# Spectroscopic Signatures of Phonon Character in Molecular Electron Spin Relaxation

**DOI:** 10.1021/acscentsci.4c01177

**Published:** 2024-12-11

**Authors:** Nathanael P. Kazmierczak, Paul H. Oyala, Ryan G. Hadt

**Affiliations:** † Division of Chemistry and Chemical Engineering, Arthur Amos Noyes Laboratory of Chemical Physics, California Institute of Technology, Pasadena, California 91125, United States

## Abstract

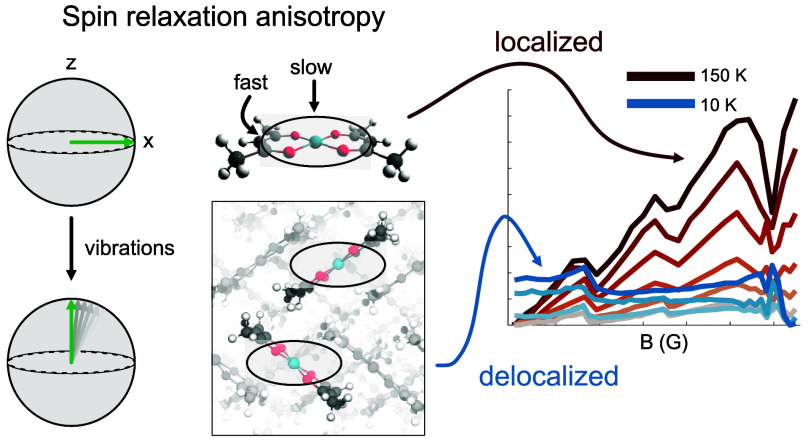

Spin–lattice relaxation constitutes a key challenge for the development of quantum technologies, as it destroys superpositions in molecular quantum bits (qubits) and magnetic memory in single molecule magnets (SMMs). Gaining mechanistic insight into the spin relaxation process has proven challenging owing to a lack of spectroscopic observables and contradictions among theoretical models. Here, we use pulse electron paramagnetic resonance (EPR) to profile changes in spin relaxation rates (*T*_1_) as a function of both temperature and magnetic field orientation, forming a two-dimensional data matrix. For randomly oriented powder samples, spin relaxation anisotropy changes dramatically with temperature, delineating multiple regimes of relaxation processes for each Cu(II) molecule studied. We show that traditional *T*_1_ fitting approaches cannot reliably extract this information. Single-crystal *T*_1_ anisotropy experiments reveal a surprising change in spin relaxation symmetry between these two regimes. We interpret this switch through the concept of a spin relaxation tensor, enabling discrimination between delocalized lattice phonons and localized molecular vibrations in the two relaxation regimes. Variable-temperature *T*_1_ anisotropy thus provides a unique spectroscopic method to interrogate the character of nuclear motions causing spin relaxation and the loss of quantum information.

## Abstract 2

Variable-temperature measurements of electron spin relaxation anisotropy reveal the character of the nuclear motions that destroy quantum information.

## 1 Introduction

Multiple emerging quantum technology concepts employ electron spins in paramagnetic molecules to store or process information.^1−3^ For example, a quantum bit (qubit) is a two-level system able to process information through uniquely quantum degrees of freedom, such as superpositions. Qubit applications include quantum sensing, computing, and communication.^4^ Paramagnetic molecules constitute a natural implementation of a qubit on the subnanometer scale, as the Zeeman sublevels of the unpaired electron spin satisfy the requirements of a two-level quantum system.^5^ Additionally, molecular spin systems exhibiting a double-well potential can store information based on which stable spin state is adopted, referred to as the concept of a single molecule magnet (SMM).^2,6,7^ In each case, information is encoded through the orientation and/or phase of the spin, so any process that dynamically alters the spin orientation on the Bloch sphere will have a deleterious impact on the proposed quantum technology.^8^

**Figure 1:**
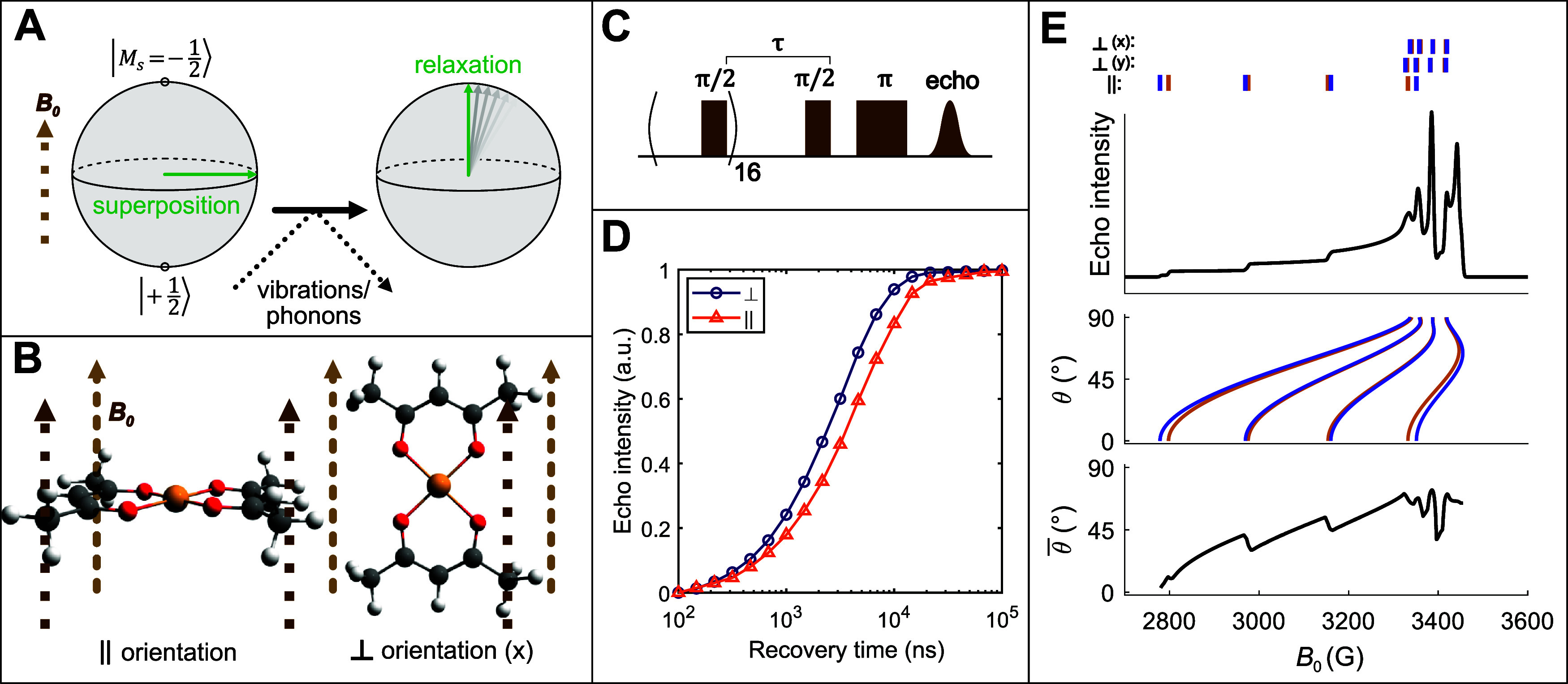
Measuring spin relaxation anisotropy. (A) Electron spin superpositions are destroyed by interactions with molecular vibrations or phonons that cause the spin to relax back to the applied magnetic field, *B*_0_. (B) Parallel (θ = 0°) and perpendicular (θ = 90°) orientations of Cu(acac)_2_ with respect to *B*_0_. θ is the angle between *B*_0_ and the normal vector of the CuO_4_ plane. (C) Saturation recovery microwave pulse sequence used to measure electron spin relaxation in pulse EPR. (D) Saturation recovery reveals distinct spin relaxation rates for the parallel and perpendicular orientations (1:1000 Cu(acac)_2_ in Pd(acac)_2_ powder, 60 K). (E) The angular dependence of the EPR spectrum enables selectivity for different orientations at different magnitudes of *B*_0_, even for a randomly oriented Cu(acac)_2_ powder. Orange ticks indicate resonance positions for the ^63^Cu isotope, while purple ticks indicate resonance positions for the ^65^Cu isotope.

Spin–lattice relaxation constitutes one such detrimental process, denoted by the time constant *T*_1_.^9^ In the molecular qubit context, this process arises when electron spins exchange energy with vibrational modes of the molecule or the surrounding bath, causing spins in superpositions to realign along the applied magnetic field and lose the quantum information (Figure 1A).^10^ Because vibrational populations increase exponentially with temperature, spin–lattice relaxation (henceforth, simply referred to as “spin relaxation”) must be minimized to obtain molecular quantum devices and sensing technologies functioning at noncryogenic temperatures.^11^

A variety of experimental investigations have sought to characterize the mechanism of molecular spin relaxation in transition metal complexes. The most common measurement has been to determine *T*_1_ by pulse electron paramagnetic resonance (EPR) at a fixed field position and analyze how it scales as a function of temperature. This can be used to extract approximate energies of the vibrational modes coupling to the spin.^12−14^ From this, spin relaxation has been suppressed by using (i) high-symmetry complexes, such as square planar Cu(II), to reduce the number of totally symmetric vibrations active for spin-phonon coupling,^15−18^ (ii) increasing the energies of those modes to reduce their thermal population,^15,19^ and (iii) reducing spin–orbit coupling through covalent metal–ligand bonding.^12,13,20^ However, temperature-dependent *T*_1_ measurements cannot unambiguously pinpoint the type of vibration driving relaxation, nor whether one or many vibrational modes dominate *T*_1_. Disagreements also exist regarding the character of the molecular vibration and/or lattice phonon modes driving relaxation.^19,21^ The challenge is that there does not yet exist a spectroscopic method for selecting a vibrational mode and measuring its contribution to *T*_1_. New spectroscopic approaches are called for to clarify the nature of spin relaxation mechanisms.

Concurrently, a plethora of theoretical studies have sought to computationally predict the rate of *S* = 1/2 spin relaxation from either experimentally calibrated models or first-principles. Such efforts have their roots in the seminal works of Van Vleck^22^ and Orbach,^23,24^ which analytically described mechanisms of relaxation. Recent works additionally incorporate the results of modern *ab initio* computational methods and quantum master equations.^25,26^ Unfortunately, contemporary studies are rife with disagreement regarding the correct spin-phonon coupling Hamiltonian to employ. There exist two main types of *S* = 1/2 spin relaxation theories: those employing the spin Hamiltonian for the coupling terms, and those not using the spin Hamiltonian approach. Of the former, there are at least four distinct models employing d^2^g_i_/dQ_2_,^27^ d**g**/dQ,^28^ dg_i_/dQ,^15,19^ and d**A**/dQ^21,29^ coupling terms. Of the latter, there are at least three proposals based on spin–orbit wave function theory,^30^ nonadiabatic spin-vibrational orbit interactions,^31^ and virtual excitations to ligand field excited states.^32^ Each choice describes the spin relaxation physics differently, consequently predicting qualitatively different vibrational modes to drive relaxation. Until a consensus is reached, it is imperative to improve these theoretical models using new types of data from experimental spectroscopy.

Recently, *T*_1_ anisotropy has been introduced as a new spectroscopic observable for probing mechanisms of spin relaxation.^30,33^ The principle of *T*_1_ anisotropy stems from the orientation dependence of spin relaxation. When measuring spin relaxation via pulse EPR, an external magnetic field (*B*_0_) is applied across the sample. *B*_0_ can have various alignments with respect to individual molecules analyzed. For a square-planar Cu(II) complex, such as copper(II) bis(acetylacetonate) (Cu(acac)_2_), alignment of *B*_0_ passing at a right angle through the plane is referred to as the parallel orientation (θ = 0°) (Figure 1B). Conversely, *B*_0_ contained within the plane is referred to as the perpendicular orientation (θ = 90°). If *T*_1_ is measured for molecules at these orientations, different values may generally be obtained (Figure 1C,D). This is the core concept of *T*_1_ anisotropy. Furthermore, a field-swept EPR spectrum will often contain natural statistical selectivity for different orientations at different magnetic field positions (Figure 1E), so *T*_1_ anisotropy information can be collected even from randomly oriented powder samples. Analysis of *T*_1_ anisotropy has provided insight on how chemical bonding affects spin dynamics^30^ and pinpointed the types of vibrations causing relaxation in *S* = 1/2 versus *S* = 1 molecular qubits.^33^

Here, we conduct a two-dimensional profiling of changes in *T*_1_ by systematically altering both temperature and field, producing a full matrix of *T*_1_ data along two independent axes (Figure S18). The field dimension yields anisotropy information at each temperature probed. We refer to this approach as variable-temperature, variable-field *T*_1_ anisotropy (VTVH-*T*_1_). We apply the VTVH-*T*_1_ methodology to Cu(acac)_2_ and copper octaethylporphyrin (CuOEP), both of which have been studied as molecular qubit candidates.^19,30^ For powder samples of both compounds, the shape of the *T*_1_ anisotropy changes between the high- and low-temperature limits, which enables assignment of different regimes of spin relaxation within a given compound. We show this information cannot be extracted in general from the temperature scaling of fixed-field *T*_1_ measurements alone. Single-crystal VTVH-*T*_1_ measurements further reveal that spin relaxation rates orient along the molecular axes at high temperatures, but switch to crystal lattice plane axes at low temperatures. This enables VTVH-*T*_1_ to ascertain the localized vs delocalized character of the vibrational modes driving spin relaxation. VTVH-*T*_1_ provides a strikingly direct experimental portrait of spin relaxation mechanisms.

## 2 Results

### 2.1 Powder VTVH-*T*_1_

**Figure 2:**
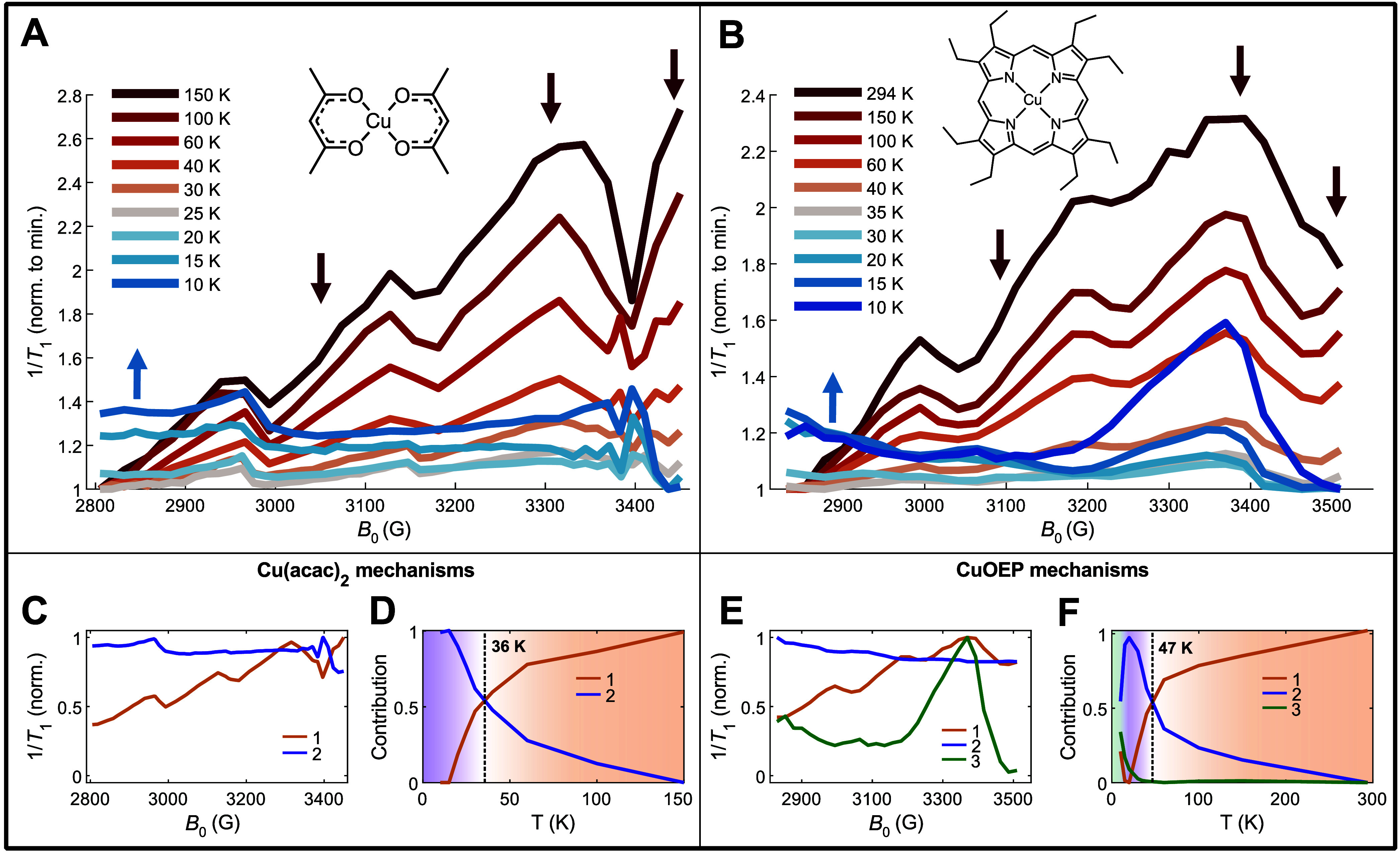
Variable-temperature, variable-field *T*_1_ anisotropy (VTVH-*T*_1_). (A) VTVH-*T*_1_ for 1:1000 Cu(acac)_2_ in Pd(acac)_2_ powder sample. Red and blue arrows indicate decreasing and increasing anisotropy as temperature is reduced, respectively. Anisotropy traces at each temperature are normalized to the slowest relaxation rate at any field position. (B) VTVH-*T*_1_ for 1:100 CuOEP in ZnOEP powder sample. (C,D) Bilinear factor analysis decomposition (Supporting Information section 8) of the Cu(acac)_2_ anisotropy data. (C) Two anisotropy patterns are extracted from the Cu(acac)_2_ data. (D) Associated temperature dependence of the anisotropy patterns in panel C, corresponding to Cu(acac)_2_ relaxation mechanism contributions. (E) Three anisotropy patterns are extracted from the CuOEP data. (F) Associated temperature dependence of the anisotropy patterns in panel E, corresponding to CuOEP relaxation mechanism contributions.

We began by acquiring X-band pulse saturation recovery VTVH-*T*_1_ measurements on randomly oriented powder samples of 1:1000 Cu(acac)_2_ in Pd(acac)_2_ and 1:100 CuOEP in ZnOEP (Figure 2A,B). *T*_1_ was acquired at a minimum of 25 field positions for each temperature and 10 temperature points (Supporting Information section 13). Previously, CuOEP temperature-dependent *T*_1_ has been measured by inversion recovery from 6 to 294 K without analysis of anisotropy,^19^ while Cu(acac)_2_ anisotropy has been measured by inversion recovery at 20, 40, and 100 K without report of a temperature-dependent *T*_1_ curve.^30,33^ The use of saturation recovery in the present work is important to reliably analyze low-temperature VTVH-*T*_1_ without conflation of anisotropy and spectral diffusion (Supporting Information sections 4 and 6). Complete saturation was not obtained under the fastest-relaxing temperature points (150 K for Cu(acac)_2_ and 294 K for CuOEP), so inversion recovery was employed instead. Spectral diffusion is not anticipated to be an issue in the presence of fast spin–lattice relaxation. *T*_1_ scales steeply with temperature, but changes in the anisotropy can be visualized by normalizing the data at each temperature to the slowest relaxation rate. This normalizes to the isotropic portion of the spin relaxation and enables comparison of the fraction of *T*_1_ that is anisotropic at each temperature. Note that this normalization procedure removes the thermal dependence of the average *T*_1_, so these anisotropy data provide independent information from that of traditional fixed-field temperature-dependent *T*_1_ experiments.

Cu(acac)_2_ does not display a quantifiable spin echo above 150 K, while CuOEP is room-temperature coherent via EPR, as observed previously.^19^ At the highest temperature of 150 K, Cu(acac)_2_ displays a sawtooth linear increase in 1/*T*_1_ from low fields (parallel orientations) to high fields (perpendicular orientations), characteristic of a sin^2^θ *T*_1_ anisotropy pattern for this molecule (Figure 2A, Figure S25).^30^ This anisotropy form has been previously assigned to the impact of totally symmetric metal–ligand bond stretching vibrations.^30^ The shape of the anisotropy remains constant with decreasing temperature from 150 to 25 K. However, the proportional contribution of the *T*_1_ anisotropy to the total *T*_1_ decreases significantly, from a maximum *T*_1_ anisotropy ratio of 2.7 at 150 K to a minimum of 1.2 at 25 K. At 20 K and below, a new anisotropy shape grows in, with slowest relaxation at the highest fields and comparatively fast relaxation at the parallel orientation. The central hyperfine sawtooth discontinuity at 3150 G vanishes by 10 K, while the discontinuity at 2970 G becomes more prominent at 10 K, and the sharp patterns above 3350 G additionally change shape. Analogous changes are visible for CuOEP, where the sin^2^θ anisotropy pattern (Figure S37) decreases from 294 to 35 K, and new shapes arise at 30 K and below (Figure 2B).

Visual inspection of a given anisotropy data set (Figure 2A,B) indicates that only a small number of fundamental *T*_1_ anisotropy patterns constitute the data, but the relative contributions of these patterns vary in different ways as a function of temperature. Thus, these anisotropy patterns likely arise from multiple underlying mechanisms of spin relaxation with distinct thermal dependencies, arising from the impact of different classes of phonons. We sought to quantify the relative proportions of each anisotropy pattern to extract mechanistic insight. Unlike in previous *T*_1_ anisotropy studies,^30,33^ however, a simple parametric form for *T*_1_ as a trigonometric function of the angle θ could not be found for the low-temperature shapes.

We therefore employed a factor analysis procedure based on soft modeling to deconvolute the contributions of the distinct anisotropy patterns (Supporting Information section 8).^34−37^ The bilinear factor analysis decomposition represents the primary anisotropy data (Figure 2A,B) as the sum of fundamental anisotropy patterns (Figure 2C,E) that each possess their own unique temperature-dependent contributions (Figure 2D,F) subject to physical constraints like nonnegativity. This procedure is equivalent to a matrix factorization (Figure S17) and is sometimes referred to as model-free global analysis. Each fundamental anisotropy pattern spans across the full range of *B*_0_ values. Thus, the temperature-dependent contributions track the evolution of the entire normalized anisotropy shape, which is different than extracting the temperature-dependent variation of *T*_1_ at a fixed field. Likewise, the anisotropy patterns are extracted from over the entire range of temperature values. By extracting anisotropy information from the *B*_0_ dimension, VTVH-*T*_1_ provides independent information on spin relaxation not accessed in a fixed-field temperature-dependent *T*_1_ experiment.

Applying this scheme to Cu(acac)_2_ successfully separates the two anisotropy patterns visible in the data. Relaxation mechanism #1 corresponds to the sin^2^θ anisotropy pattern (Figure 2C). The high-temperature mechanism #1 is shown to be more anisotropic than the low-temperature mechanism #2, consistent with the magnitudes of the anisotropies visible in the data. The crossover point between the two relaxation mechanisms is found to arise at 36 K (Figure 2D). CuOEP displays three distinct anisotropy patterns (Figure 2E), owing to the prominent bulge at 3350 G below 20 K (Figures S31–S36). The high-temperature pattern is dominant above 47 K (Figure 2F), a higher crossover point than observed for Cu(acac)_2_. In summary, powder VTVH-*T*_1_ indicates a low-temperature and a high-temperature regime of spin relaxation for both Cu(acac)_2_ and CuOEP, demarcated by the 36 and 47 K crossover points for each.

At this stage of the analysis, the different regimes can be assigned to relaxation dominated by different classes of phonons. Each phonon mechanism possesses its own characteristic anisotropy pattern, and the temperature dependences arise from thermal population of the relevant phonon modes. However, two important questions remain. First, does anisotropy give different information about relaxation mechanisms compared to traditional fixed-field temperature-dependent *T*_1_ fitting? Second, can anisotropy extract any unique experimental information regarding the characteristics of the phonons participating in each relaxation mechanism? Both questions are answered affirmatively in the following sections.

### 2.2 Comparison to Temperature-Dependent *T*_1_ Fitting

**Figure 3:**
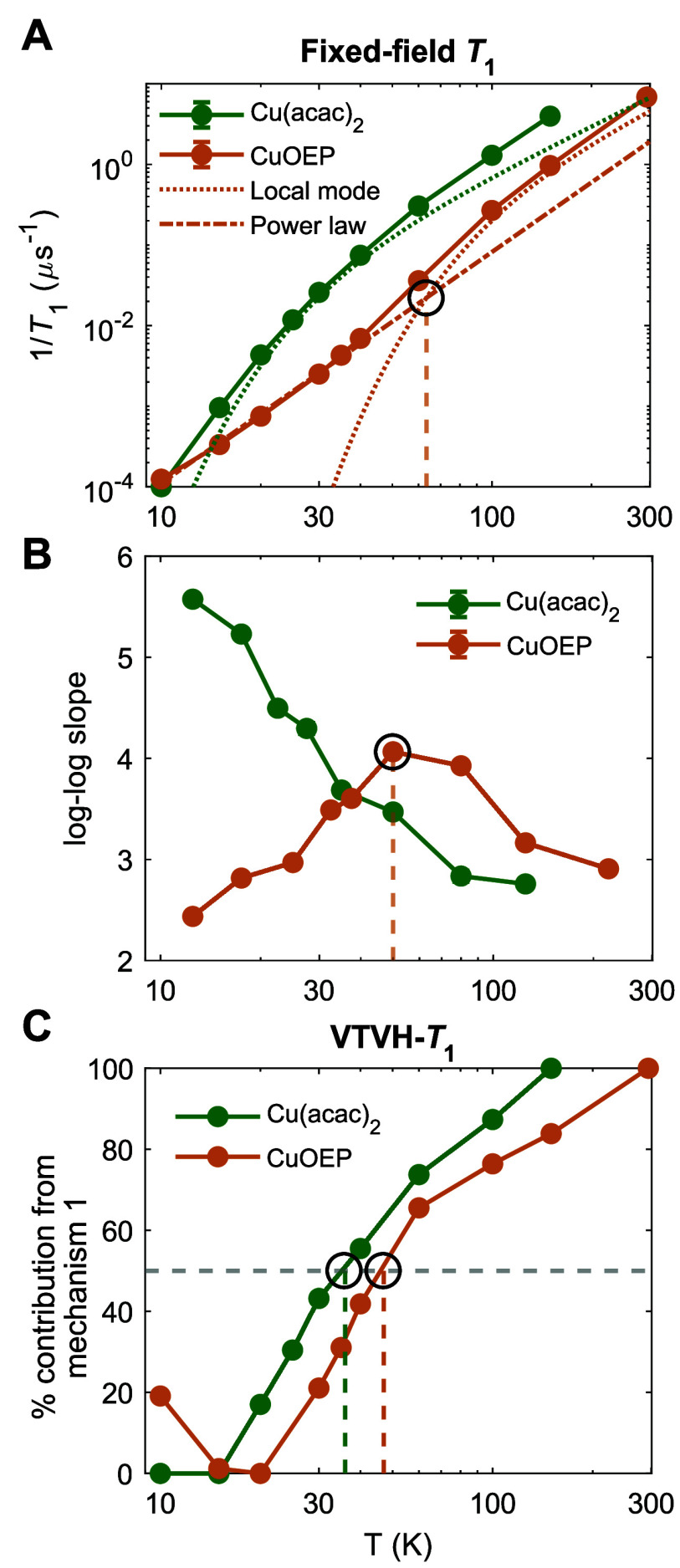
Comparison to local mode fitting. (A) Local mode fitting of temperature-dependent *T*_1_ at fixed field positions selective for the perpendicular orientation (Cu(acac)_2_: 3318 G, 9.7060 GHz; CuOEP: 3369 G, 9.6291 GHz). CuOEP is fit to two spin relaxation mechanisms, while Cu(acac)_2_ can only be fit to one. (B) The slope of the graph in panel A indicates the power law scaling of 1/*T*_1_ vs *T* at all temperatures, revealing two regimes for CuOEP but only one for Cu(acac)_2_. (C) Percentage contribution of the high-temperature spin relaxation mechanism from VTVH-*T*_1_ indicates two distinct regimes for both Cu(acac)_2_ and CuOEP, despite the absence of clear features in 1/*T*_1_ vs *T* for Cu(acac)_2_.

Assignment of direct, Raman, and local mode relaxation processes is commonly conducted by fitting the temperature scaling of *T*_1_ at a fixed *B*_0_ to power law and local mode functional forms.^8,12^ We sought to compare these assignments to the spin relaxation regimes extracted from powder VTVH-*T*_1_. Local mode fits to CuOEP saturation recovery data reveal two distinct contributions to the temperature scaling of *T*_1_: a power law process dominant at low temperatures, and a molecular vibration dominant at high temperatures (Figure 3A, Supporting Information section 5). The fits are in good agreement with a previous report employing inversion recovery data.^19^ The crossover between the two contributions occurs at 64 K. This is reasonably close to the 47 K mechanism crossover observed by CuOEP VTVH-*T*_1_ (Figure 2F), suggesting that the mechanism crossover detected is likely the same in both the temperature dimension and the anisotropy dimension.

However, the Cu(acac)_2_*T*_1_ temperature scaling cannot be fit to two functional forms (Figure 3A). Unlike for CuOEP, the slope of 1/*T*_1_ vs T is steeper at lower temperatures than at higher temperatures. This precludes use of a power law to fit the low-temperature data, which would dominate *T*_1_ over the entire range and predict excessively fast spin relaxation at high temperature. A single local mode may be fit, but it does not satisfactorily capture the curvature of the data (Supporting Information section 5). Fitting two or more local mode forms is of course mathematically possible, but the relationship to real mechanistic regimes of spin relaxation would be spurious, given the single curvature of the data.

Analysis of *T*_1_ vs T without functional fitting also fails to detect two mechanistic regimes for Cu(acac)_2_. The curvature may be easily visualized through plotting the slope of log(1/*T*_1_) vs log(T) (Figure 3B).^15,19^ The CuOEP slope displays a maximum around 50 K that indicates a clear separation between two mechanistic regimes, while Cu(acac)_2_ displays a monotonic decrease lacking clear features. We also acquired inversion recovery measurements of 1/*T*_1_ vs T for Cu(acac)_2_ with finer temperature resolution, yet no mechanistic separation could be ascertained (Figures S8 and S9, Supporting Information Sections 3–4). A similar phenomenon was described in a recent report of spin relaxation in vanadyl tetrapyrazinoporphyrazine dyes (VOPyzPz-DIPP), where elaboration with peripheral substituents removed a visible mechanistic separation from 1/*T*_1_ vs T.^38^ Yet, as demonstrated, powder VTVH-*T*_1_ measurements are able to unveil multiple mechanistic regimes for both Cu(acac)_2_ and CuOEP (Figure 3C). Thus, the anisotropy information in VTVH-*T*_1_ contains unique mechanistic insights not present in the traditional 1/*T*_1_ vs T fitting approach.

### 2.3 Single-Crystal *T*_1_ Anisotropy

We then sought to extract information about the character of the dominant vibrational mode in the different mechanistic regimes. While the high-temperature anisotropy shape for both Cu(acac)_2_ and CuOEP can be nicely fit to the sin^2^θ form, the low-temperature shapes cannot easily be fit to a function of θ. Maximal care was taken to exclude spectral diffusion^10,39−41^ as the source of these shapes, including probing the impact of the number of pulses, interpulse spacing, and pulse duration in the picket fence saturation recovery sequence (Figure S15), as well as the Cu(acac)_2_ paramagnetic concentration dependence of the 20 K powder anisotropy shape (Figure S16). We conclude spectral diffusion is unlikely to be the cause of these powder anisotropy shapes.

**Figure 4:**
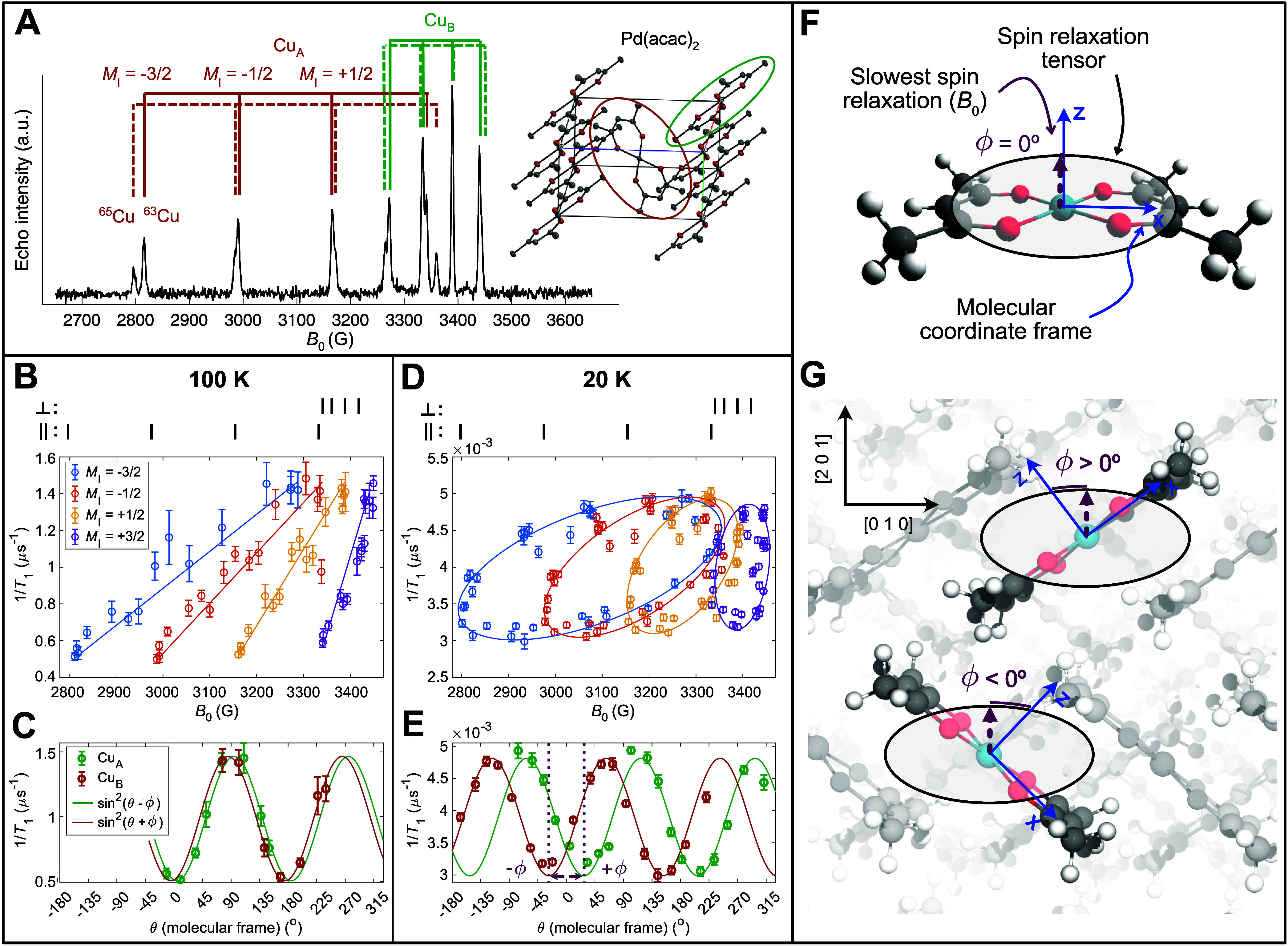
Single-crystal *T*_1_ anisotropy for 1:1000 Cu(acac)_2_ in Pd(acac)_2_. (A) Example single-crystal EDFS at 100 K, together with assignments of the peaks to the two sites in the unit cell. (B) 100 K spin relaxation rate vs *B*_0_ indicates linear trends between parallel and perpendicular positions for each hyperfine manifold. Lines serve as a guide to the eye. (C) 100 K relaxation (*M*_I_ = −3/2) vs molecular orientation reveals sin^2^θ angular dependence, with the slowest relaxation very close to the pure parallel position (θ = 5°). (D) 20 K relaxation vs *B*_0_ displays elliptical *T*_1_ patterns for each hyperfine manifold. (E) 20 K relaxation (*M*_I_ = −3/2) vs field displays sin^2^(θ ± ϕ) angular dependence, with a large phase shift inducing slowest spin relaxation away from the parallel orientation (θ = 28°). (F) *T*_1_ anisotropy determined by the local orientation of the molecular point group leads to sin^2^θ angular dependence, characteristic of localized spin-phonon coupling at 100 K. (G) *T*_1_ anisotropy oriented along a lattice plane of the crystal unit cell can lead to sin^2^(θ ± ϕ) angular dependence with different phase shifts ϕ for each crystallographic site, characteristic of delocalized spin-phonon coupling at 20 K.

To obtain the most detailed analysis of *T*_1_ anisotropy, a single crystal of Cu(acac)_2_ cocrystallized with Pd(acac)_2_ in a 1:1000 ratio was prepared. The crystal was face-indexed by X-ray diffractometry and mounted for rotation along the [1 0 –1] axis to access pure parallel and perpendicular positions (Supporting Information section 9). A slight rhombic splitting exists between *g*_*x*_ and *g*_*y*_.^30^ DFT calculations of the *g*-tensor indicate the rotation about [1 0 –1] specifically accesses the *g*_*x*_ position, the smaller of the two perpendicular *g* values (Supporting Information section 12). Single-crystal *T*_1_ anisotropy experiments offer three significant advantages: (1) the ability to know the exact 360° molecular orientation through laboratory frame rotations, rather than inferring an angle between 0° and 90° from the resonant field position, (2) the ability to selectively probe different hyperfine transitions that would overlap in the powder spectrum, and (3) a vastly altered, sparse spectral density of states. The latter should reduce or eliminate spectral diffusion. Echo-detected field sweep (EDFS) line widths as sharp as 10 G were obtained for the doped crystal (Figure 4A), additionally enabling selective *T*_1_ measurements on ^65^Cu and ^63^Cu nuclear isotopes. Two metal sites with different molecular orientations exist in the isostructural Pd(acac)_2_ and Cu(acac)_2_ unit cells. Thus, two distinct Cu(II) hyperfine-split signals are observed at almost every crystal orientation, which we denote Cu_A_ and Cu_B_. An exception exists when the two molecules possess the same relative angle to the applied magnetic field, in which case the Cu_A_ and Cu_B_ resonances coincide. The Cu_A_ and Cu_B_ sites are equivalent by symmetry, related by a 2-fold screw axis along the *b* direction of the P2_1_/n space group.

Saturation recovery measurements for all ^63^Cu peaks were acquired as a function of crystal orientation. At 100 K, 1/*T*_1_ varies almost linearly with *B*_0_ for each hyperfine manifold (Figure 4B). For a given orientation, the relaxation rates are nearly equivalent for different values of *M*_I_ and for the ^63^Cu and ^65^Cu isotopes, despite the differing nuclear gyromagnetic ratios of the latter. The insensitivity to nuclear spin parameters indicates that Cu(II) spin relaxation at 100 K does not proceed through modulation of the hyperfine tensor, consistent with previous experimental^42^ and theoretical^15,30^ reports. By instead plotting 1/*T*_1_ vs the molecular frame θ, a clear sin^2^θ dependence of spin relaxation is observed (Figure 4C), with slowest relaxation very close to the exact parallel position (θ = 5°). This is fully consistent with the interpretation of the high-temperature powder anisotropy form for Cu(acac)_2_.

A surprise arose when conducting this analysis at 20 K, which is within the low-temperature mechanism regime extracted from powder VTVH-*T*_1_ (Figures 2 and 3). Unlike previous observations of *T*_1_ anisotropy, a plot of 1/*T*_1_ vs *B*_0_ displays the opening of tilted ellipses (Figure 4D), where the semimajor axis sits approximately along a linear variation from parallel to perpendicular orientations. Widened ellipses are also visible at 10 K (Figure S63). The 20 K data has been reproduced with two separate crystals mounted in the pulse EPR instrument with two different sample mounting procedures (Supporting Information section 9). The implication of an ellipse is that two distinct Cu(acac)_2_ orientations possessing the same resonant field *B*_0_ nonetheless have different spin relaxation rates. Since the resonant field *B*_0_ is invariant to changes in the sign of θ, this could occur if orientations of +θ and –θ have different relaxation rates. Indeed, upon plotting 1/*T*_1_ vs θ (the angle of *B*_0_ to the molecular frame *z*-axis), the spin relaxation rate is not invariant to changes in the sign of θ for any particular Cu site (Figure 4E). Both Cu_A_ and Cu_B_ can be described instead by a phase-shifted sin^2^θ, given as sin^2^(θ ± ϕ). The phase shift ϕ is equal and opposite for the two sites, and equal to 28° at 20 K. Thus, slowest spin relaxation for any given Cu(acac)_2_ molecule arises at either +28° or −28°, and *not* at the molecular frame parallel orientation.

## 3 Discussion

We interpret the single-crystal *T*_1_ anisotropy data by proposing the concept of a “spin relaxation tensor.” Just as the *g*-tensor indicates how the Zeeman splitting changes as the magnetic field rotates relative to the molecular frame, so too the spin relaxation tensor indicates how the spin relaxation rate changes as the magnetic field rotates relative to the molecular frame. A formal definition of the spin relaxation tensor is given in Supporting Information Section 10. The *g*-tensor has principal axes, which indicate the orientations of largest and smallest Zeeman splitting. Likewise, the spin relaxation tensor has principal axes, which indicate the fastest and slowest spin relaxation rates.

Crucially, the principal axes of the spin relaxation tensor may or may not align with the molecular frame coordinate system (Figure 4F,G). The implications of tensor (mis-)alignment on the *T*_1_ anisotropy can be illustrated graphically, where the spin relaxation tensor is visualized as an ellipse. The ellipse represents the different possible orientations of the applied field *B*_0_, and the distance from the center of the ellipse represents the rate of spin relaxation at that orientation. At 100 K, the spin relaxation tensor aligns to the coordinate frame of the molecular point group, and the orientation of slowest spin relaxation coincides with the molecular *z*-axis (Figure 4F). This indicates that the dominant spin-phonon coupling process at 100 K is localized on individual molecules. At 20 K, by contrast, the spin relaxation tensor aligns to lattice planes in the crystal space group. In this scenario, the orientation of slowest spin relaxation no longer coincides with the molecular *z*-axis, but with the orientation of the crystal lattice planes, and equal-and-opposite phase shifts (ϕ) may be obtained due to the angular orientation between the two Cu(acac)_2_ molecules (Figure 4G). This behavior matches the experimental relaxation data at 20 K (Figure 4E), and a simple analytical model successfully predicts the observed sin^2^(θ ± ϕ) *T*_1_ anisotropy form (eq S9). Plots of 1/*T*_1_ vs the laboratory frame orientation of the crystal (Ω) confirm that Ω does not determine 1/*T*_1_ at 100 K, while it partially determines 1/*T*_1_ at 20 K, and completely determines 1/*T*_1_ at 10 K, consistent with a dominant effect of the lattice orientation at low temperatures (Figures S56–S58). Because the spin relaxation tensor responds to the intermolecular crystal packing rather than the intramolecular bonding, this indicates that the dominant spin-phonon coupling process at 20 K is delocalized across multiple molecules.

The localized (100 K) and delocalized (20 K) phonon assignments obtained by single-crystal *T*_1_ anisotropy correspond to the high- and low-temperature spin relaxation regimes obtained from powder VTVH-*T*_1_ (Figure 2C–F). Therefore, the impact of different atomic motions can be disentangled based on their VTVH-*T*_1_ spectroscopic signatures in the single-crystal and powder forms. The VTVH-*T*_1_ methodology provides a uniquely direct handle for measuring the vibrational character of competing spin relaxation mechanisms.

If the spin relaxation tensor does not align with the molecular axes at low temperature, then there is also no reason to assume that it is axial (like the molecular *g*-tensor), or that any of its principal axes lie in the (1 0 –1) rotation plane interrogated in the single crystal experiments. While Figure 4F,G has depicted the spin-relaxation tensor as an ellipse in two-dimensions, it is actually a three-dimensional surface. Rotation of the crystal along multiple axes would enable characterization of the full tensor, so there are no limitations in principle for less-than-axial systems. However, this is beyond the scope of the present study. We note that the [1 0 –1] axis also produces a small rotation in the molecular xy plane in addition to the xz plane, but xy *T*_1_ anisotropy is unlikely to explain the observed phase shifts (Figures S54–S55). The three-dimensional structure of the spin relaxation tensor would likely be required to simulate the low-temperature powder anisotropy shape.

The insights from single-crystal VTVH-*T*_1_ anisotropy open up intriguing crystal engineering approaches for controlling lattice phonon involvement in spin relaxation. Changing the space group will probably change the observed single-crystal *T*_1_ anisotropy significantly. Crystallization of a paramagnetic analyte in different diamagnetic host polymorphs or compounds may alter the phase shift properties in the low-temperature regime. We note that single-crystal *T*_1_ anisotropy measurements at very low temperature (often <5 K) were reported on several inorganic lattices in the early days of EPR, including for Cr^3+^ ions in ruby,^9,43^ as an experimental technique for detecting cross relaxation,^9^ and in lanthanide-doped materials.^44,45^ However, inorganic lattices lack the potential for tuning *T*_1_ through intermolecular contacts. We are aware of only one comparable single-crystal *T*_1_ anisotropy study for a highly coherent molecular crystal, reported by Eaton and Eaton in 1995, which examined Cu(dtc)_2_ in Ni(dtc)_2_ and Zn(dtc)_2_ hosts.^46^ The 100 K anisotropy in the isostructural Ni(dtc)_2_ host agreed well with the patterns displayed by Cu(acac)_2_ at 100 K (Figure 4B), but it is unknown if Cu(dtc)_2_ also displays phase shift behavior at lower temperatures. Alteration of molecular packing through peripheral substituents may further probe the delocalized spin-phonon coupling regime–this effect may already be at play in the aforementioned VOPyzPz-DIPP system.^38^ Finally, application of the VTVH-*T*_1_ methodology to qubits embedded in metal–organic frameworks may provide a tunable means for engineering lattice phonon contributions in a high-symmetry environment.^47,48^

Powder VTVH-*T*_1_ anisotropy contains broad potential to elucidate spin relaxation regimes not accessible by conventional local mode fitting. In the above analysis (Figure 2C–F), Cu(acac)_2_ spin relaxation was shown to be dominated by a molecular vibration mechanism down to lower temperatures (>36 K) as compared to CuOEP (>47 K). This result is in good agreement with the ligand field spin dynamics model.^15^ By density functional theory (DFT), Cu(acac)_2_ has a lowest-energy totally symmetric vibration^30^ at 212 cm^–1^, whereas the corresponding vibration for CuOEP exists at 271 cm^–1^ by resonance Raman spectroscopy.^19^ This change arises from having two bidentate ligands in Cu(acac)_2,_ which enables a low-energy totally symmetric scissoring mode that is not present in CuOEP.^15,30^ The change in mechanism crossover point between CuOEP and Cu(acac)_2_ likely arises from the increased thermal population of this mode in Cu(acac)_2_. Discrimination between the impact of localized and delocalized phonons affects the design criteria for suppressing spin relaxation. If spin relaxation dominantly proceeds through localized modes, then the first coordination sphere of the molecule should be altered to suppress relaxation. Relevant strategies have been described in the literature, including stiffening the ligand framework to raise the metal–ligand stretching frequencies and strengthening the ligand field to reduce the orbital contribution to the *g* value.^20^ Conversely, if delocalized modes dominantly drive spin relaxation, then intermolecular contacts/crystal packing effects should exhibit a greater impact. Relevant design criteria are less-well characterized, but may include the symmetry of the space group and the rotational freedom of the molecule in the lattice.^33^ Powder VTVH-*T*_1_ anisotropy can thus produce chemically interpretable design principles for controlling spin relaxation.

The present data invite detailed theoretical work to explain the change in coupling mechanism between the low- and high-temperature regimes, particularly as regards to the origin of the single-crystal phase shift. Initially, it might seem that a delocalized lattice phonon should still induce spin relaxation anisotropy obeying the molecular symmetry. Under the spin–orbit wave function model, *T*_1_ anisotropy has been shown to arise from anisotropic spin–orbit coupling to ligand field electronic excited states.^30^ This is a local property of the first coordination sphere, regardless of how delocalized the vibrational mode is. Alternatively, the delocalized phonons could induce spin flips involving multiple Cu centers (e.g., cross relaxation,^9,48^ or dipole–dipole mediated relaxation^29^). But this seems unlikely in view of the minimal paramagnetic concentration dependence of the Cu(acac)_2_ 20 K powder anisotropy (Figure S16), as well as the dilute nature of the single-crystal samples. *T*_1_ anisotropy has also recently been predicted through a nonadiabatic spin-vibrational orbit mechanism, though the predicted magnitude is orders of magnitude larger than observed experimentally.^31^ The present anisotropy phenomena will thus provide a stringent test for development of improved spin relaxation theories.

Very recently, a new *ab initio* model has proposed that *S* = 1/2 spin relaxation occurs through virtual excitations to ligand field excited states.^32^ Similar to the previously reported spin–orbit wave function model,^30^ this approach employs explicit anisotropic spin–orbit couplings to excited states, enabling accurate *ab initio* prediction of *T*_1_ anisotropy within an open quantum systems framework. The model displays significantly more promising agreement with experiment than previous *ab initio* theories based on the spin Hamiltonian, including invariance to metal-based hyperfine coupling and the magnitude of the external magnetic field. However, one key point of disagreement with established experimental interpretation remains: the *ab initio* results were used to argue against the impact of high-energy molecular vibrations in driving spin relaxation. These vibrations have been experimentally interpreted to give rise to the curved local mode functional form in a plot of 1/*T*_1_ vs T, such as that observed for CuOEP (Figure 3A). We note that the Cr(V) complexes employed in the *ab initio* predictions do not display clear local mode curvature–they behave more like Cu(acac)_2_ than CuOEP in the present study. It is possible that only high-symmetry D_4h_ and C_4v_ compounds regularly display a dominant impact of a local mode, a result that would be consistent with previously derived group theory selection rules for spin-phonon coupling.^15^ Nevertheless, the present VTVH-*T*_1_ data show that Cu(acac)_2_ and CuOEP alike display two distinct regimes of spin relaxation. For both compounds, this is most easily explained by high-energy molecular vibrations playing a major role at elevated temperatures. Therefore, accurate prediction of both temperature- and orientation-dependence in VTVH-*T*_1_ constitutes an important test for *ab initio* spin dynamics models, which would be required to assert the dominant impact of low-energy phonons across all temperature regimes.

## 4 Conclusion

The VTVH-*T*_1_ methodology uniquely probes the character of nuclear motions involved in spin relaxation and, thus, the loss of quantum information. The new mechanistic understanding gained from VTVH-*T*_1_ will provide useful design principles for controlling spin relaxation across temperature regimes that differ by orders of magnitude, as well as diverse applications in molecular quantum information science ranging from quantum computing to room-temperature quantum sensing. Powder VTVH-*T*_1_ measurements delineate multiple spin relaxation mechanisms operating in the same compound at different temperatures. Single-crystal VTVH-*T*_1_ measurements characterize the orientation of the spin relaxation tensor, garnering insight into the localized vs delocalized character of the vibrational/phonon modes coupled to the spin. The two-dimensional nature of the VTVH-*T*_1_ methodology has proven crucial for extracting this information. The temperature dimension incorporates all the information attainable from functional fitting of 1/*T*_1_ vs T, while the field dimension incorporates all the information attainable from *T*_1_ anisotropy. Therefore, VTVH-*T*_1_ provides the most complete picture of spin relaxation yet obtained from pulse EPR.

Until now, there has been no experimental spectroscopic method able to directly interrogate the character of the phonons causing spin relaxation. Information about spin relaxation has only been indirectly inferred from the temperature dependence of *T*_1_. Yet, different phonons produce distinct anisotropy signatures, which can be used to directly detect competing spin relaxation mechanisms. By leveraging this insight, we constructed a direct spectroscopic probe for phonon character in spin relaxation. We believe this VTVH-*T*_1_ approach will constitute the new gold standard for mechanistic studies of spin relaxation in molecular qubits.

## Supporting Information Available

Sample preparation, pulse EPR methods, temperature dependent *T*_1_ and *T*_m_ curves for Cu(acac)_2_, comparison of *T*_1_ for differing pulse sequences, *T*_1_ local mode fitting, influence of saturation recovery pulse sequence parameters on *T*_1_ anisotropy, influence of paramagnetic concentration on *T*_1_ anisotropy, factor analysis methods, single-crystal pulse EPR methods, computational methods, tabulation of *T*_1_ anisotropy data, example Matlab code for VTVH-*T*_1_ factor analysis, supplementary references (PDF)
Transparent Peer Review report available (PDF)
