# Supplementary material for: Spectroscopic Signatures of Phonon Character in Molecular Electron Spin Relaxation
Source: ACS Cent Sci. 2024 Dec 11;10(12):2353–62. doi: 10.1021/acscentsci.4c01177 (PMC11672536; doi:10.1021/acscentsci.4c01177)
Supplement: Supplementary file 1 — oc4c01177_si_001.pdf [file oc4c01177_si_001.pdf]

Supporting Information

Spectroscopic Signatures of Phonon Character in Molecular Electron Spin Relaxation

Nathanael P. Kazmierczak[1], Paul H. Oyala[1], and Ryan G. Hadt*[,1]

[1]Division of Chemistry and Chemical Engineering, Arthur Amos Noyes Laboratory of Chemical Physics, California Institute of Technology, Pasadena, California 91125, United States

*Corresponding Author: rghadt@caltech.edu

1. Sample preparation. 2

2. Pulse electron paramagnetic resonance spectroscopy 2

3. Temperature-dependence of T_1 and T_m for $Cu(acac)_2$ 6

4. Comparison of T_1 temperature dependence for differing pulse sequences 10

5. T_1 local mode fitting 12

6. Influence of saturation recovery pulse sequence parameters on powder T_1 anisotropy 15

7. Influence of paramagnetic concentration on powder T_1 anisotropy 16

8. VTVH-T_1 powder anisotropy factor analysis 18

9. Single crystal pulse electron paramagnetic resonance 30

10. Discussion of the spin relaxation tensor 42

11. Single-crystal anisotropy at 10 K 46

12. Computational methods 47

13. Tabulation of powder T_1 anisotropy data 49

14. Tabulation of single-crystal T_1 anisotropy data 65

15. Matlab code for VTVH-T_1 powder anisotropy factor analysis 72

16. References 82

1. Sample preparation.

Powder samples of 1:1000 $Cu(acac)_2$ in $Pd(acac)_2$ and 1:100 CuOEP in ZnOEP were prepared by rotary evaporation from stoichiometric solutions as described previously.[1,2] Both compounds possess planar geometries in the crystal structure. Single-crystal samples of 0.1% $Cu(acac)_2$ co-crystallized into $Pd(acac)_2$ were prepared by vapor diffusion of diethyl ether into a chloroform solution of the components mixed in stoichiometric ratios. Face indexing was conducted on a Bruker AXS D8 VENTURE KAPPA diffractometer coupled to a PHOTON II CPAD detector with Kα radiation (λ = 1.54178 Å) from an IμS micro-source. The unit cell agreed with the reported $Pd(acac)_2$ structure.[3] The dimension of the crystal employed for the main text EPR analysis was 3 mm x 1.5 mm x 0.2 mm (**Figure S43**).

A series of powder samples with different paramagnetic concentrations was prepared for $Cu(acac)_2$ in $Pd(acac)_2$, encompassing 1:10, 1:30, 1:100, 1:300, 1:1000, 1:3000, and 1:10000 concentrations. The doubly-integrated intensities of the CW EPR signals displayed a good correlation with the nominal concentrations, suggesting that the true paramagnetic concentrations are similar to the solution stoichiometry. Given that the powder samples are prepared by fast rotary evaporation, it is to be expected that the paramagnetic centers will be well dispersed in the diamagnetic host as a chemical dilution.

For the single crystal sample, it is possible that the paramagnetic concentration could differ from the nominal stoichiometry due to different relative solubility of the paramagnetic and diamagnetic compounds. If anything, slow co-crystallization of a mixture with a small amount of paramagnetic dopant is likely to exclude the dopant, due to greater fractional solubility. This would tend to decrease the paramagnetic concentration of the single crystal. As spin dynamics in the paramagnetically dilute regime are independent of paramagnetic concentration, the single crystal results are unlikely to be perturbed by off-stoichiometry compositions.

The paramagnetic concentration of the single crystal versus the powder concentration series can be roughly estimated by the lineshapes of the pulse echo-detected field sweep (EDFS). Changes in the peak width in the EDFS for the powdered samples indicates the degree of dipolar broadening to be expected at a given paramagnetic concentration. As shown in **Figure S16D**, the narrow powder line peak at 3386 G exhibits a full-width-at-half-max of about 8.5 G for the 1:3000 and 1:10000 dilution levels. This peak broadens to 9.5 G at 1:300, and 13 G at 1:100. By contrast, the single crystal sample possesses a 5 G FWHM for the sharp line centered at 3390 G **(Figure 4A**), indicating that it is unlikely to be as concentrated as the broadened 1:100 sample. Thus, the single crystal EPR signal is consistent with the nominal single crystal stoichiometry of 1:1000.

2. Pulse electron paramagnetic resonance spectroscopy

Pulse X-band EPR experiments were conducted with a Bruker ELEXSYS E580 pulse EPR spectrometer using a Bruker MD-4 resonator. Temperature control was achieved using an Oxford Instruments CF935 cryogen flow cryostat using liquid helium (5−100 K) or liquid nitrogen (>100−240 K) and a Mercury ITC temperature controller. For $Cu(acac)_2$ inversion recovery experiments below 100 K, a Bruker MS-5 resonator and a ER 4118HV-CF5-L Flexline Cryogen-Free VT cryostat (ColdEdge) was used. 294 K data were collected without temperature control, and the ambient temperature of the room was recorded. Echo-detected field sweep spectra employed a two-pulse Hahn-echo sequence ($\pi/2-\tau-\pi-\tau-$echo). Inversion recovery experiments employed the pulse sequence $\pi-t-\pi/2-\tau-\pi-\tau-$echo, where t is the variable time delay and τ is a fixed constant. Unless otherwise specified, picket fence saturation recovery experiments employed sixteen consecutive $\pi/2$ pulses with a fixed interpulse delay of 1 μs, followed by a Hahn-echo

detection sequence after a recovery time t. The fixed delay τ was optimized independently for each sample to maximize the echo intensity. The video gain was optimized at each field position and temperature. Four-step phase cycling was used for inversion recovery and saturation recovery measurements to eliminate influence of secondary echoes and microwave ringdown, while two-step phase cycling was used for Hahn-echo decay measurements. Unless otherwise noted, the π/2 pulses had a duration of 8 ns at X-band, while the π pulses had a duration of 16 ns. The shot repetition time (SRT) was always set to at least 5 times the value of T_1 to eliminate effects of incomplete recovery, though in most cases SRT was set much longer. Visual inspection of the inversion recovery trace was conducted in each instance to ensure sufficient recovery time was permitted. Inversion recovery and saturation recovery experiments were fit to stretched exponential functions in Matlab R2023b according to Equation S1. Hahn-echo decay experiments to quantify T_m were fit to Equation S2, which yielded an improved uncertainty for T_m in the presence of ESEEM. Error bars were obtained from the 95% confidence intervals on the fitted T_1 or T_m parameters.

$$I = Ae^{\left[-\left(\frac{t}{T_1}\right)^{\beta}\right]} + I_0 \quad \textbf{(S1)}$$

$$I = Ae^{\left[-\frac{2\tau}{T_m}\right]} + I_0 \quad \textbf{(S2)}$$

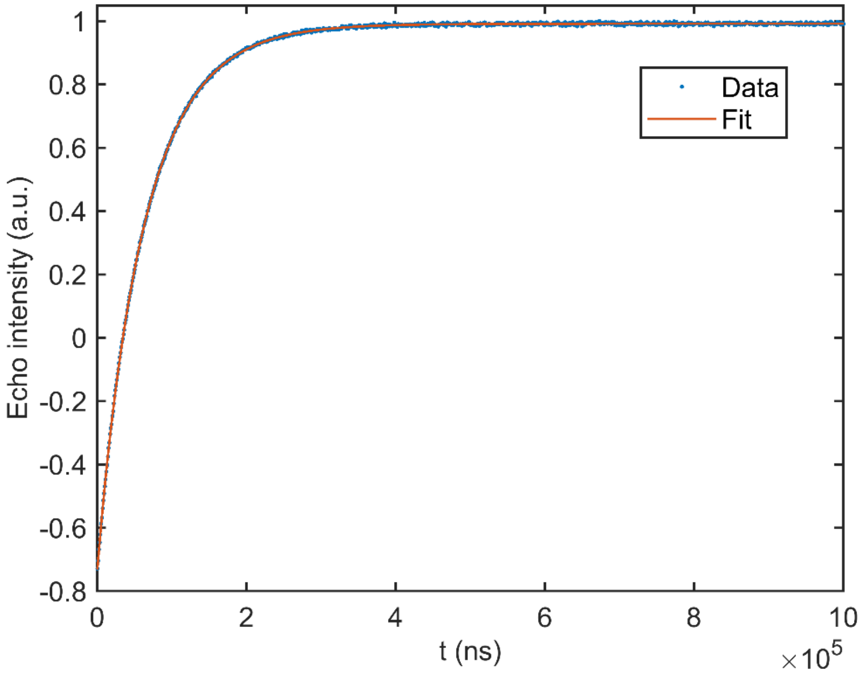

Figure S1: Example inversion recovery and stretched exponential fit for polycrystalline 0.1% $Cu(acac)_2$ in $Pd(acac)_2$ at 3282 G, 25 K, ν = 9.418 GHz.

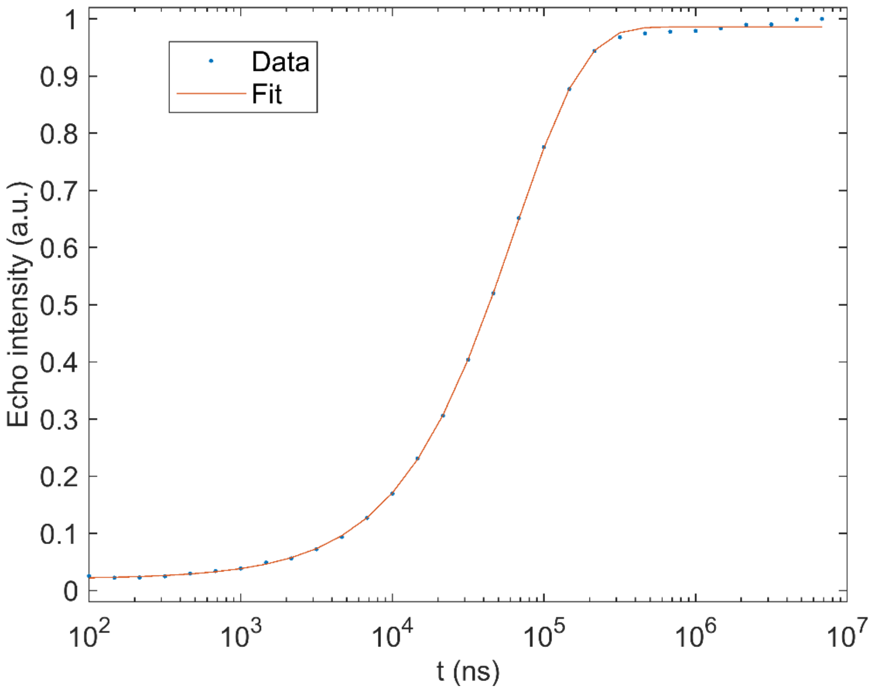

Figure S2: Example saturation recovery and stretched exponential fit for polycrystalline 0.1% $Cu(acac)_2$ in $Pd(acac)_2$ at 3282 G, 25 K, ν = 9.418 GHz.

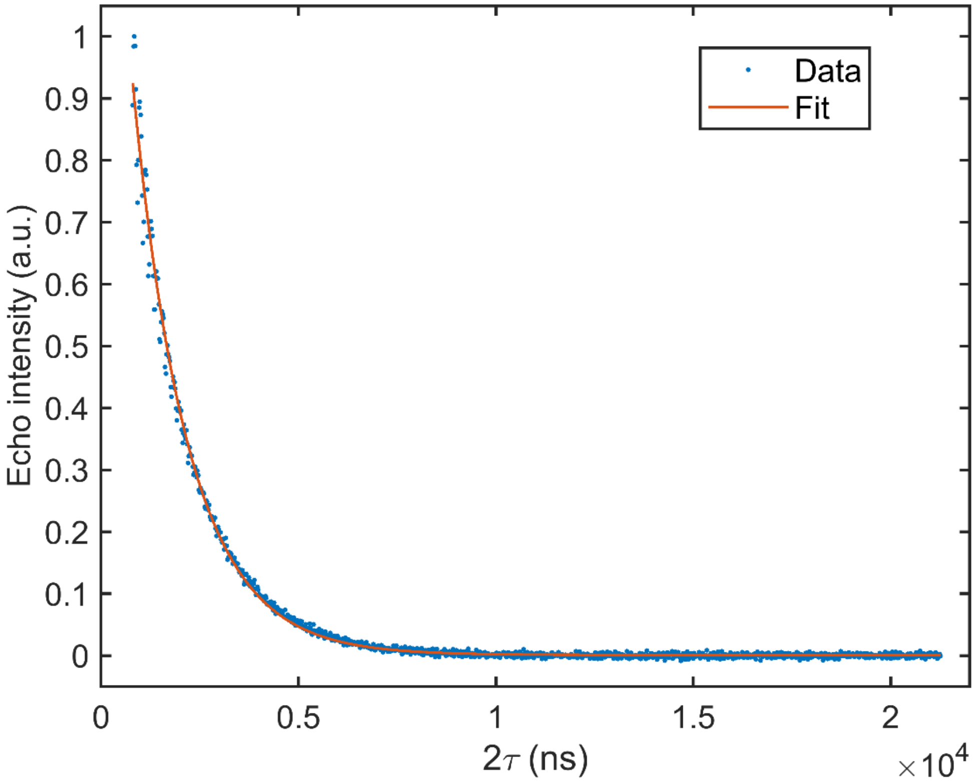

Figure S3: Example Hahn-echo decay and monoexponential fit for polycrystalline 0.1% $Cu(acac)_2$ in $Pd(acac)_2$ at 3282 G, 25 K, ν = 9.418 GHz.

3. Temperature-dependence of T_1 and T_m for Cu(acac)$_2$

Temperature-dependent T_1 data were collected for Cu(acac)$_2$ via inversion recovery and 2-pulse Hahn-echo decay. Relative uncertainties are derived from the 95% confidence intervals based on standard errors of the nonlinear least squares fit. Measurements from 6.5 – 100 K were conducted at a microwave frequency of 9.4183 GHz using a Bruker MS-5 resonator, while measurements from 100 – 155 K were conducted at 9.7266 GHz using a Bruker MD-4 resonator. Field positions were adjusted accordingly to maintain a constant position on the EDFS spectrum.

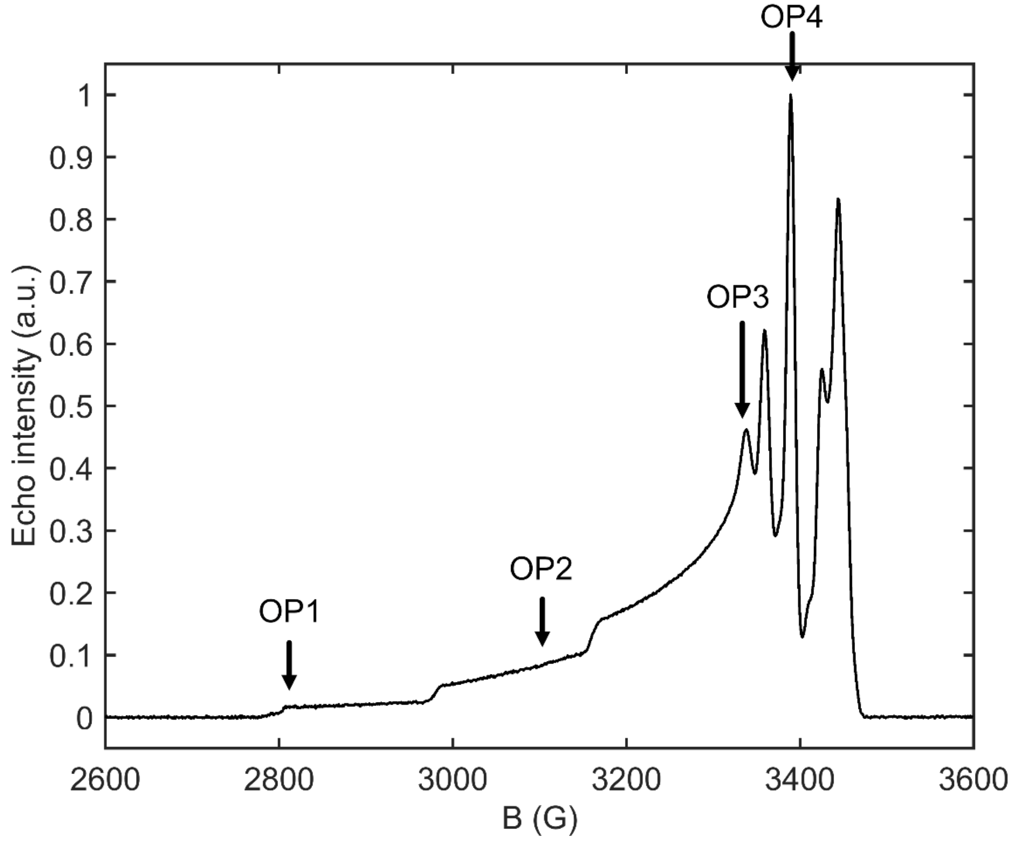

Figure S4. 100 K echo-detected field sweep of 0.1% Cu(acac)$_2$ in Pd(acac)$_2$ at ν = 9.727 GHz. Observer positions for temperature-dependent T_1 / T_m indicated.

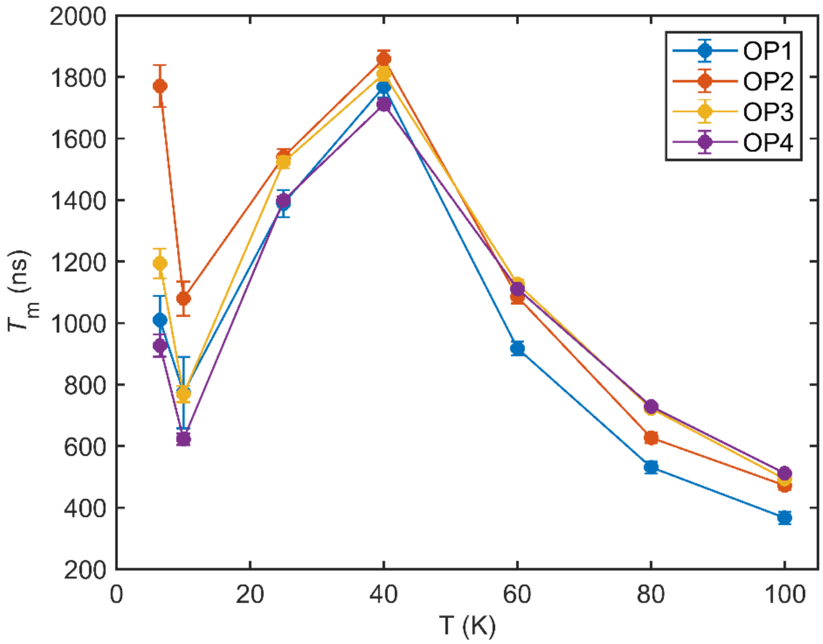

Figure S5: Temperature-dependent T_m by Hahn-echo decay for 0.1% Cu(acac)$_2$ in Pd(acac)$_2$.

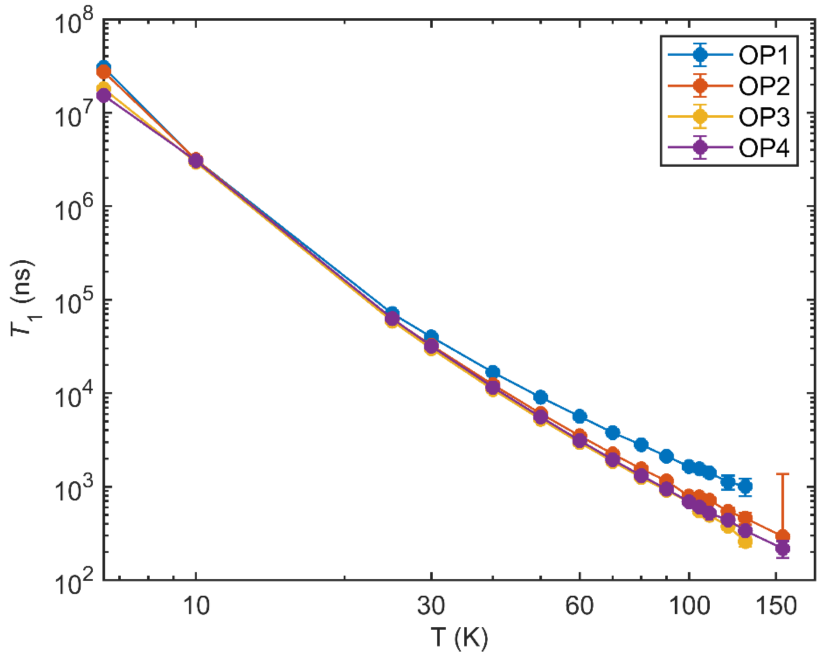

Figure S6: Temperature-dependent T_1 by inversion recovery for 0.1% Cu(acac)$_2$ in Pd(acac)$_2$.

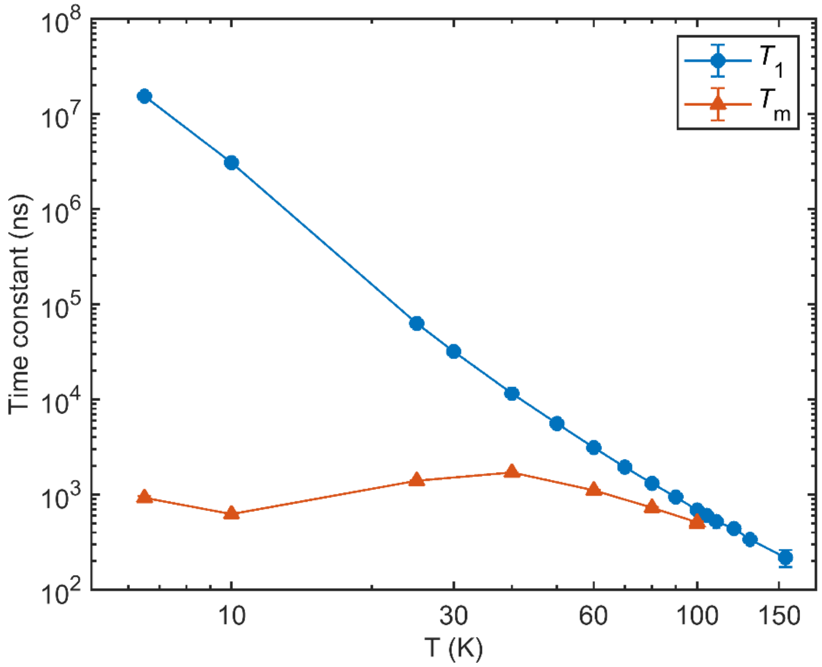

Figure S7: Comparison of T_1 by inversion recovery and T_m for 0.1% $Cu(acac)_2$ in $Pd(acac)_2$ at OP4 (powder line).

Table S1: Temperature-dependent T_m data for 0.1% $Cu(acac)_2$ in $Pd(acac)_2$

T (K)	T_m (ns)				% uncertainty			
	OP1 (2715 G)	OP2 (3050 G)	OP3 (3228 G)	OP4 (3282 G)	OP1 (2715 G)	OP2 (3050 G)	OP3 (3228 G)	OP4 (3282 G)
6.5	1010	1771	1194	927	7.80	3.87	4.01	3.97
10	774	1080	769	623	14.96	5.16	3.41	2.97
25	1389	1541	1523	1399	3.21	1.65	1.24	0.94
40	1768	1858	1810	1711	2.02	1.52	1.18	0.87
60	917	1085	1127	1111	2.45	1.89	1.26	0.86
80	531	627	722	729	3.58	2.66	1.63	1.08
100	367	472	493	512	5.57	2.93	1.61	0.98

Table S2: Temperature-dependent T_1 data by inversion recovery for 0.1% $Cu(acac)_2$ in $Pd(acac)_2$

T (K)	T_m (ns)				% uncertainty			
	OP1 (2715 G / 2809 G)	OP2 (3050 G / 3151 G)	OP3 (3228 G / 3338 G)	OP4 (3282 G / 3389 G)	OP1	OP2	OP3	OP4
6.5	3.061E+07	2.736E+07	1.804E+07	1.534E+07	2.09	1.80	1.22	1.10
10	3.142E+06	3.146E+06	2.950E+06	3.076E+06	6.93	2.81	1.53	1.13
25	7.094E+04	6.216E+04	5.903E+04	6.286E+04	2.64	0.72	0.29	0.21
30	3.997E+04	3.258E+04	2.997E+04	3.187E+04	1.46	0.45	0.17	0.13
40	1.673E+04	1.232E+04	1.094E+04	1.160E+04	0.74	0.49	0.18	0.12
50	9.068E+03	6.080E+03	5.263E+03	5.568E+03	1.44	0.50	0.17	0.10

60	5.654E+03	3.503E+03	2.959E+03	3.124E+03	2.50	1.00	0.30	0.16
70	3.791E+03	2.244E+03	1.861E+03	1.941E+03	3.98	1.55	0.51	0.29
80	2.819E+03	1.552E+03	1.252E+03	1.310E+03	2.85	2.82	0.86	0.48
90	2.114E+03	1.155E+03	9.157E+02	9.423E+02	3.27	2.96	0.91	0.41
100	1.647E+03	7.904E+02	6.855E+02	6.896E+02	5.02	6.44	1.82	0.69
105	1.551E+03	7.750E+02	5.463E+02	6.042E+02	6.94	4.11	2.75	0.90
110	1.408E+03	7.132E+02	4.958E+02	5.223E+02	7.83	4.67	3.21	1.14
120	1.124E+03	5.459E+02	3.766E+02	4.413E+02	17.61	9.15	5.56	1.71
130	1.007E+03	4.594E+02	2.598E+02	3.375E+02	21.61	14.20	12.08	3.39
155	5.118E+02	2.938E+02	9.653E+01	2.184E+02	1841.53	368.14	4736.92	20.35
177		2.006E+02	9.756E+01	2.705E+02		105.94	110.67	50.53

Table S3: Temperature-dependent stretching factors β for inversion recovery for 0.1% $Cu(acac)_2$ in $Pd(acac)_2$

T (K)	Stretching factor			
	OP1	OP2	OP3	OP4
6.5	0.751	0.679	0.649	0.666
10	0.822	0.796	0.785	0.827
25	0.911	0.924	0.937	0.968
30	0.948	0.946	0.956	0.971
40	0.950	0.947	0.962	0.967
50	0.955	0.950	0.964	0.969
60	0.948	0.952	0.961	0.961
70	0.936	0.946	0.956	0.949
80	0.976	0.951	0.952	0.943
90	0.966	0.977	0.968	0.953
100	0.973	0.902	0.967	0.942
105	0.979	0.959	0.836	0.929
110	0.973	0.983	0.844	0.918
120	0.950	0.963	0.820	0.951
130	1.100	0.998	0.783	0.937
155		1.008		0.952

4. Comparison of T_1 temperature dependence for differing pulse sequences

In this work, T_1 measurements for $Cu(acac)_2$ were collected by both inversion recovery and saturation recovery. Measurements for CuOEP were here acquired by saturation recovery, and inversion recovery results have been previously published,[2] enabling comparison of the behavior between the two pulse sequences. In the high temperature limit, saturation and inversion recovery are found to give extremely similar rates (**Figure S8**). Below 30 K, inversion recovery measures a significantly faster echo relaxation rate, indicating the enhanced role of spectral diffusion in the inversion recovery time constant. We note that the magnitude of the deviation is similar for both compounds, and the ordering does not alter for the two pulse sequences. Inversion recovery is therefore unlikely to perturb compound-to-compound comparisons. It does, however, significantly reduce the log-log slope at low temperatures (**Figure S9**). We therefore employ saturation recovery in the main text when comparing the log-log slope behavior to VTVH T_1 anisotropy.

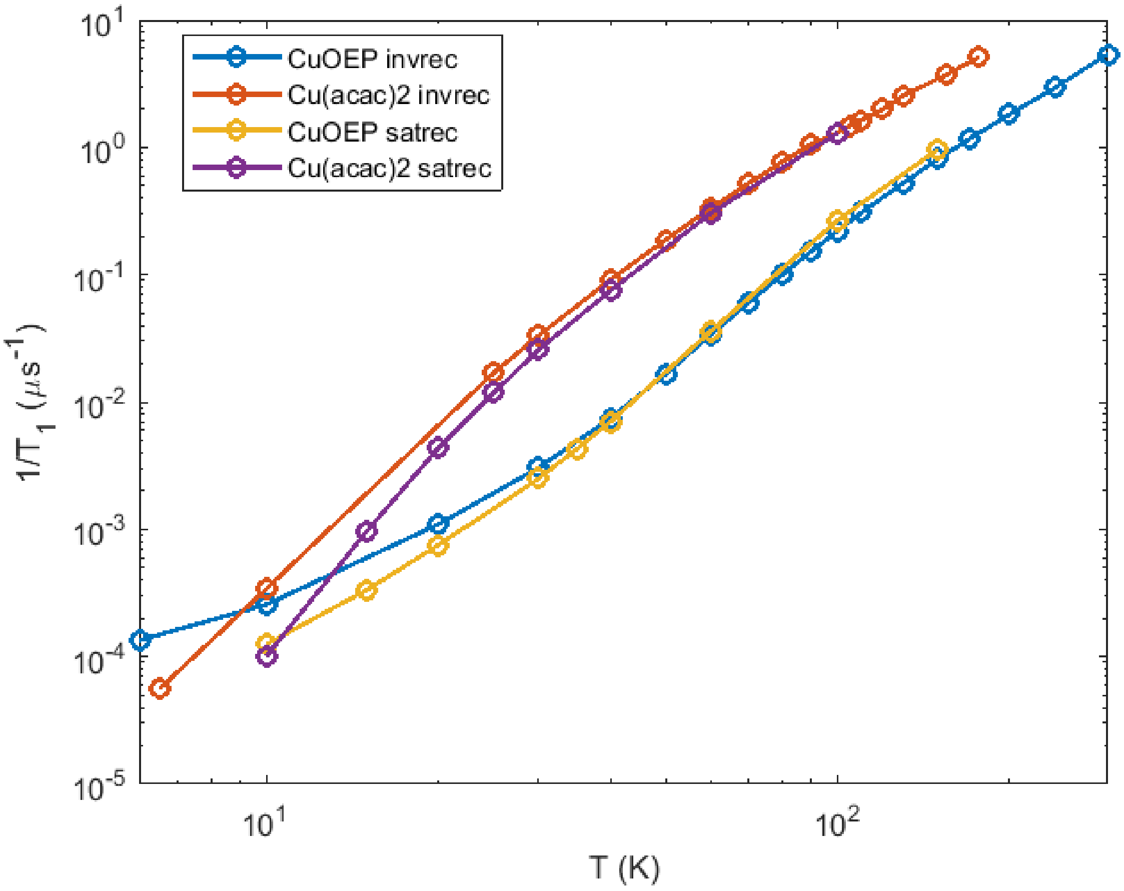

Figure S8: Comparison of T_1 measured by inversion and saturation recovery for 0.1% $Cu(acac)_2$ in $Pd(acac)_2$ and 1% CuOEP in ZnOEP polycrystalline powders. Data taken near pure perpendicular position (3315 G for $Cu(acac)_2$ at 9.7060 GHz, 3369 G for CuOEP at 9.6291 GHz).

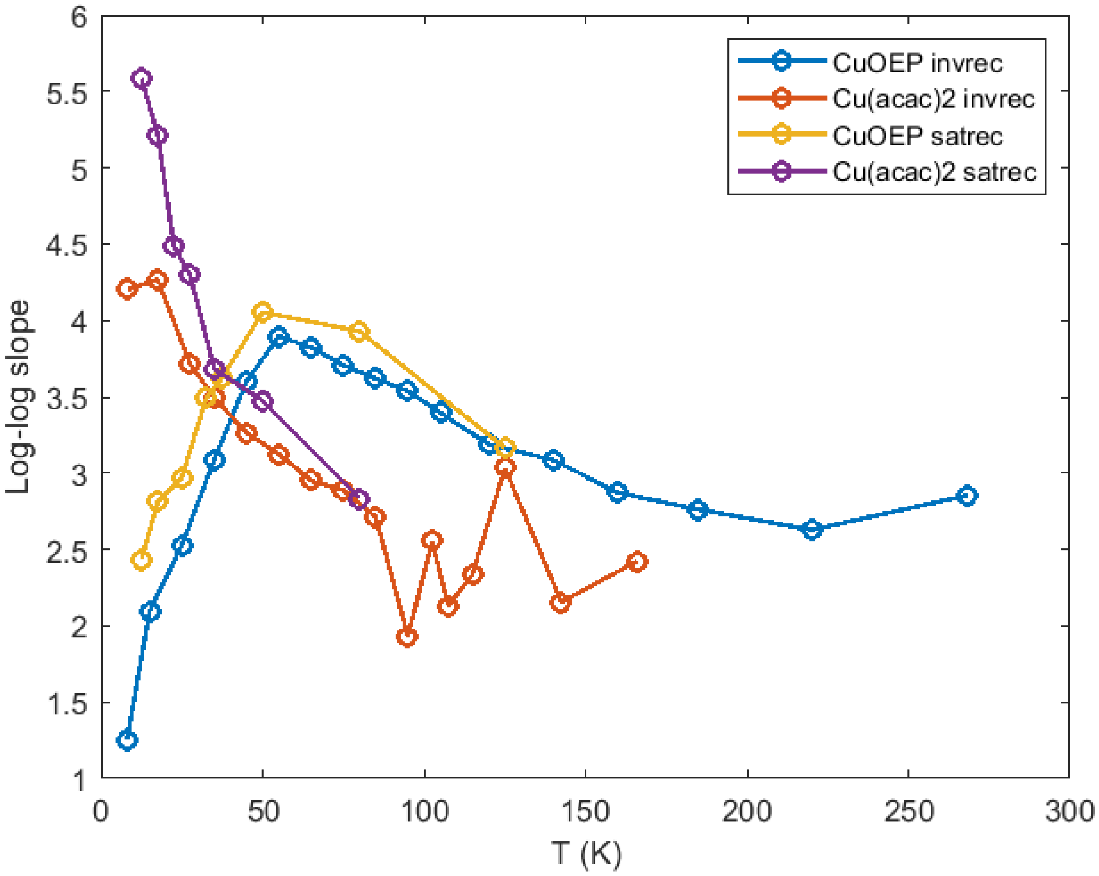

Figure S9: Comparison of the slope of T_1 measured by inversion and saturation recovery for 0.1% $Cu(acac)_2$ in $Pd(acac)_2$ and 1% CuOEP in ZnOEP polycrystalline powders. Log-log slope is defined as the slope of a graph of $\log_{10}(1/T_1)$ vs. $\log_{10}$(T), and indicates the power law scaling of the data.

5. T_1 local mode fitting

Local mode fitting was conducted according to a previously reported method.[2] Fits for CuOEP with T_1 data collected by inversion recovery were previously reported,[2] with E_{loc} = 258 cm^{-1} determined. In the present work, VTVH-T_1 data were collected by the saturation recovery pulse sequence instead. The temperature-dependent rates at the purest perpendicular (fastest-relaxing) field position were extracted and fit (**Figure S10**). The saturation recovery fit yields E_{loc} = 259.8 cm^{-1}, in excellent agreement with the previous determination.

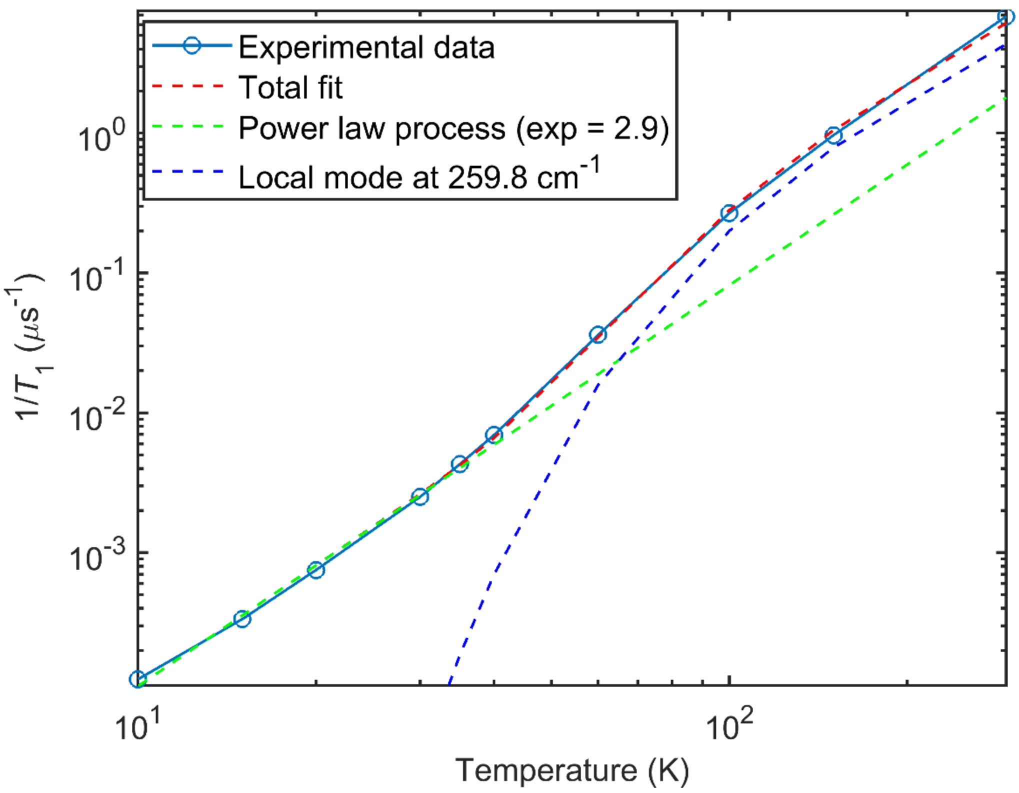

Figure S10: Local mode fitting of temperature-dependent T_1 by saturation recovery for 1:100 CuOEP:ZnOEP powder, collected at the perpendicular position.

However, a satisfactory local mode fit for $Cu(acac)_2$ could not be found. First, a least-squares fit was conducted in an identical manner to the CuOEP data (**Figure S11**). Despite a good mathematical fit, the result is physically unrealistic. The power law process, which is supposed to capture the role of the direct process relaxation and/or Raman relaxation mediated by low-energy acoustic phonons, reemerges to dominate the spin relaxation rate at high temperatures. This unphysical fit arises because the log-log slope of the T_1 vs. T data is steep at low temperatures, while it is shallow for CuOEP. As a result, it is not possible to find a power law function that fits only the low temperature data without grossly overpredicted the relaxation rate at high T.

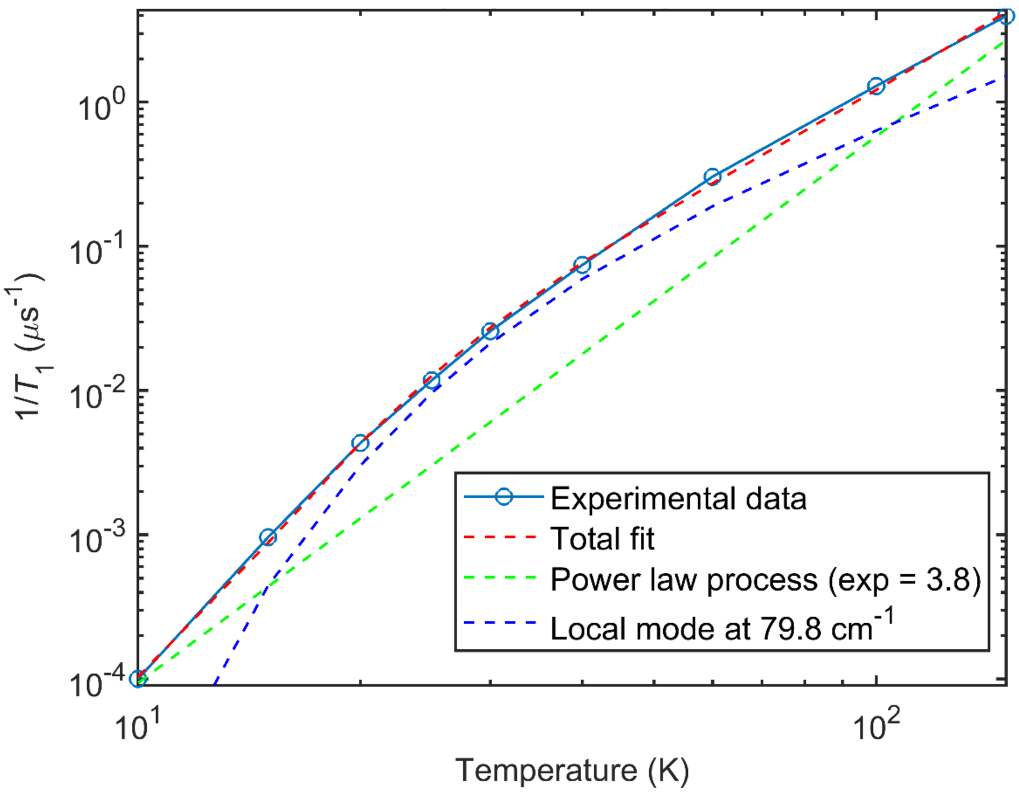

Figure S11: Local mode fitting of temperature-dependent T_1 by saturation recovery for 1:1000 $Cu(acac)_2$:$Pd(acac)_2$ powder, collected at the perpendicular position.

Several attempts were made to manually fit the T_1 vs. T data with a single local mode form (**Figures S12-S14**). No matter the energy chosen for the local mode, some of the T_1 vs. T curve cannot be fit. Thus, the VTVH-T_1 anisotropy methodology can more effectively delineate regimes of spin relaxation in $Cu(acac)_2$ than local mode fitting can.

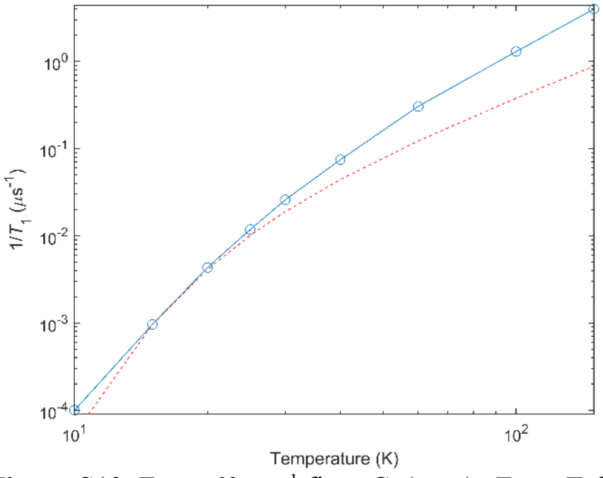

Figure S12: E_{loc} = 60 cm^{-1} fit to $Cu(acac)_2$ T_1 vs. T data.

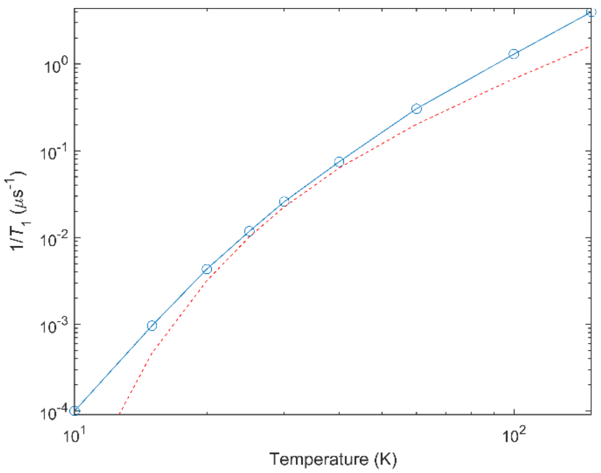

Figure S13: E_{loc} = 80 cm^{-1} fit to Cu(acac)$_2$ T_1 vs. T data.

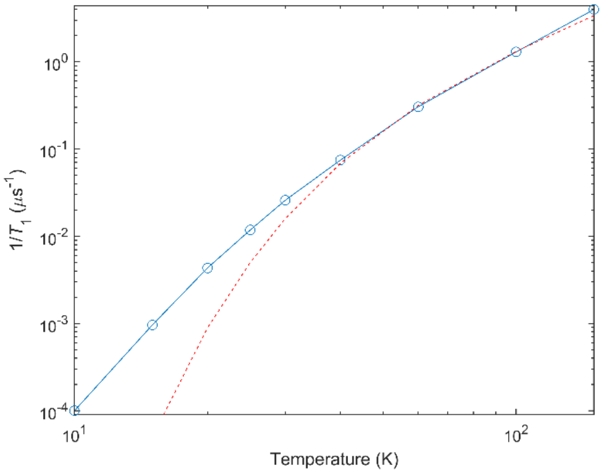

Figure S14: E_{loc} = 120 cm^{-1} fit to Cu(acac)$_2$ T_1 vs. T data.

6. Influence of saturation recovery pulse sequence parameters on powder T_1 anisotropy

The low-temperature T_1 anisotropy shapes for Cu(acac)$_2$ and CuOEP cannot be fit by simple geometric functions such as $\sin^2\theta$. This raises the question of whether they constitute true anisotropy in the spin relaxation rate, or instead, anisotropy in residual spectral diffusion that is not successfully eliminated by the picket fence saturation recovery pulse sequence. To probe this, T_1 anisotropy traces for Cu(acac)$_2$ were acquired at 20 K under five different parameter sets for the saturation recovery pulse sequence. These parameter changes include varying the length of the pulses in the sequence (π = 32 ns and $\pi/2$ = 16 ns, vs. π = 16 ns and $\pi/2$ = 8 ns), varying the number of $\pi/2$ pulses in the picket fence (8 vs. 16), and varying the interpulse spacing Δ in the picket fence (100, 400, or 1000 ns).

As shown in **Figure S15**, small changes in the isotropic magnitude of $1/T_1$ are observed, on the order of 5%, which falls within the typical limit of consistency for pulse EPR relaxation experiments among different pulse sequences and concentrations.[2] Smaller values of $1/T_1$ indicate more effective suppression of the spectral diffusion contribution to echo intensity loss. The biggest difference revealed is that the π = 32 ns pulse sequence less effectively suppresses spectral diffusion than the π = 16 ns, as expected due to the narrower polarization hole in frequency space generated by the more selective 32 ns pulse. 5% changes in the isotropic spin relaxation rate, however, are not consequential for the analysis.

Most importantly, these alterations to the saturation recovery pulse sequence do not change the shape of the measured T_1 anisotropy. If the 20 K Cu(acac)$_2$ anisotropy shape were due to incomplete spectral diffusion removal by the picket fence saturation recovery pulse sequence, then altering the parameters of that pulse sequence should enhance or diminish the amount of spectral diffusion removed and, thus, change the observed anisotropy. Because this is not the case, we conclude that the low-temperature powder T_1 anisotropy shapes are most likely caused by true spin relaxation anisotropy rather than spectral diffusion.

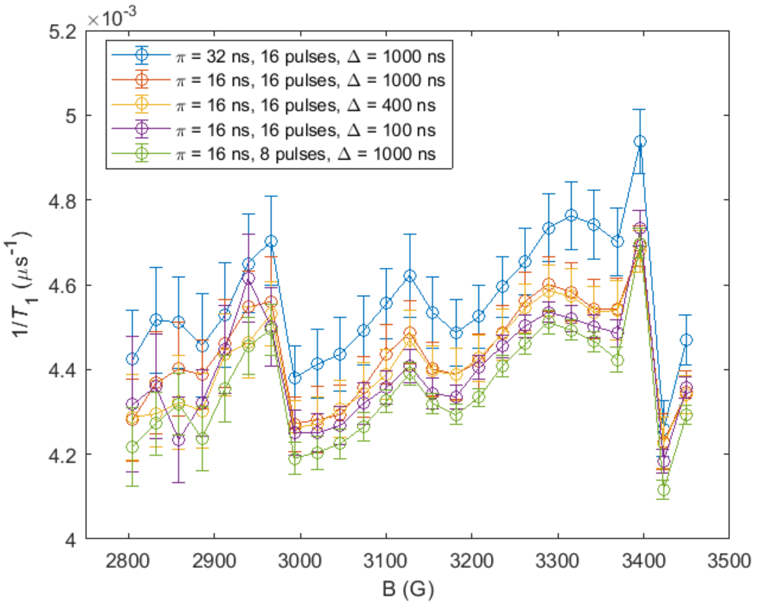

Figure S15: Influence of saturation recovery pulse sequence parameters on T_1 anisotropy for 1:1000 Cu(acac)$_2$:Pd(acac)$_2$ at 20 K.

7. Influence of paramagnetic concentration on powder T_1 anisotropy

To probe the mechanistic origin of the low-temperature T_1 anisotropy shape, 20 K saturation recovery measurements were conducted for varying paramagnetic concentrations of $Cu(acac)_2$ doped into $Pd(acac)_2$. Doping levels of 1:100, 1:300, 1:3000, and 1:10000 Cu:Pd were measured in sequence at 20 K under identical cryostat equilibration conditions. The T_1 anisotropy for 1:100 is substantially different from all other samples (**Figure S16A**), while the T_1 anisotropy for 1:300 deviates modestly from those of 1:3000 and 1:10000 (**Figure S16B**). The 1:100 EDFS displays significantly broadened features due to electron spin dipole-dipole interactions; moderate broadening is observed in the 1:300 EDFS, while the 1:3000 and 1:10000 EDFS spectra are nearly identical (**Figure S16C-D**). Thus, the concentration changes in the observed T_1 anisotropy correlate directly to the concentration changes in the EDFS line shape. When comparing the 1:3000 T_1 anisotropy to the 1:1000 T_1 anisotropy used in the analysis for **Figure 2** in the main text, an offset of ~5% is observed, likely due to variations in cryostat equilibration on the different days in which these measurements were taken (**Figure S16E**). However, the mean normalized 1:3000 and 1:1000 traces display identical T_1 anisotropy within the uncertainty of the measurement (**Figure S16F**).

These data indicate that the 1:1000 $Cu(acac)_2$:$Pd(acac)_2$ samples used in the main text are satisfactorily representative of the paramagnetically dilute limit. Thus, the new T_1 anisotropy pattern arising at low temperature (<25 K) at 1:1000 concentration is a fundamental property of the isolated paramagnetic molecule. It is unlikely that the low temperature T_1 anisotropy arises from relaxation mechanisms involving simultaneous flips of two electron spins. Explanations on the basis of phonons/vibrations are most consistent with the data.

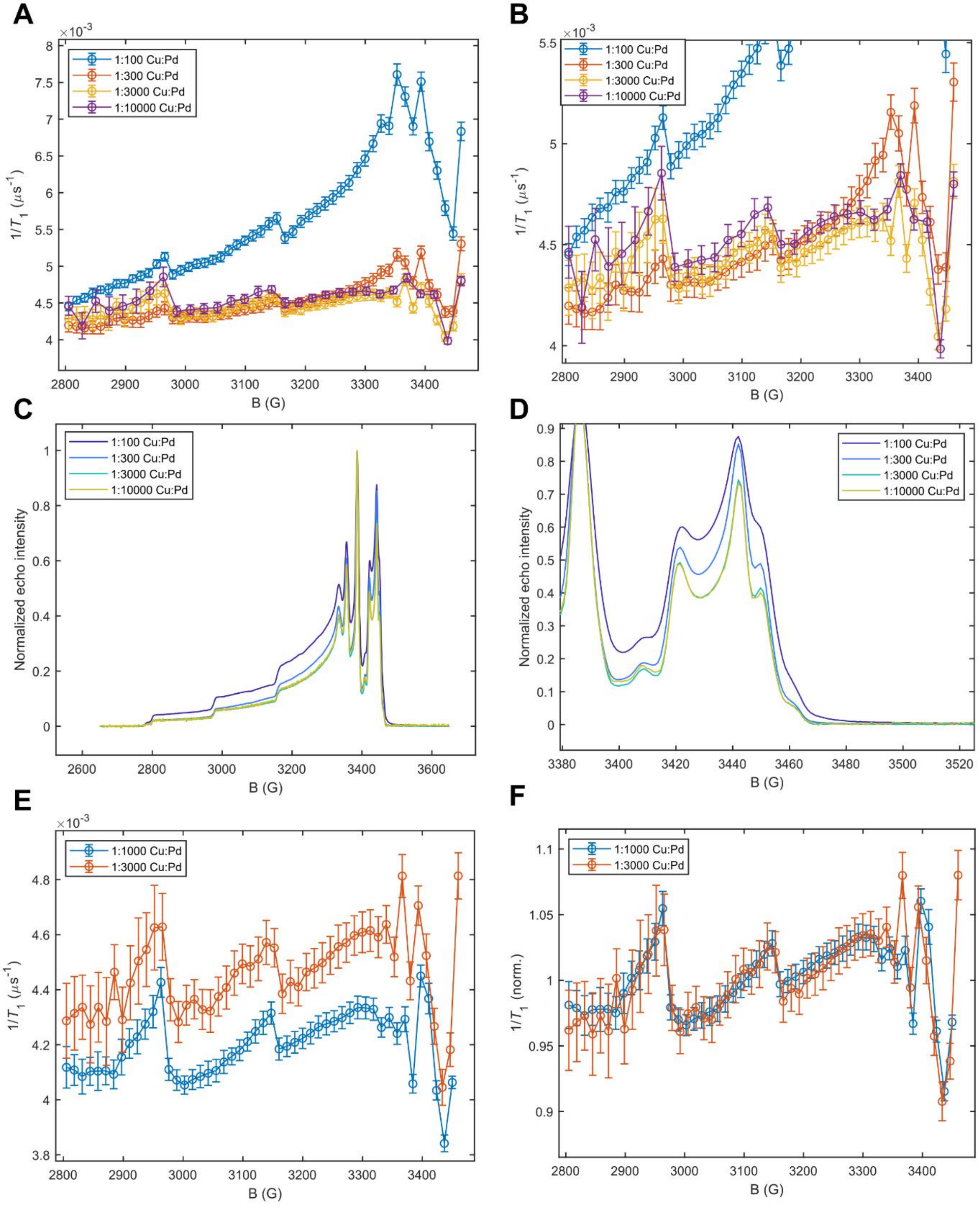

Figure S16: Impact of paramagnetic concentration on 20 K T_1 anisotropy for $Cu(acac)_2$ in $Pd(acac)_2$. (A) T_1 anisotropy for four concentrations measured on the same day. (B) Zoomed-in version of panel A. (C) 20 K echo-detected field sweep (EDFS) spectra of samples in panel A, normalized to the maximum intensity. (D) Zoomed-in version of panel C. (E) T_1 anisotropy comparison to the 1:1000 data used in **Figure 2** of the main text (measured on different days). (F) Mean-normalized comparison between data in panel E.

8. VTVH-T_1 powder anisotropy factor analysis

The high-temperature T_1 anisotropy in Cu(II) and VO(IV) molecular qubits is known to follow an average $\sin^2\theta$ functional form, which can be explicitly computed from the spin Hamiltonian and used to fit the experimental data through least squares regression.[1] For $S = 1$ Cr(*o*-tolyl)$_4$, an anisotropy functional form of average $\sin^2(2\theta)$ was detected.[4] However, in the present work, no simple parametric function of θ was found that could successfully explain the low-temperature T_1 anisotropy patterns. This necessitated a more flexible data analysis procedure than least-squares regression.[5]

Analysis of the T_1 anisotropy for the powder polycrystalline samples was conducted according to a factor analysis procedure, which decomposes the anisotropy data into a sum of additive components (**Figure S17**).[6] Each factor possesses a characteristic spectral shape (the T_1 anisotropy pattern) and a profile of how it contributes to the total anisotropy as a function of temperature, thereby constituting a bilinear matrix factorization of the VTVH-T_1 data matrix (**Figure S18**).

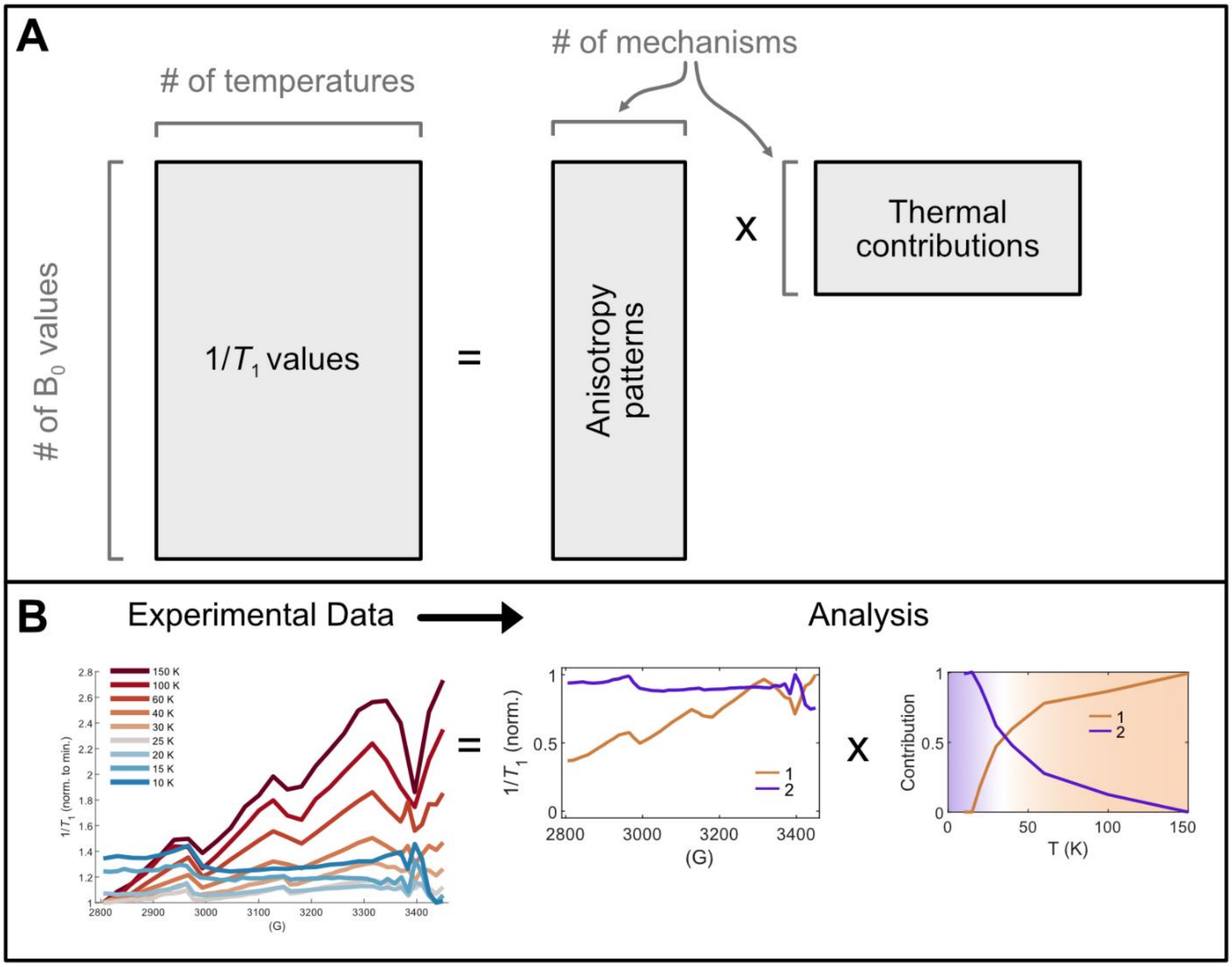

Figure S17: Schematic of the VTVH-T_1 factor analysis procedure. (A) Diagram of the matrix factorization of the $1/T_1$ values into fundamental anisotropy patterns and associated thermal contributions. (B) Illustration of how the Cu(acac)$_2$ plots in **Figure 2A, 2C-D** correspond to this matrix factorization schematic. Note that the $1/T_1$ data are scaled and normalized before applying the factorization techniques, as described below. Note that the leftmost panel can also be represented as a 3D surface with two independent axes: B_0 (G) and T (K).

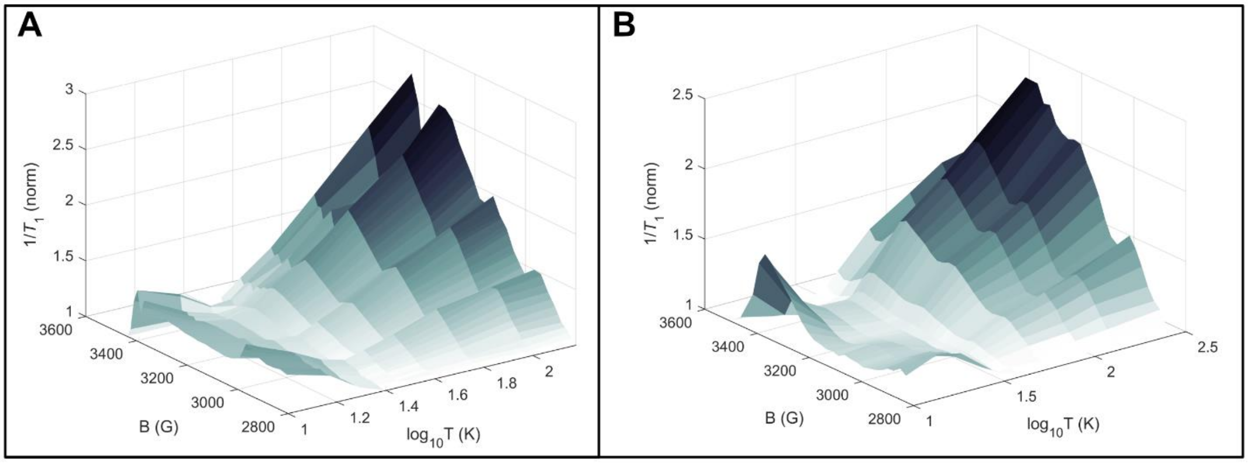

Figure S18: Surface plot representation of the VTVH-T_1 datasets from **Figure 2A-B**, emphasizing the 2D matrix nature of the acquired relaxation data. (A) $Cu(acac)_2$ powder sample VTVH-T_1. (B) CuOEP powder sample VTVH-T_1.

The spectral shapes and contributions need not be known *a priori*; rather, they are obtained from the data subject to physical constraints. If no constraints are employed, an infinite number of factor decompositions exist with equal goodness of fit. If constraints are employed, either a family of solutions or a single unique solution may be obtained.[7] Our factor analysis procedure employed constraints of non-negativity on both spectral shapes and contributions, as well as normalization to a height of one for all spectral shapes. For the datasets analyzed herein, these constraints were sufficient to produce a uniquely determined factor decomposition, so an analysis of factor ambiguity is not required. The factor analysis technique used is also referred to as non-negative matrix factorization (NMF),[8] soft modeling,[9] or multivariate curve resolution – alternating least squares (MCR-ALS)[10] in various branches of scientific literature.

A two-step procedure was employed to obtain the T_1 anisotropy factors, which yields two sets of factors referred to as the "shapes" and "mechanisms" (Matlab code provided in **Supporting Information Section 15**). First, the anisotropic component of the spin relaxation was isolated and normalized by computing $D/max(D) - min(D/max(D))$ for the T_1 anisotropy spectrum D at each temperature (see **Figure S19**). Then, singular value decomposition (SVD) was employed to ascertain the appropriate number of factors to be fit (**Figures S20-S24**). For $Cu(acac)_2$, two factors were found to give a satisfactory fit to the temperature-dependent T_1 anisotropy. For CuOEP, three factors were required, owing to the prominent growth of a peak at ~3360 G at temperature below 20 K. The best matrix factorization subject to the aforementioned non-negativity and normalization constraints was then obtained through the ALS technique. We refer to this factorization as the "shapes" analysis. This representation shows how fundamental T_1 anisotropy shapes (and additionally the isotropic shape[4]) change in prominence as the temperature changes. It does not make direct claims about the spin relaxation induced *by a particular vibrational mode*, however, as any given vibrational mode may induce a mixture of isotropic and anisotropic spin relaxation in accordance with the nature of the molecule's electronic structure.

To draw correspondence between T_1 anisotropy and the contributions of specific vibrational modes, the second stage analysis ("mechanisms") is conducted. Given a set of k+1 shapes (the k anisotropy shapes plus the single constant, isotropic shape), a linear combination is formed to reduce these to the best k factors describing the original normalized T_1 data (including both the isotropic and anisotropic components of spin relaxation). This linear combination corresponds to dimension reduction of 1 in the factor representation,

and is solved by nonlinear least squares optimization, akin to the technique of resolving factor analysis (RFA).[11] The idea is as follows: since each vibrational mode or mechanism will in general contain both an anisotropic T_1 contribution and an isotropic contribution that is the same at all fields, the isotropic component of spin relaxation should be partitioned between the different vibrational mechanisms along with the anisotropic shapes. Each vibrational mechanism can thus be described by the relative fractions of constituent shapes. This analysis allows statements about which types of vibrational modes dominate spin relaxation at different temperatures. We note that this analysis should be used carefully, as changes in the temperature range fitted can alter the percentage of isotropic vs. anisotropic relaxation placed into each mechanism. This arises because higher temperatures (> 100 K) accentuate the anisotropy of the $\sin^2\theta$ mechanism, as seen for both Cu(acac)$_2$ and CuOEP; if these temperatures are not included, the high-temperature molecular vibration mechanism will obtain a somewhat more isotropic fit. The absolute differences in mechanism crossover point were found to be on the order of 10 K for different choices of data range to fit. Nevertheless, the relative orderings between Cu(acac)$_2$ and CuOEP remain consistent for all choices, and the mechanism analysis gives a more physically plausible picture of mode contributions than does the shapes analysis. The mechanism results are analyzed in the main text.

Figures S19 – S30 detail the factor analysis decomposition for Cu(acac)$_2$. Two factors were considered necessary to reproduce the salient features of the T_1 anisotropy data.

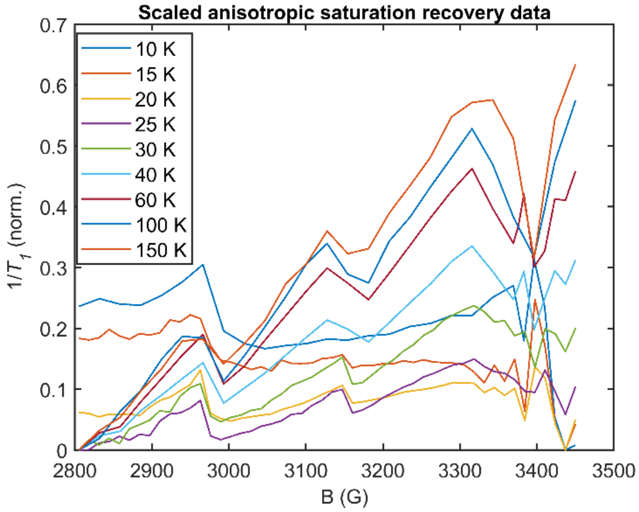

Figure S19: Normalized anisotropic component of the saturation recovery T_1 data for Cu(acac)$_2$ (computed as $D/max(D) - min(D/max(D))$ for each temperature point).

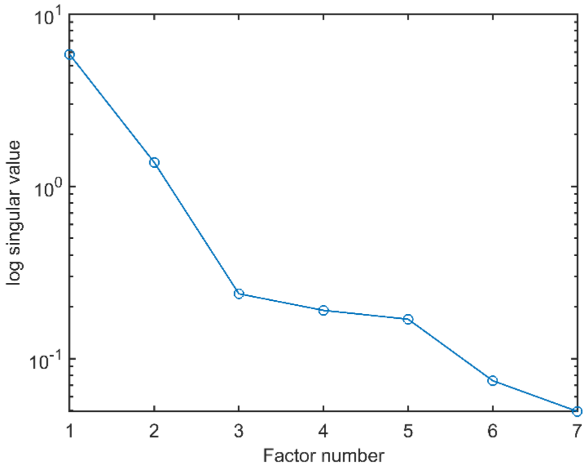

Figure S20: Scree plot of the SVD singular values for $Cu(acac)_2$ T_1 data given in **Figure S19**.

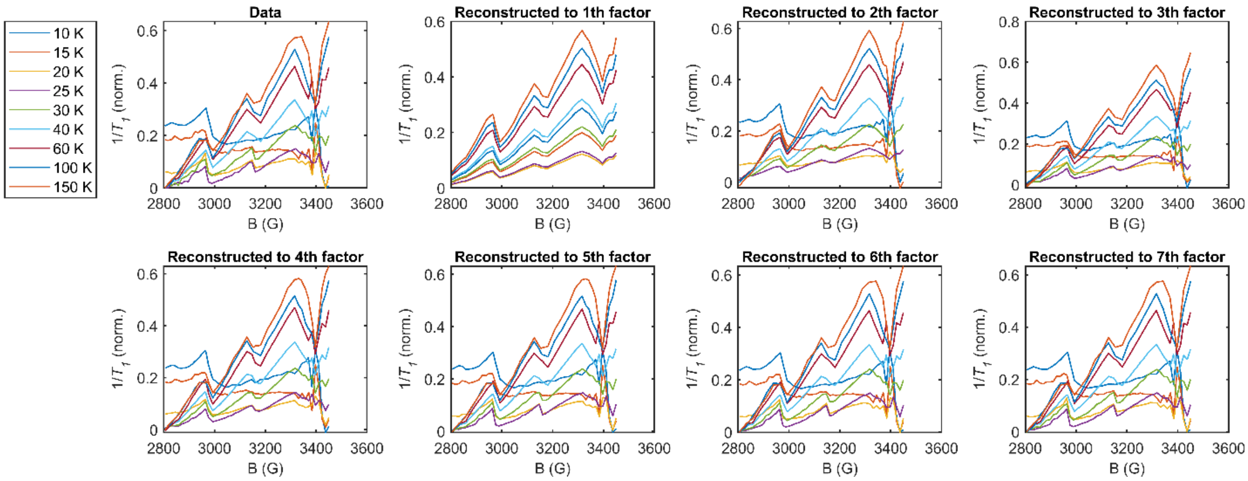

Figure S21: $Cu(acac)_2$ data reconstruction for various rank values used in SVD.

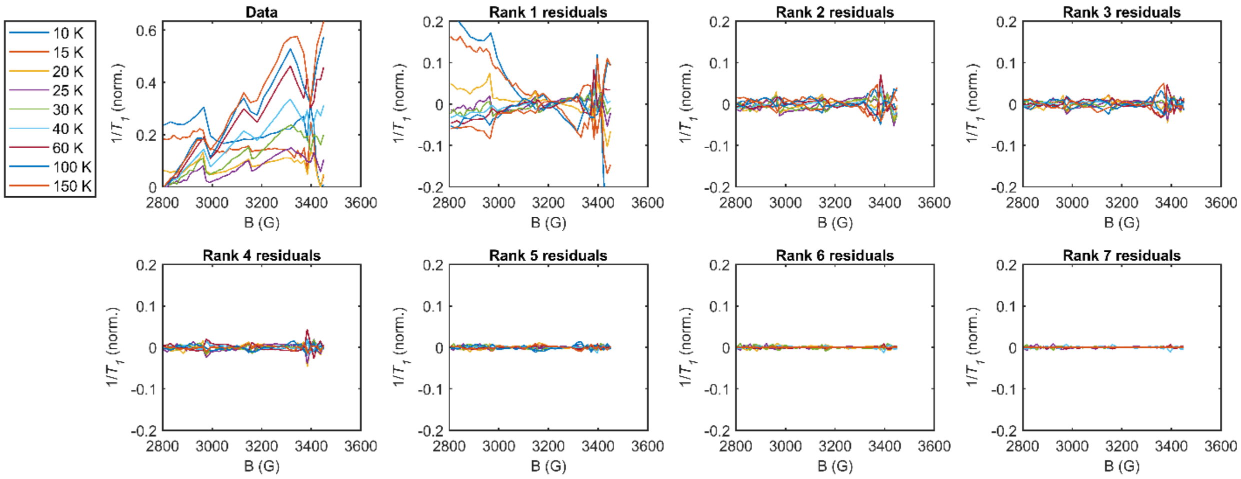

Figure S22: $Cu(acac)_2$ residuals for various rank values used in SVD.

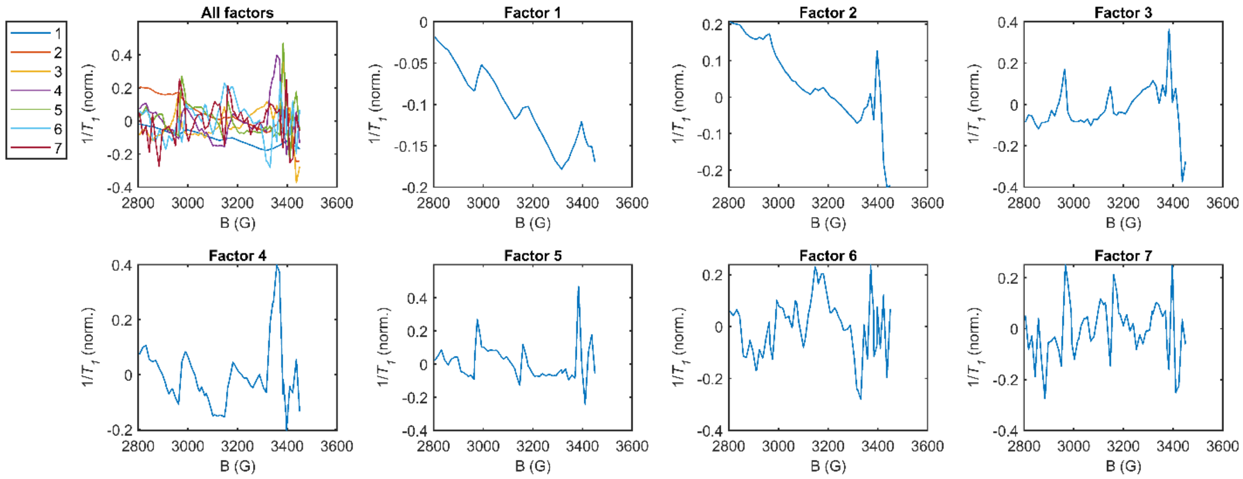

Figure S23: $Cu(acac)_2$ factor spectra for various rank values used in SVD.

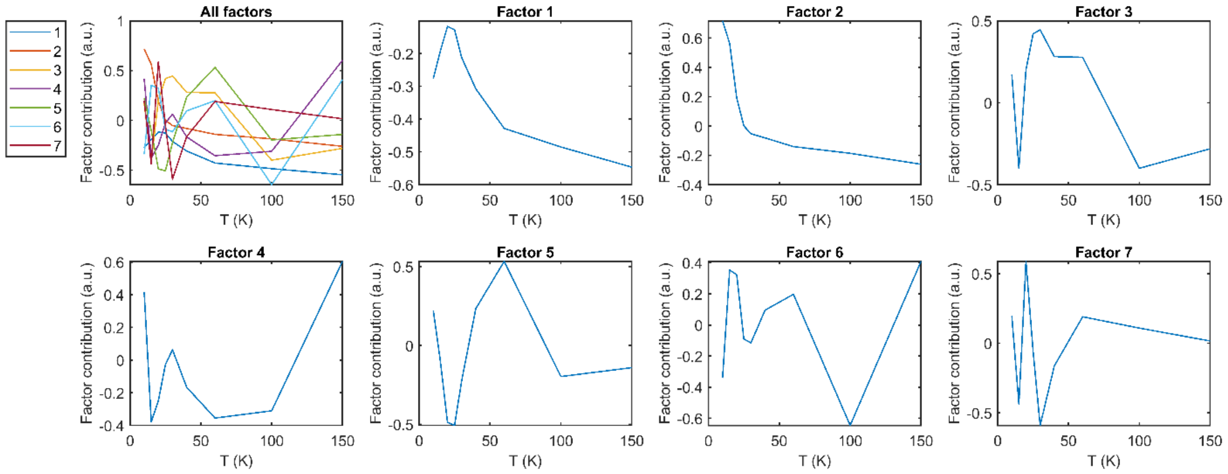

Figure S24: $Cu(acac)_2$ factor contributions for various rank values used in SVD.

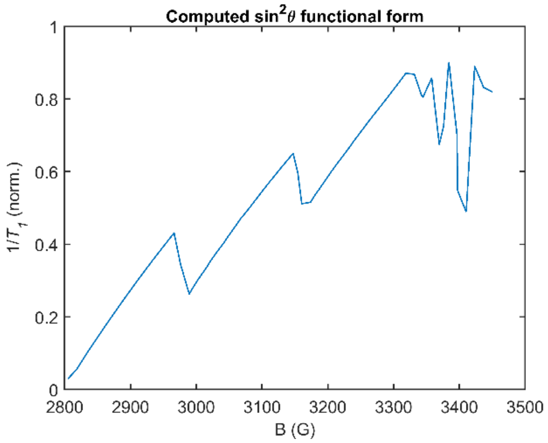

Figure S25: Computed $\sin^2\theta$ functional form for $Cu(acac)_2$.

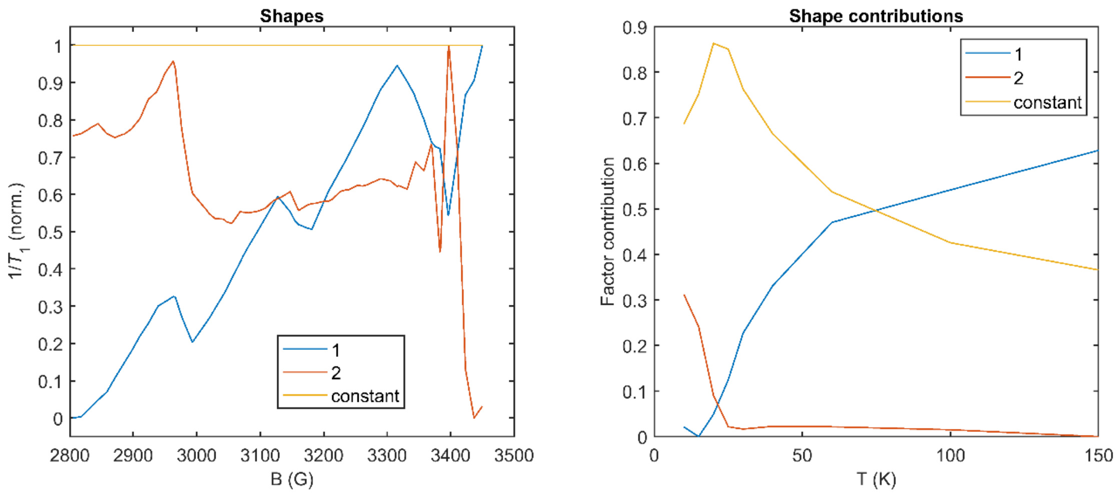

Figure S26: Cu(acac)$_2$ T_1 anisotropy shapes from alternating least-squares factor decomposition.

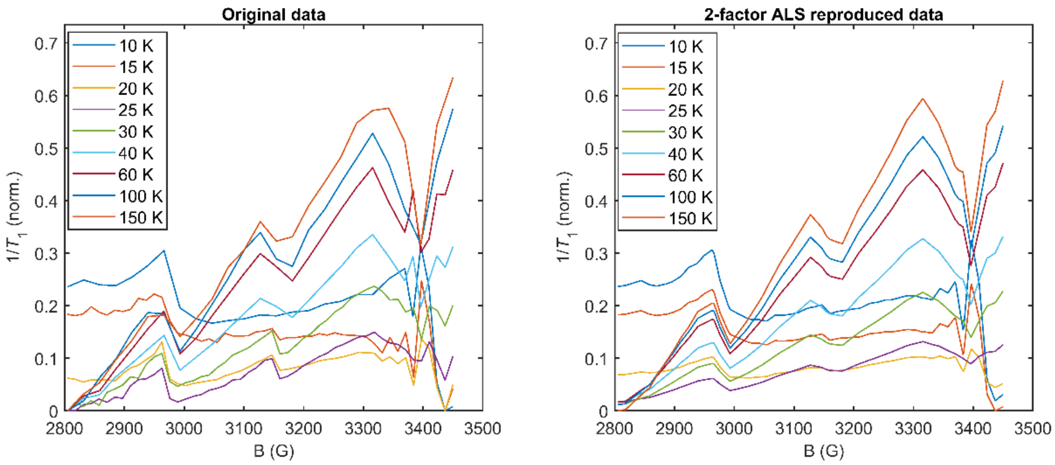

Figure S27: Cu(acac)$_2$ data reproduction from alternating least-squares factor decomposition (T_1 anisotropy shapes).

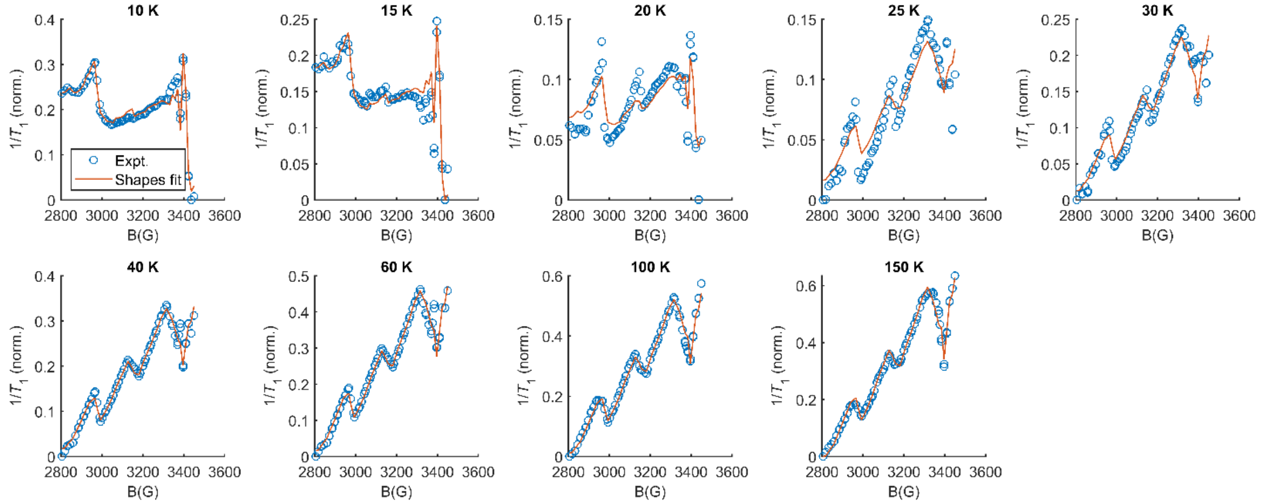

Figure S28: Cu(acac)$_2$ data fit from alternating least-squares factor decomposition (T_1 anisotropy shapes).

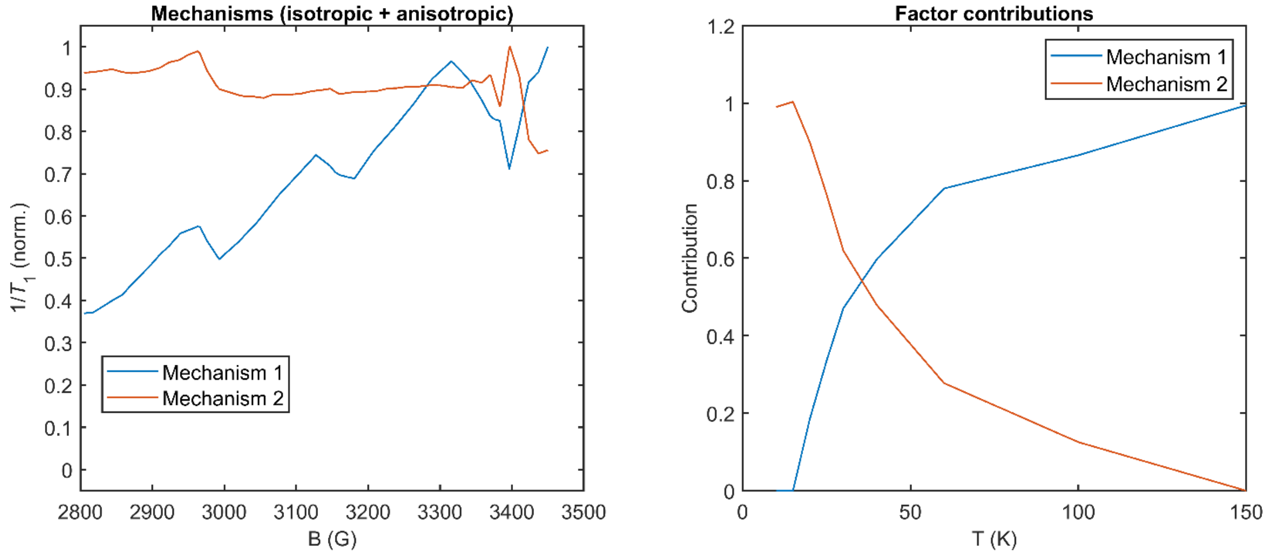

Figure S29: Cu(acac)$_2$ best-fit T_1 anisotropy mechanisms.

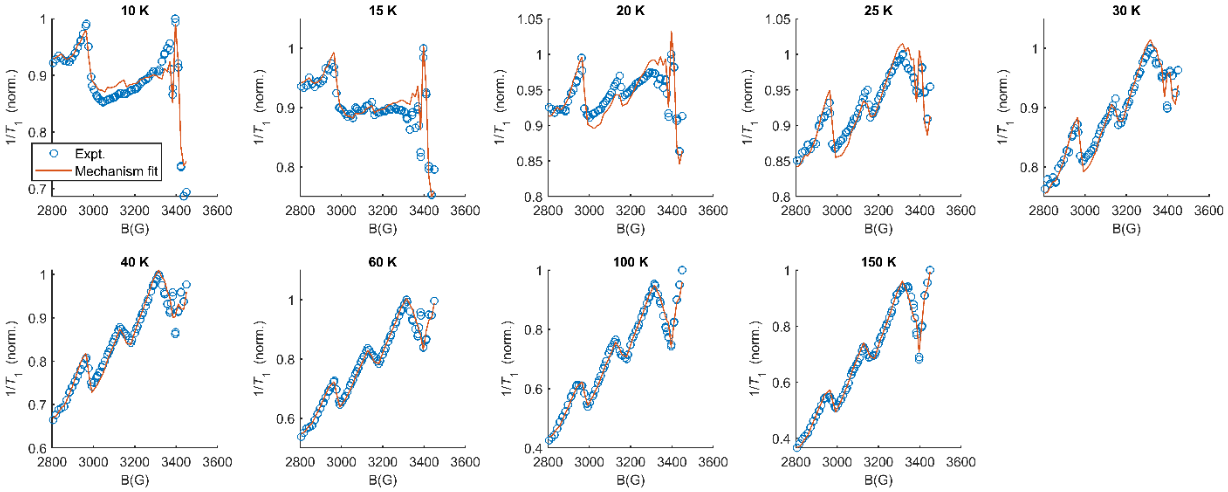

Figure S30: Cu(acac)$_2$ T_1 anisotropy data fit by mechanisms.

Figures S31 – S42 detail the factor analysis decomposition for CuOEP. Three factors were considered necessary to reproduce the salient features of the T_1 anisotropy data.

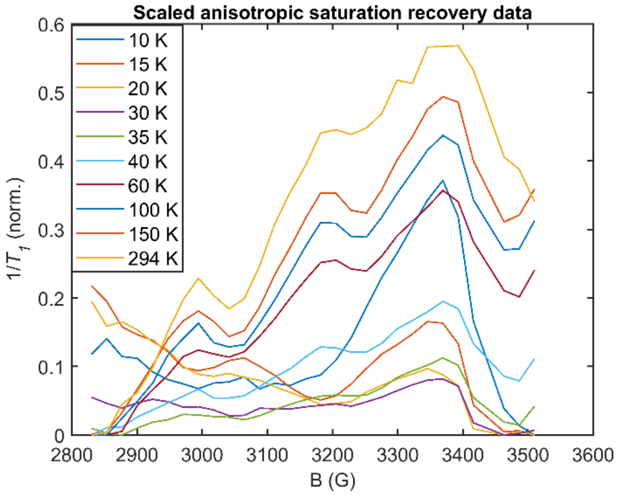

Figure S31: Normalized anisotropic component of the saturation recovery T_1 data for CuOEP (computed as $D/max(D) - min(D/max(D))$ for each temperature point).

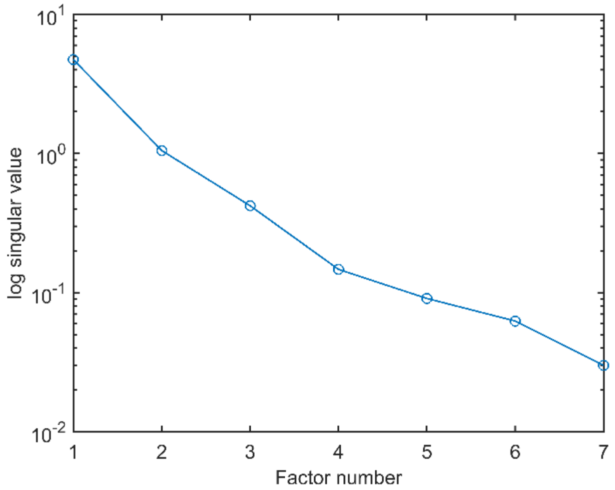

Figure S32: Scree plot of the SVD singular values for CuOEP T_1 data given in **Figure S31**.

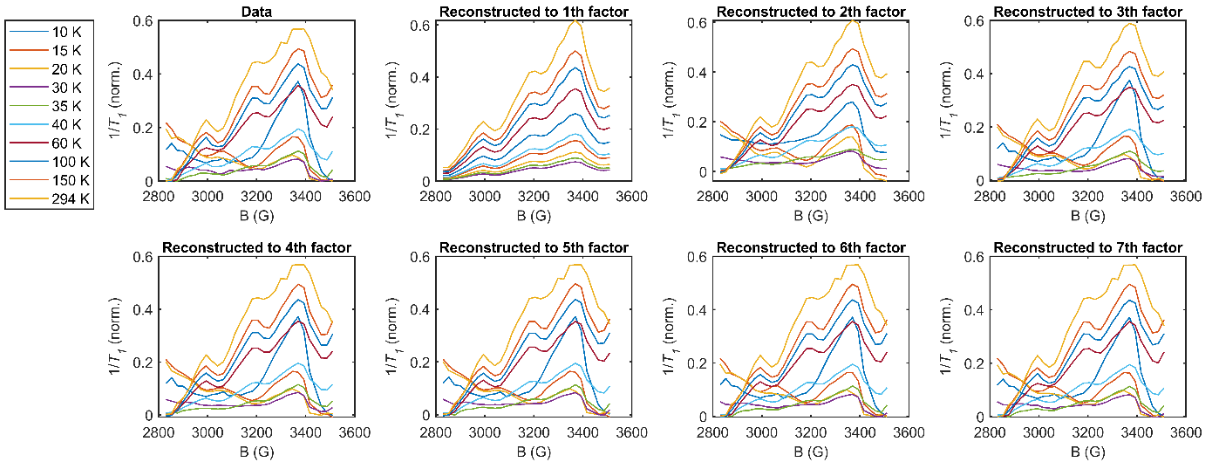

Figure S33: CuOEP data reconstruction for various rank values used in SVD.

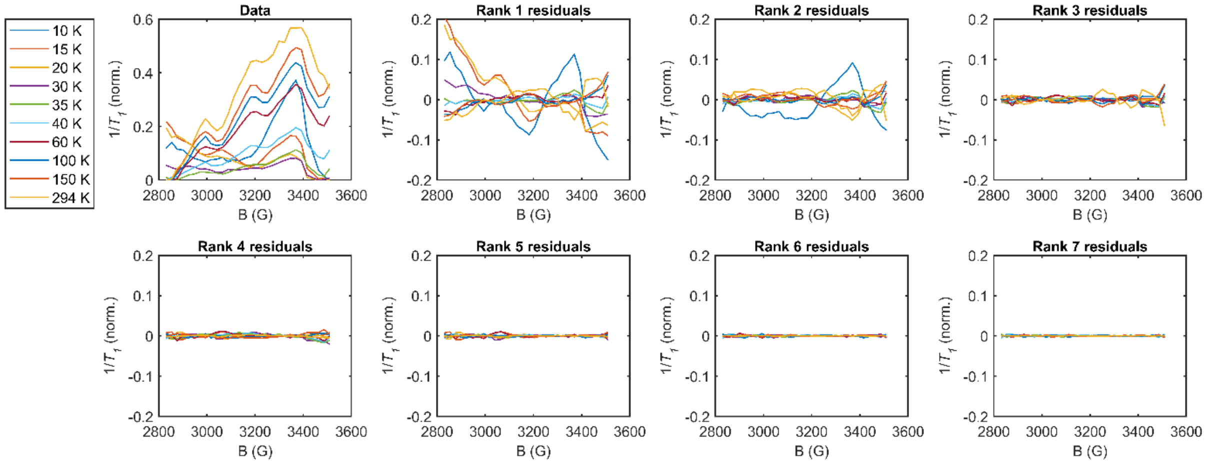

Figure S34: CuOEP residuals for various rank values used in SVD.

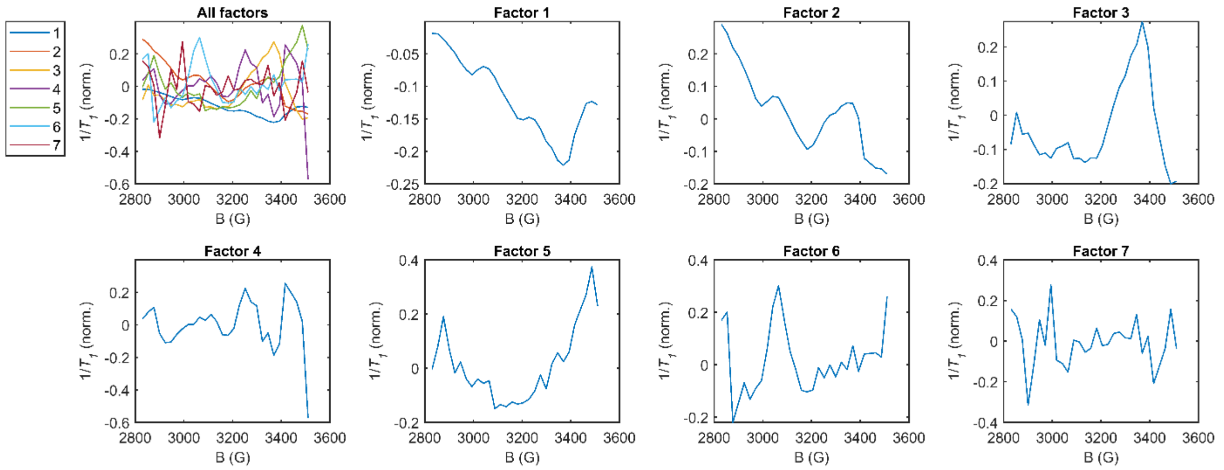

Figure S35: CuOEP factor spectra for various rank values used in SVD.

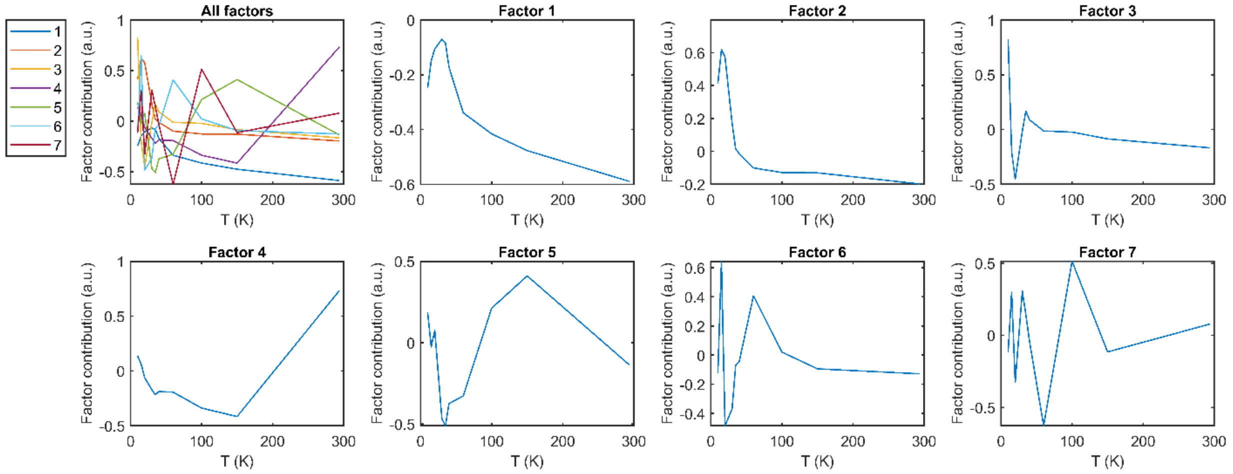

Figure S36: CuOEP factor contributions for various rank values used in SVD.

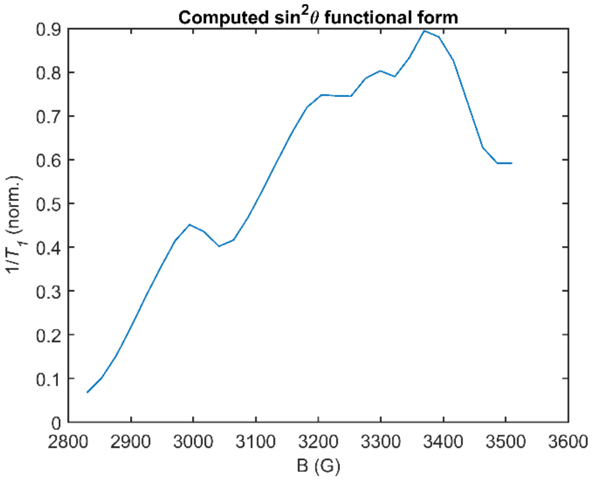

Figure S37: Computed $\sin^2\theta$ functional form for CuOEP.

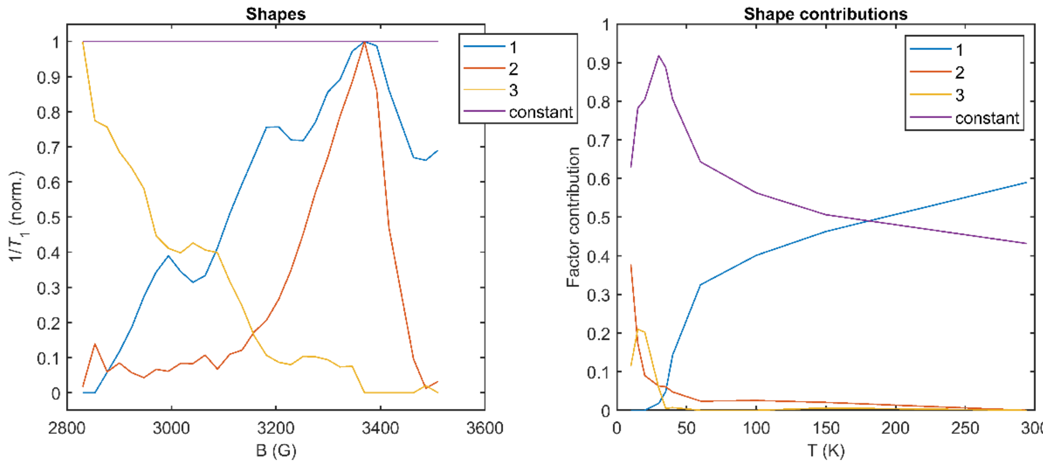

Figure S38: CuOEP T_1 anisotropy shapes from alternating least-squares factor decomposition.

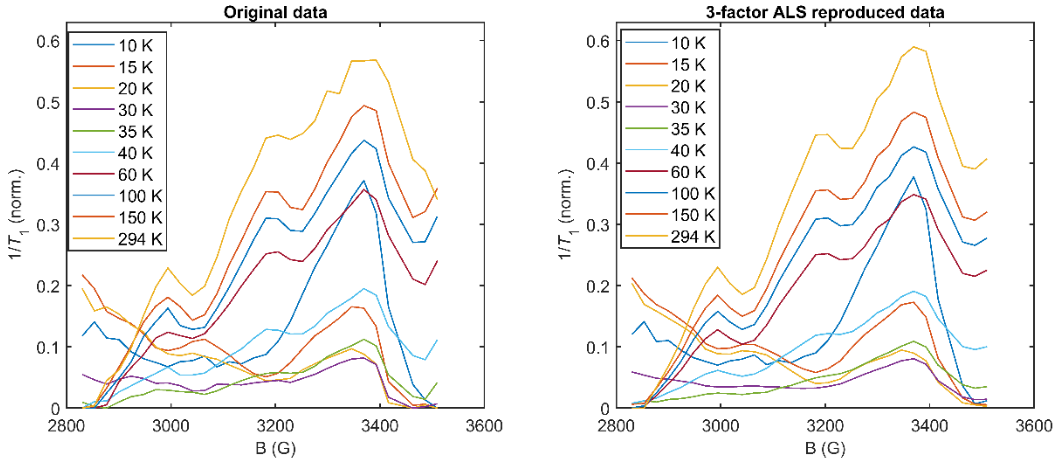

Figure S39: CuOEP data reproduction from alternating least-squares factor decomposition (T_1 anisotropy shapes).

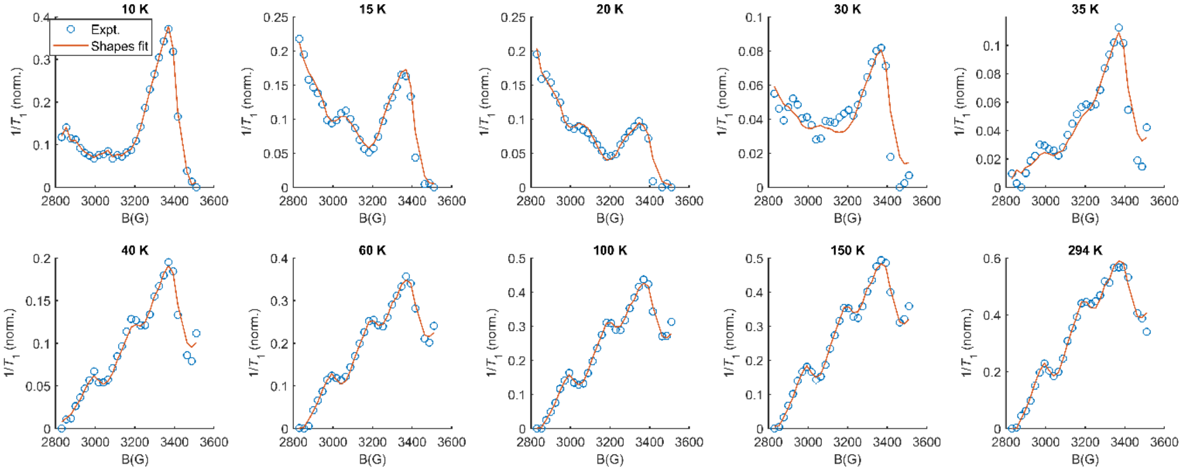

Figure S40: CuOEP data fit from alternating least-squares factor decomposition (T_1 anisotropy shapes).

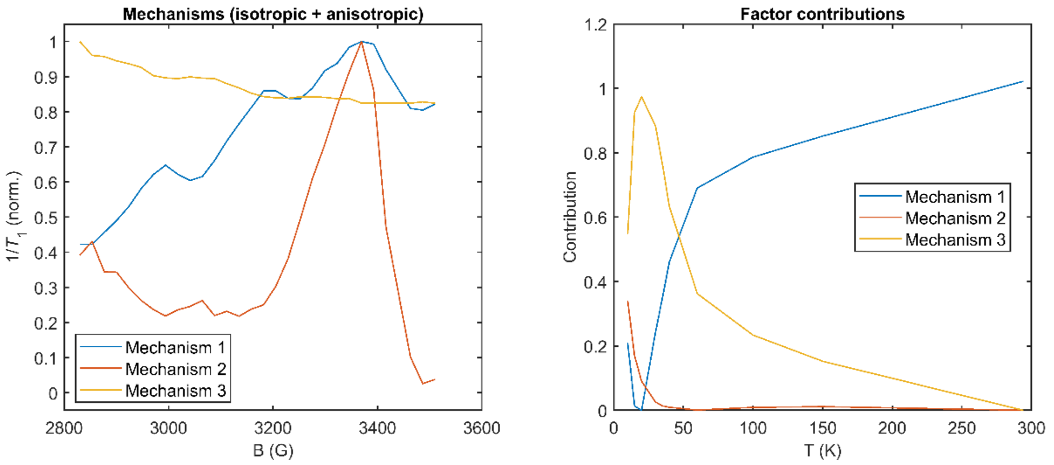

Figure S41: CuOEP best-fit T_1 anisotropy mechanisms.

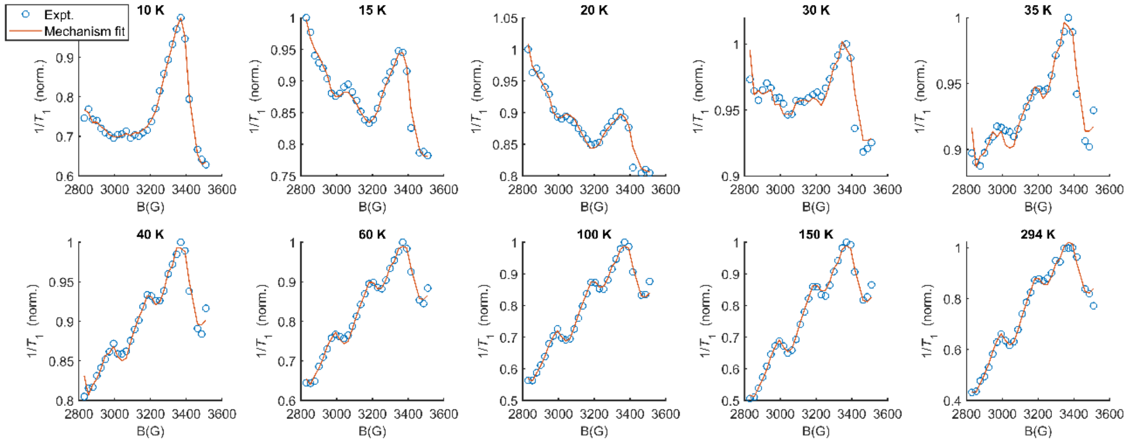

Figure S42: CuOEP T_1 anisotropy data fit by mechanisms.

9. Single crystal pulse electron paramagnetic resonance

All single-crystal pulse EPR experiments were conducted with 32 ns $\pi/2$ pulses and 64 ns π pulses. Shorter pulses were found to reduce resolution in the EDFS spectrum, as the pulse bandwidth exceeded the inhomogeneous broadening of the individual hyperfine manifold transitions.

Single-crystal samples of 0.1% Cu(acac)$_2$ co-crystallized in Pd(acac)$_2$ were mounted in two ways. For the data presented in the main text, a quartz rod with 4 mm outer diameter was prepared with a flat cut-out region (**Figure S43**). The cut-out was coated with Corning vacuum grease, and the crystal was gently placed on top. The grease acts as an EPR-silent crystal adhesive that solidifies at cryogenic temperatures. The quartz rod was then attached to the sample rod of the Bruker E580 pulse EPR spectrometer in an identical manner to a usual X-band tube. This mounting has the advantage of accommodating large crystals, but may induce an offset in the crystal from the center of the resonator depending on crystal thickness. To ensure that the observed T_1 angular dependence was not an artifact of angle-dependent cavity coupling, a second crystal was mounted in the end of an open quartz capillary (I.D. 1.2 mm). The capillary end was plugged with vacuum grease, and the crystal was submerged into the grease. This mounting strategy ensures the crystal is as close as possible to the sample rod axis of rotation. Both sample mounting strategies display the same T_1 anisotropy behavior, demonstrating that single-crystal T_1 anisotropy is not an artifact of cavity coupling.

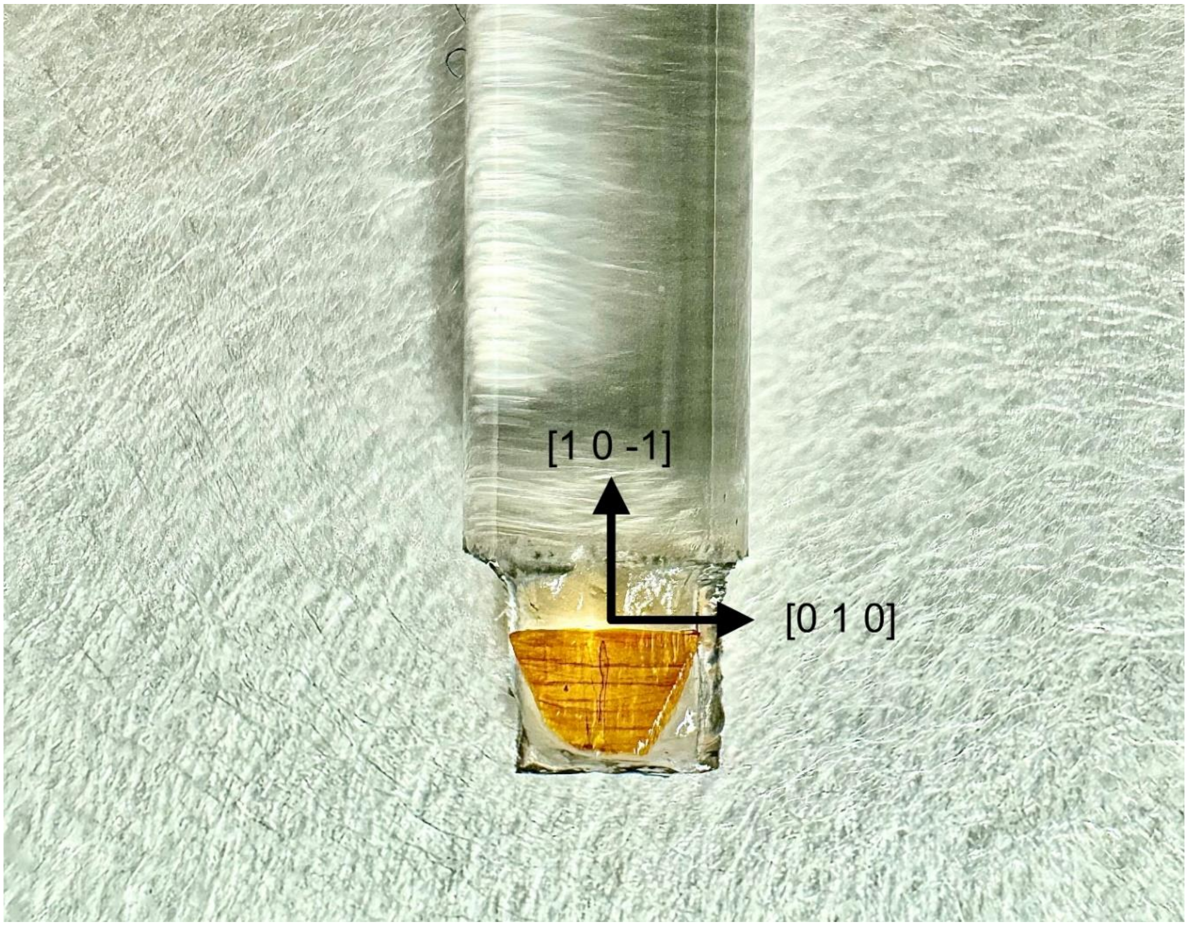

Figure S43: 0.1% Cu(acac)$_2$ in Pd(acac)$_2$ sample mounted on quartz rod cut-out for EPR spectroscopy. [1 0 1] direction lies out of the plane of the page (the flat face of the crystal).

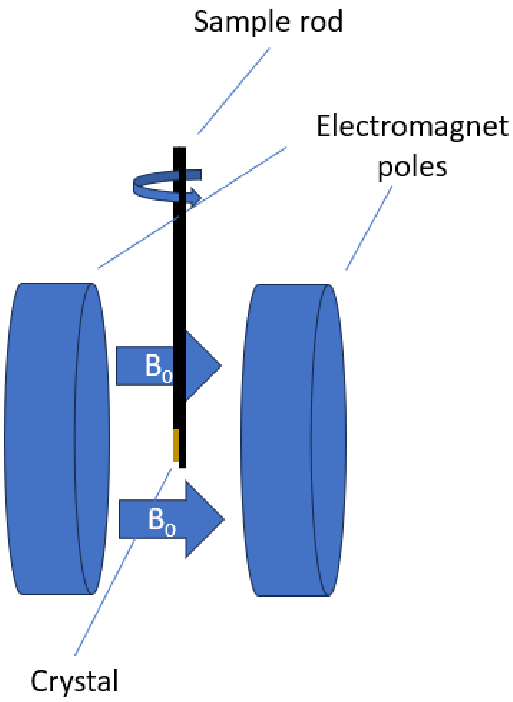

Figure S44: Geometry of crystal mounting in the EPR spectrometer.

The unit cells of Cu(acac)$_2$ and Pd(acac)$_2$ are very close to isostructural and each contain two metal centers in sites related by symmetry (**Figure S45**).[3,12] To acquire single-crystal T_1 anisotropy directly comparable to the powder anisotropy, it is essential to choose an axis of rotation such that both parallel and perpendicular orientations relative to B_0 will be obtained through the course of a 360 degree sample rod rotation (**Figure S44**). The [1 0 -1] direction satisfies the desired rotation properties (**Figures S46-S47**). Samples were mounted on the rod so that the [1 0 -1] axis was parallel to the sample rod axis. We denote the laboratory-frame angle of the quartz sample rod around the vertical axis as Ω. When B_0 is collinear with [1 0 1], we define Ω = 0°. When B_0 is collinear with [0 1 0], then Ω = 90°. Two sets of four intense lines are in general expected, corresponding to the two Cu orientations within the crystal (**Figure S48**)

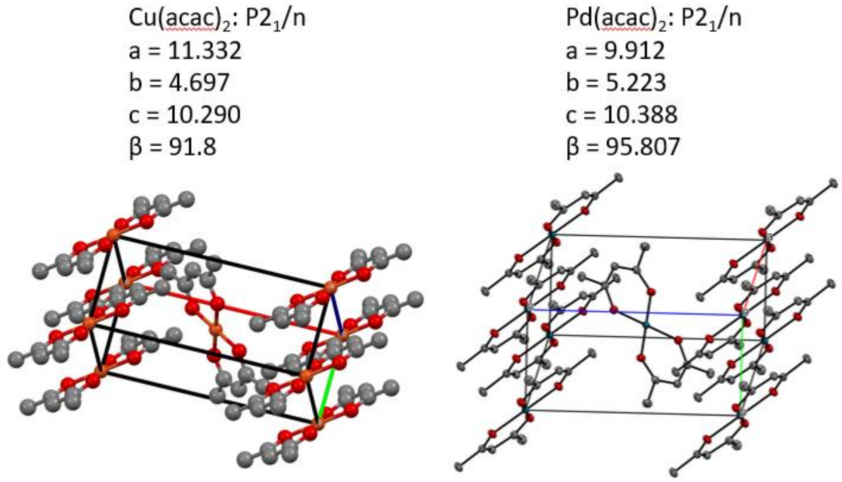

Figure S45: Unit cells of $Cu(acac)_2$ and $Pd(acac)_2$.

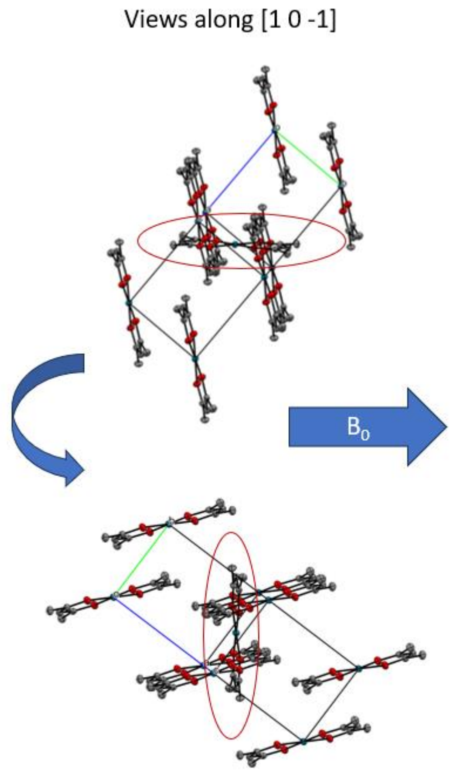

Figure S46: $Pd(acac)_2$ unit cell viewed along the [1 0 -1] direction (pointing into the page). Rotation of the crystal accesses both perpendicular (top) and parallel (bottom) orientations relative to the magnetic field (in the plane of the page).

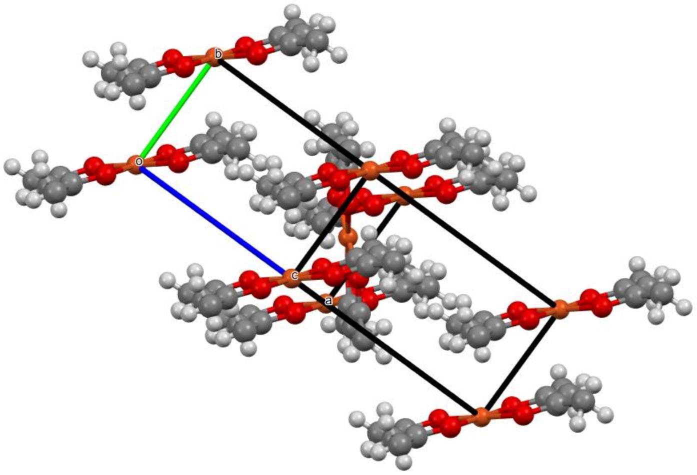

Figure S47: Cu(acac)$_2$ unit cell viewed along the [1 0 -1] direction (pointing into the page). Rotation of the crystal accesses both perpendicular (top) and parallel (bottom) orientations relative to the magnetic field (in the plane of the page).

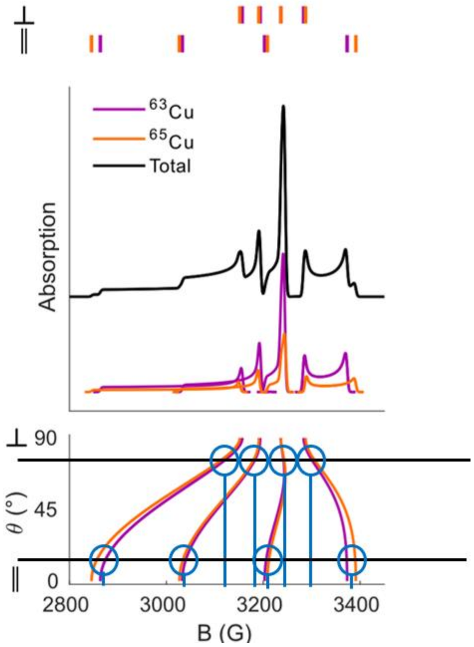

Figure S48: Schematic correspondence of a single-crystal EPR spectrum to the orientation-averaged powder-EPR spectrum.

Different crystal orientations were accessed by manually rotating the sample rod in the spectrometer, and the angle was recorded. Taking into account the precision of mounting along [1 0 -1] and the precision of rotating the rod, this laboratory-frame angle determination is precise to +/–5-10°. The EDFS spectrum was subsequently employed to refine this angle determination (**Figure S49-S50**). The spin Hamiltonian of the powdered 0.1% Cu(acac)$_2$ in Pd(acac)$_2$ has previously been reported. Using the EasySpin 5 software,[13] the

crystal orientation Euler angles were altered until the simulated peaks for a given Cu site match the observed parameters. These refined angles are accurate to +/–2°.

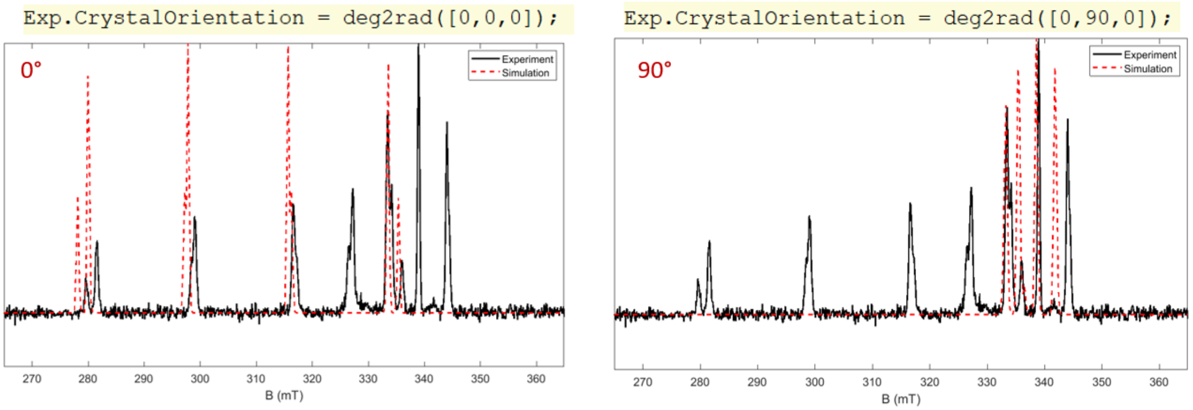

Figure S49: Initial guesses for the molecular frame angle based on the EDFS spectrum.

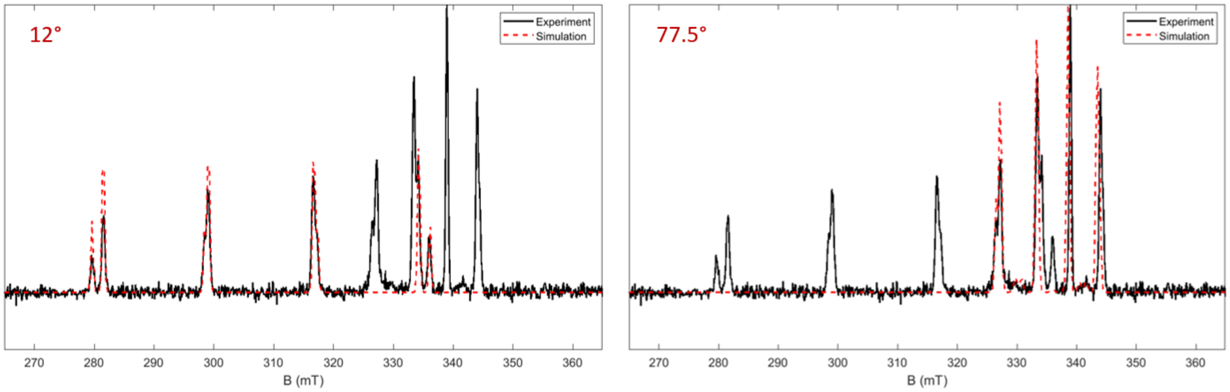

Figure S50: Refined molecular frame angles based on the EDFS spectrum, obtained by altering the second Euler angle in EasySpin to rotate between x and z orientations.

The EDFS spectra themselves can only be used to determine molecular angles θ within the range 0° – 90°, as angles greater than 90° contain an equivalent spectrum to some angle between these bounds. However, because the laboratory-frame angles Ω were also recorded, it is possible to reconstruct the refined molecular angles θ with complete phase information, which we refer to as phase-unwrapping. For a given value of θ fit from the EDFS angle, this may be done by constructing the set S of all θ yielding equivalent EDFS spectra modulo the 90° phase ambiguity. We denote the molecular frame EDFS angle between 0 and 90° as $\tilde{\theta}$ and the reconstructed molecular angle with full phase information as simply θ. The equivalent angles set is constructed as $S = \{\tilde{\theta} + k * 180°, -\tilde{\theta} + k * 180°\}$ for all integers k. Then $\theta_0 = \tilde{\theta}_0$ or $-\tilde{\theta}_0$, and $\theta_{i+1} = argmin_{s_{i+1}} \|(\theta_i + \Omega_{i+1} - \Omega_i) - s_{i+1}\|$ for $s \in S$. This algorithm computes the increment by which θ should increase on the basis of the laboratory frame rod rotation $\Omega_{i+1} - \Omega_i$, finds the closest angle s_{i+1} equivalent to the angle $\tilde{\theta}_{i+1}$ calculated from the EDFS fit, and assigns this as the molecular angle θ_{i+1} possessing full rotational phase information. The starting phase of $\tilde{\theta}_0$ or $-\tilde{\theta}_0$ for the Cu_A site was chosen by observing which choice gave a total rotation angle most similar to that computed from the total rod rotation angle recorded across the full set of experiments. The Cu_B site phase was initialized at the closest equivalent angle to $\theta_0(Cu_A)$ + 90°, in accordance with the molecules being at right angles in the crystal geometry. The phase-unwrapped angles θ_i are employed for the remainder of the single crystal analysis.

An example of the angle reconstruction is given below for the 100 K data (**Figure S51**):

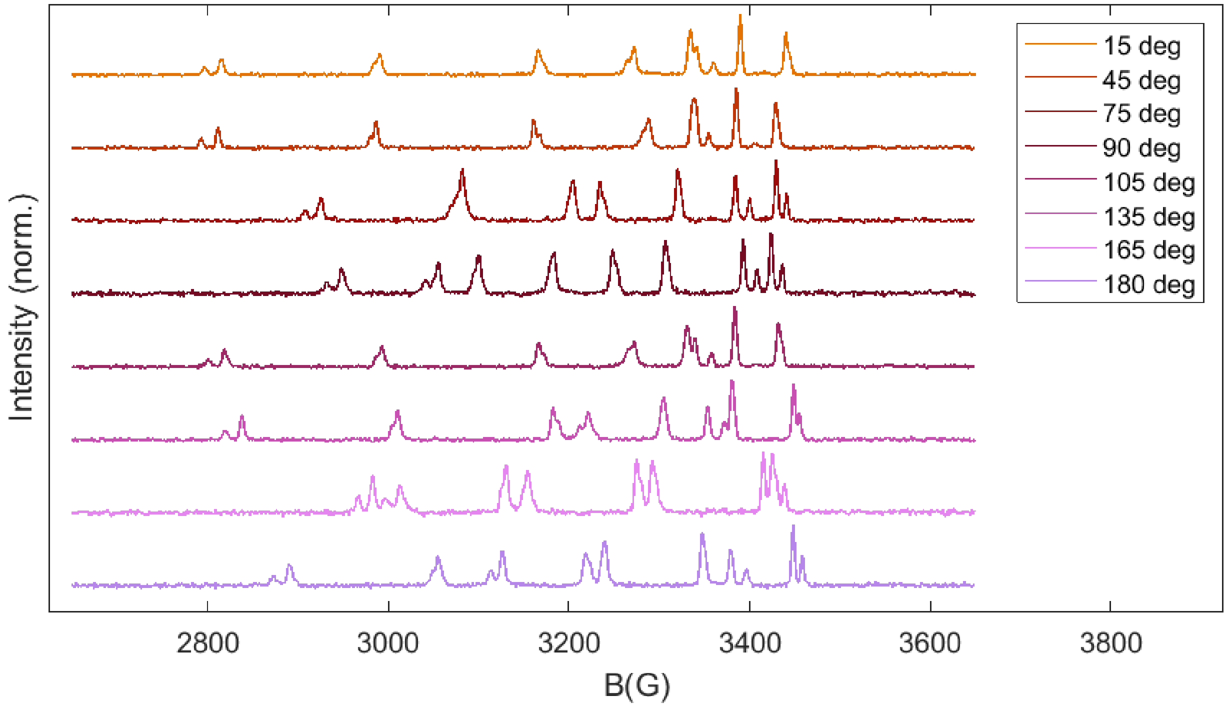

Figure S51: 100 K single-crystal EDFS spectra given for several rod orientations Ω. Angles are recorded in the laboratory frame (see above).

Table S4: Laboratory-frame rod angles (Ω), EDFS-refined molecular-frame angles ($\tilde{\theta}$), and phase-unwrapped molecular-frame angles (θ) for 100 K single-crystal T_1 anisotropy.

Ω (°)	$\tilde{\theta}$ (Cu_A) (°)	$\tilde{\theta}$ (Cu_B) (°)	θ (Cu_A) (°)	θ (Cu_B) (°)
15	12	76.5	-12	76.5
45	10	78	10	102
75	34	54	34	126
90	37.5	51	51	142.5
105	12	75.5	75.5	168
135	19	70	110	199
165	43	46.5	137	226.5
180	30	59.5	150	239.5

Table S5: Laboratory-frame rod angles (Ω), EDFS-refined molecular-frame angles ($\tilde{\theta}$), and phase-unwrapped molecular-frame angles (θ) for 20 K single-crystal T_1 anisotropy. A global phase offset of −180° is applied to the phase-unwrapped angles θ to center the data around 0°.

Ω (°)	$\tilde{\theta}$ (Cu_A) (°)	$\tilde{\theta}$ (Cu_B) (°)	θ (Cu_A) (°)	θ (Cu_B) (°)
135	13	76	-167	-76
165	36.5	54	-143.5	-54
180	36	54	-126	-36

-165	17.5	72	-108	-17.5
-135	5	86	-86	5
-105	33	56.5	-56.5	33
-90	38	51	-38	51
-75	22	67	-22	67
-45	11	78	11	102
-15	38	52.5	38	127.5
0	35	55	55	145
15	15	73	73	165
45	2	86	94	182
75	31.5	57	123	211.5
90	36	53	144	233
105	14	74	166	254
135	8	80	188	280
165	40.5	48.5	220.5	311.5

Table S6: Laboratory-frame rod angles (Ω), EDFS-refined molecular-frame angles ($\tilde{\theta}$), and phase-unwrapped molecular-frame angles (θ) for 10 K single-crystal T_1 anisotropy.

Ω (°)	$\tilde{\theta}$ (Cu_A) (°)	$\tilde{\theta}$ (Cu_B) (°)	θ (Cu_A) (°)	θ (Cu_B) (°)
15	30	59.5	-30	59.5
45	5	82	5	82
75	25.5	63	25.5	117
90	36.5	52	36.5	128
105	19.5	69	69	160.5
135	12	76	104	192
165	40.5	48.5	131.5	220.5

The values of θ for the Cu_A and Cu_B sites reliably sum to a value within 1-2° of 90°, indicating that the two Cu molecules are at close to a right angle orientation. In the $Pd(acac)_2$ unit cell, the planes defined by the first coordination sphere of the PdO_4 atoms of the two molecules form an angle of 78.8° (**Figure S52**). In the $Cu(acac)_2$ unit cell, however, the planes defined by CuO_4 form an angle of 89.2° (**Figure S53**). This indicates that the $Cu(acac)_2$ sites in the 1:1000 Cu:$Pd(acac)_2$ co-crystal obey a packing more similar to the pure $Cu(acac)_2$ structure than the pure $Pd(acac)_2$ structure, a surprising result. This inevitably introduces a small uncertainty in the orientation of the $Cu(acac)_2$ units relative to the $Pd(acac)_2$ lattice planes probed by face indexing. However, the ability to obtain single-crystal $Cu(acac)_2$ spectra in agreement with pure perpendicular and pure parallel fields determined from the powder spectrum demonstrates that the $Cu(acac)_2$ have been oriented as desired.

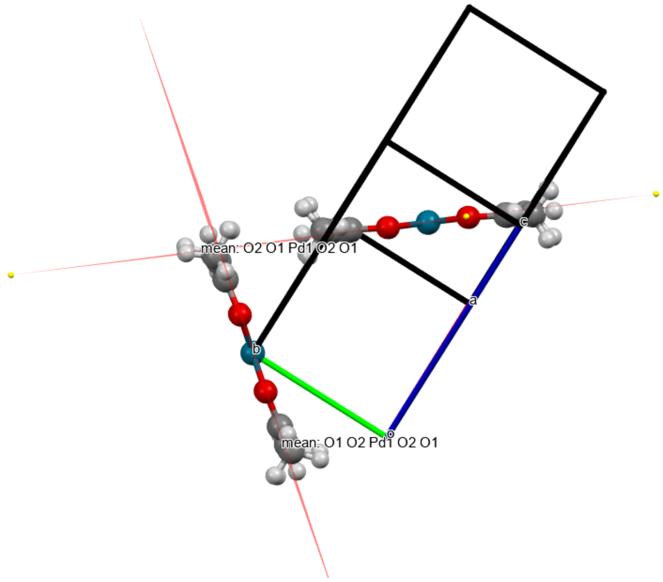

Figure S52: Pd(acac)$_2$ first-coordination-sphere planes, which form an angle of 78.8°.

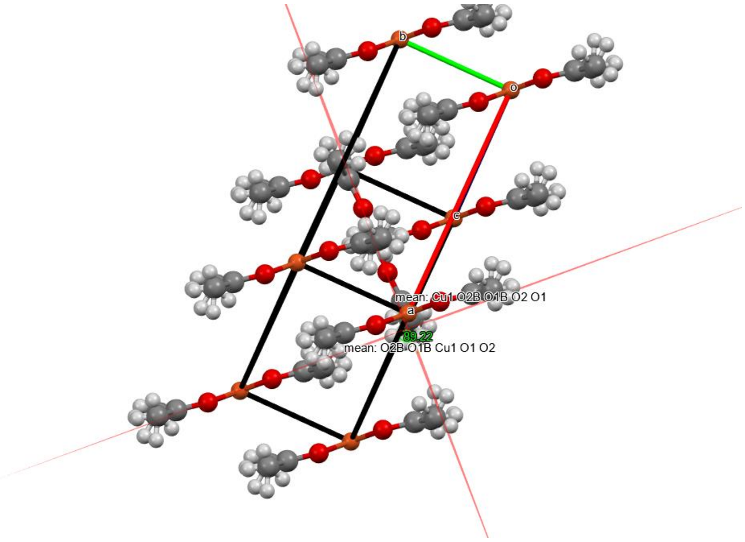

Figure S53: Cu(acac)$_2$ first-coordination-sphere planes, which form an angle of 89.2°.

Finally, we examined the degree of fidelity with which x and z orientations are interchanged by rotation about [1 0 -1]. As is apparent from **Figures S46-S47**, the Cu(Pd)O$_4$ first coordination spheres do not lie exactly on end when viewed along [1 0 -1]. There will therefore be some component of the rotation that is not exactly in the molecular xz plane. We computed the principal axes of the molecular g-tensor (**Supporting Information Section 12**) and aligned them to the two Cu sites in the Cu(acac)$_2$ crystal structure. We then simulated the alignment of the crystal with respect to B_0 and the sample rod, and rotated the rod around [1 0 -1] while examining the angle formed between B_0 and the three principal g-tensor axes (or, equivalently, the vector projections). As expected, the majority (~90%) of the angular variation can be described by a rotation in the xz plane of the g-tensor frame (**Figures S54-S55**). However, there exists a minor component of the rotation which gives a nonzero projection of B_0 onto g_y. Notably, this projection

minimizes at Ω = −22°, while the projections of g_x and g_z minimize at −135° and −45°, respectively. Rotation in the molecular xy plane is therefore phase-shifted relative to rotation in the molecular xz plane for the [1 0 -1] direction of the $Cu(acac)_2$ structure. One could therefore conceive that the phase shifts ϕ observed at 20 K and 10 K in the spin relaxation rates arise from enhanced T_1 anisotropy in the xy plane, with the spin relaxation tensor principal axes still aligned to the molecular frame axes.

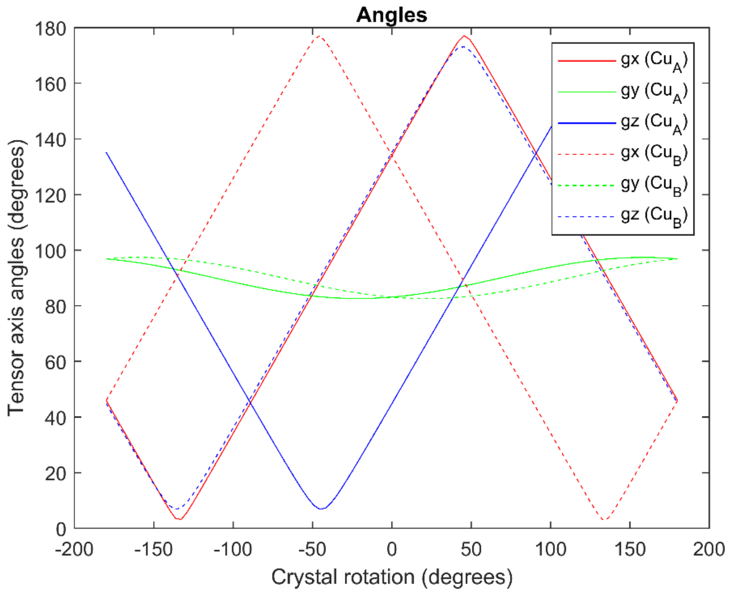

Figure S54: Simulated angles between the $Cu(acac)_2$ molecular g-tensor principal axes and B_0 when rotating the $Cu(acac)_2$ crystal structure around the [1 0 -1] axis.

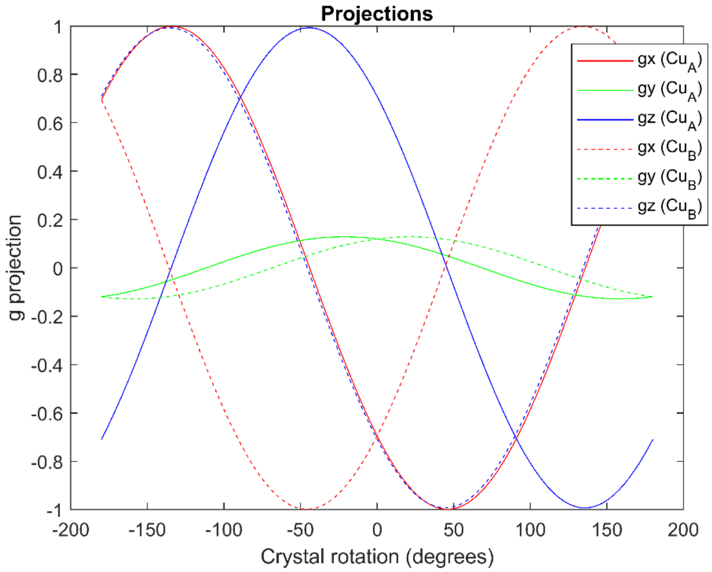

Figure S55: Simulated projections along B_0 of the $Cu(acac)_2$ molecular g-tensor principal axes when rotating the $Cu(acac)_2$ crystal structure around the [1 0 -1] axis.

Three considerations indicate this is a less-likely explanation than the proposed mechanism involving rotation of the spin relaxation tensor axes themselves (**Figure 4F-G)**. First, given the small magnitude of the projection along g_y (maximum projection of 0.13), the innate anisotropy in the xy plane would have to be very large to account for the observed spin relaxation anisotropy magnitude. For the T_1 anisotropy ratio of ~1.7 observed at 20 K, it would be necessary for the intrinsic xy anisotropy ratio to be approximately 1.7/0.13 = 13. Anisotropy ratios up to 10 do have precedent,[14] but much more commonly the T_1 anisotropy is in the single digits.[1] Second, the phase shift between the xy rotation and the xz rotation is 23° as discussed above, but larger phase shifts ϕ are observed experimentally (28° at 20 K, and 42° at 10 K) (**Figures 4E, S64**), which could not easily be accessed under this mechanism. Third, when the spin relaxation rate is plotted as a function of the unrefined laboratory frame orientation Ω, the relaxation curves of the two copper sites clearly begin to overlay at 20 K and 10 K, whereas they are separated at 100 K (**Figures S56 – S58**). This indicates that orientation of the crystal planes is a good predictor of the spin relaxation rate, consistent with the interpretation that the spin relaxation tensor axes conform to the symmetry of the unit cell at low temperatures. By contrast, at 100 K, the spin relaxation tensor clearly does not conform to the crystal orientation, and the relaxation properties of the different Cu sites are strongly offset.

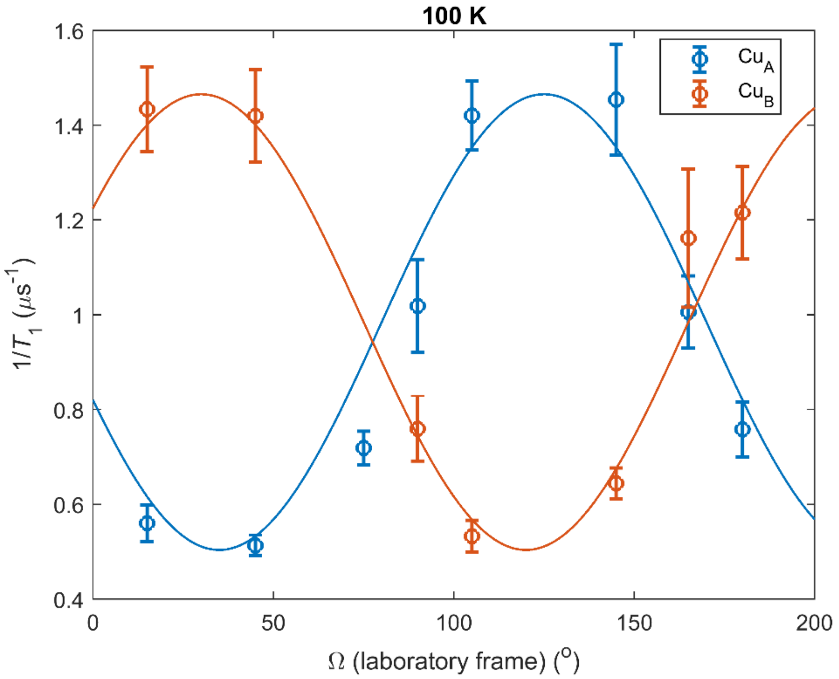

Figure S56: Relaxation rate (M_I = -3/2) vs. unrefined laboratory frame sample rod angle at 100 K. The curves for Cu_A and Cu_B are maximally offset by about 90°, indicating that the macroscopic crystal orientation does not control spin relaxation.

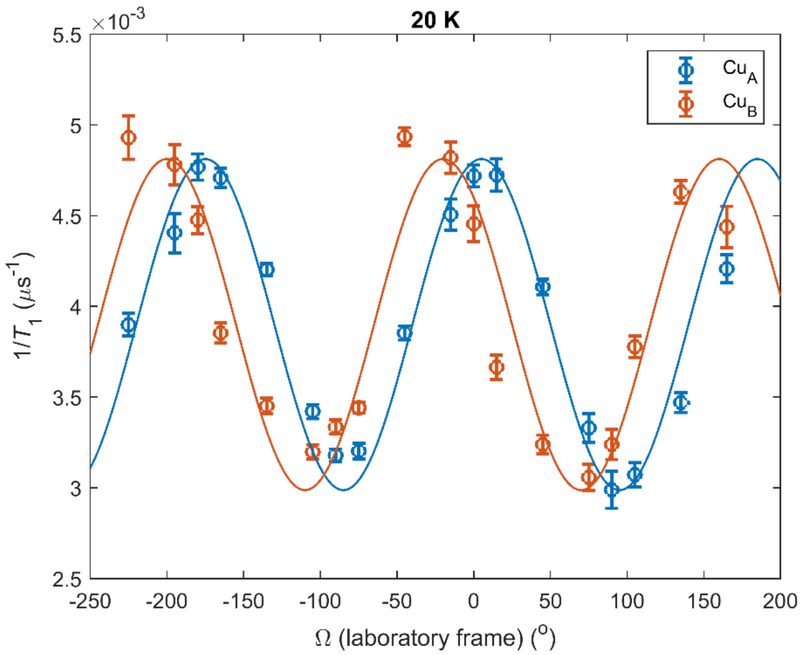

Figure S57: Relaxation rate (M_I = -3/2) vs. unrefined laboratory frame sample rod angle at 20 K. The curves for Cu_A and Cu_B are offset by about 35°, indicating that the macroscopic crystal orientation partially determines spin relaxation.

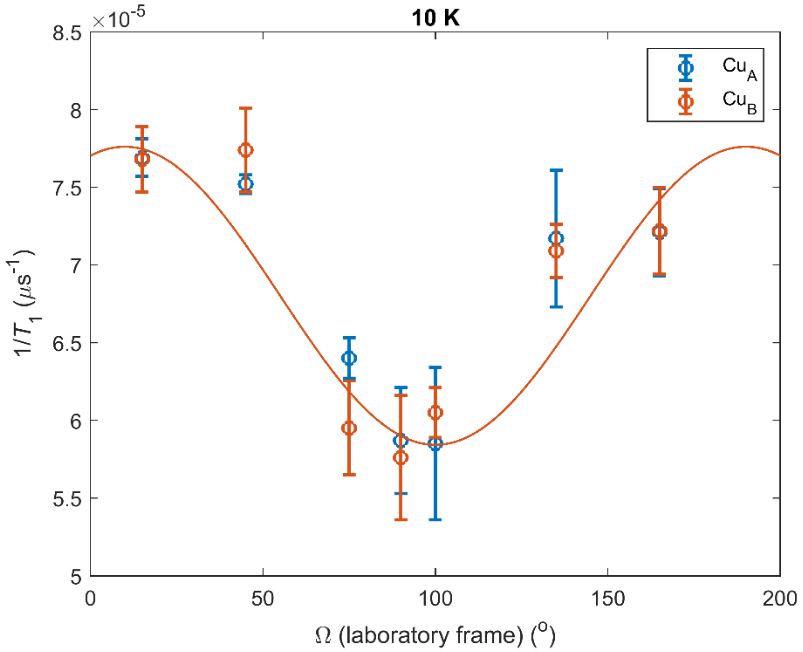

Figure S58: Relaxation rate (M_I = -3/2) vs. unrefined laboratory frame sample rod angle at 10 K. The curves for Cu_A and Cu_B are the same within measurement uncertainty, indicating that the macroscopic crystal orientation completely determines spin relaxation.

Regardless, the details of the low-temperature $1/T_1$ phase shift do not significantly affect the conclusions of this study. Even if xy anisotropy contributes to the observed signal < 20 K, the symmetry of the spin relaxation tensor still must deviate from the axial symmetry of the molecular frame (with y, rather than z, constituting the new axial axis). This change in spin relaxation symmetry points to a qualitatively distinct mechanism of spin-phonon coupling. Future experiments involving rotation about multiple crystal axes will be able to conclusively disambiguate these possibilities.

10. Discussion of the spin relaxation tensor

The single-crystal T_1 anisotropy data in **Figure 4** has been analyzed by proposing the concept of a Cartesian spin-relaxation tensor. This tensor is distinct from zero-field spin relaxation tensors,[15] the use of spherical tensor operators in Liouville space to describe spin relaxation,[16] or a tensor description of spin relaxation across a condensed-phase bandstructure.[17] Here, we provide a precise definition of our spin relaxation tensor concept, and show through a simplified model how it can give rise to the experimentally-observed single-crystal T_1 anisotropy data.

In magnetic resonance spectroscopy, tensors commonly specify energetic interactions that vary with the orientation of a molecule. Tensors can be thus be visualized by 3D ellipsoids specifying how the energetic interaction varies in space.[18] The semimajor and semiminor axes of the ellipsoids define the principal axes of the tensor, while the length of those axes dictate the magnitude of that interaction along that direction in space (the principal values). In EPR spectroscopy, this interaction could be the magnitude of the Zeeman splitting or the hyperfine coupling. In NMR spectroscopy, this interaction could be the magnitude of the chemical shift.

In the present work, we propose to define a tensor where the corresponding surface specifies the rate of spin relaxation along that direction in space. The spin relaxation tensor will be a property of the sample. If the principal axes of the spin relaxation tensor align with coordinate axes of the molecule, then we say that the spin relaxation tensor "adopts the orientation" of the molecule. If the principal axes of the spin relaxation tensor align with the lattice planes of the unit, then we say it adopts the orientation of the lattice.

Formally, a rank-2 tensor is a real-valued linear function of two vectors. This is a mathematical object that takes two vectors as an input and returns a scalar as the output, and possesses distinctive transformation properties.[19] We define the spin relaxation tensor as a rank-2 tensor such that the input vectors are (i) the vector of the applied magnetic field, and (ii) the direction in which spin relaxation is measured, and the output is the rate of spin relaxation. In these experiments, we always measure spin relaxation along the applied magnetic field, so the two input vectors will have the same orientation. Define $\widehat{B_0}$ as the normalized vector corresponding to the applied field direction, and define **T** as the spin relaxation tensor. (Note that **T** is not related to the axial component of the hyperfine interaction, another common usage of this symbol in EPR.) The extraction of the scalar relaxation rate from the rank-2 tensor can be represented by the following matrix-vector multiplication equation:

$$\frac{1}{T_1} = \widehat{B_0}^T \cdot \mathbf{T} \cdot \widehat{B_0} = \begin{pmatrix} \widehat{B_0}_x & \widehat{B_0}_y & \widehat{B_0}_z \end{pmatrix} \begin{pmatrix} T_{xx} & T_{yx} & T_{zx} \\ T_{xy} & T_{yy} & T_{zy} \\ T_{xz} & T_{yz} & T_{zz} \end{pmatrix} \begin{pmatrix} \widehat{B_0}_x \\ \widehat{B_0}_y \\ \widehat{B_0}_z \end{pmatrix} \quad \text{(S3)}$$

The basis directions of x, y, and z are, in general, arbitrary. If the basis is chosen to align with the principal axes of the spin relaxation tensor, then the matrix representation of the tensor will be diagonalized.

For simple illustration, let us consider an axial system such that x is equivalent to y, and show how the spin relaxation tensor concept explains the single-crystal T_1 anisotropy results. In this scenario, the matrix representation of **T** is 2 x 2 and each vector has two components, corresponding to x and z. Let us choose the basis corresponding to the x and z orientations of the axial molecule (**Figure S59**).

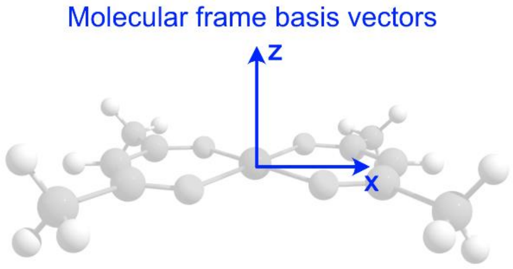

Figure S59: Basis vectors defining the molecular frame of an axial molecule. For the D_{2h} symmetry molecule $Cu(acac)_2$, the *g*-tensor principal axes are exactly aligned with the molecular frame basis vectors.

Let us further assume that these correspond to the orientations of the fastest and slowest spin relaxation rates, as shown schematically (**Figure S60**):

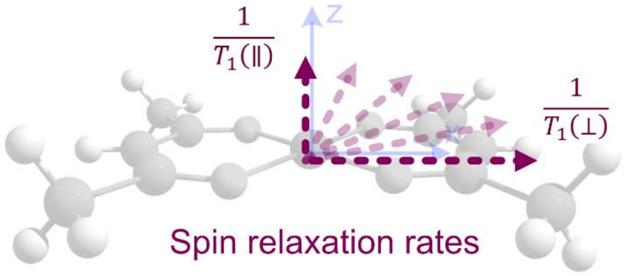

Figure S60: Depiction of the spin relaxation rate for different orientations. The direction of the arrows indicates the direction along which B_0 is applied and spin relaxation is measured. The magnitude of the arrows indicates the magnitude of the spin relaxation rate at that orientation.

This matches the situation for $Cu(acac)_2$ at 100 K. Then, the matrix representation of the spin relaxation tensor is simply:

$$\mathbf{T} = \begin{pmatrix} T_x & 0 \\ 0 & T_z \end{pmatrix} \tag{S4}$$

To calculate the spin relaxation rate along the x-direction of the molecule, we choose $\widehat{B_0}^T = (1 \quad 0)$. So:

$$\frac{1}{T_1(\perp)} = (1 \quad 0)\begin{pmatrix} T_x & 0 \\ 0 & T_z \end{pmatrix}\begin{pmatrix} 1 \\ 0 \end{pmatrix} = T_x \tag{S5}$$

And similarly for the z direction:

$$\frac{1}{T_1(\parallel)} = (0 \quad 1)\begin{pmatrix} T_x & 0 \\ 0 & T_z \end{pmatrix}\begin{pmatrix} 0 \\ 1 \end{pmatrix} = T_z \tag{S6}$$

So the principal tensor values of the spin relaxation tensor **T** give the rates of spin relaxation for the parallel and perpendicular orientations, enabling a description of T_1 anisotropy.

Now, consider a vector at an arbitrary angle θ between the z (θ = 0°) and the x (θ = 90°) orientations: $\widehat{B_0}^T = (\sin\theta \quad \cos\theta)$. Then:

$$\frac{1}{T_1(\theta)} = (\sin\theta \quad \cos\theta)\begin{pmatrix} T_x & 0 \\ 0 & T_z \end{pmatrix}\begin{pmatrix} \sin\theta \\ \cos\theta \end{pmatrix} = T_x(\sin^2\theta) + T_z(\cos^2\theta)$$
$$= (T_x - T_z)(\sin^2\theta) + T_z \qquad \text{(S7)}$$

The expectation value of the spin relaxation tensor with respect to $\widehat{B_0}$ predicts a $\sin^2\theta$ functional form for the T_1 anisotropy (**Figure S61**). This is the experimentally observed anisotropy form for $Cu(acac)_2$ at 100 K.

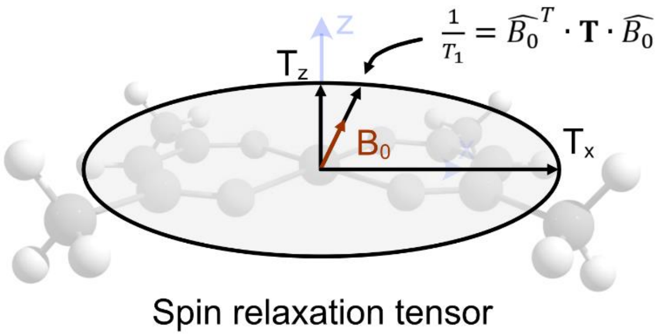

Figure S61: Schematic representation of the spin relaxation tensor. For any given vector direction of B_0, computation of the product $\widehat{B_0}^T \cdot \mathbf{T} \cdot \widehat{B_0}$ with the spin relaxation tensor yields a scalar value giving the rate of spin relaxation at that orientation. The set of all such products yields the surface depicted graphically. The exact shape is similar to but not exactly an ellipse. The long axis and the short axis are dictated by the principal tensor values T_x and T_z; these determine the largest and smallest rates of spin relaxation obtainable at any orientation.

So far, the spin relaxation tensor concept has not provided insights beyond the $\sin^2\theta$ description of T_1 anisotropy already established in the literature. We now show that the spin relaxation tensor enables rationalization of the present low-temperature single crystal anisotropy results. Let us suppose that spin relaxation is not dictated by local molecular vibrations, but by a process involving phonons traveling across lattice planes. Then, the principal axes of the spin relaxation tensor need no longer align with the principal axes of the g-tensor (i.e., the axes of the molecular frame). Instead, the spin relaxation rates should be "rotated" by an angle φ to point in the direction of the lattice planes. The spin relaxation tensor concept allows us to mathematically represent this through application of tensor rotations to **T**, as given below. Note that the basis vectors remain aligned with the molecular axes. Thus, the resulting spin relaxation tensor $\mathbf{S'}$ is no longer diagonal.

$$\mathbf{T'} = \mathbf{R}^{\boldsymbol{T}}(\boldsymbol{\varphi})\mathbf{T}\mathbf{R}(\boldsymbol{\varphi}) = \begin{pmatrix} \cos\varphi & \sin\varphi \\ -\sin\varphi & \cos\varphi \end{pmatrix}\begin{pmatrix} T_x & 0 \\ 0 & T_z \end{pmatrix}\begin{pmatrix} \cos\varphi & -\sin\varphi \\ \sin\varphi & \cos\varphi \end{pmatrix}$$
$$= \begin{pmatrix} T_x\cos^2\varphi + T_z\sin^2\varphi & \frac{1}{2}(T_z - T_x)\sin(2\varphi) \\ \frac{1}{2}(T_z - T_x)\sin(2\varphi) & T_x\sin^2\varphi + T_z\cos^2\varphi \end{pmatrix} \qquad \text{(S8)}$$

We define an arbitrary orientation of the field relative to the molecular axes, as before: $\widehat{B_0}^T = (\sin\theta \quad \cos\theta)$. Then the spin relaxation rate is given as follows. The final simplified form may be obtained by using the product-to-sum trigonometric identities.

$$\frac{1}{T_1}(\theta) = \widehat{B_0}^T \cdot \mathbf{T}' \cdot \widehat{B_0} = (\sin\theta \quad \cos\theta)\begin{pmatrix} T_x\cos^2\varphi + T_z\sin^2\varphi & \frac{1}{2}(T_z - T_x)\sin(2\varphi) \\ \frac{1}{2}(T_z - T_x)\sin(2\varphi) & T_x\sin^2\varphi + T_z\cos^2\varphi \end{pmatrix}\begin{pmatrix}\sin\theta \\ \cos\theta\end{pmatrix} \tag{S9}$$

$$= (T_x - T_z)\sin^2(\theta - \varphi) + T_z$$

Crucially, this is the exactly the same functional form for the T_1 anisotropy that is experimentally observed at 20 K for Cu(acac)$_2$ (**Figure 4E**). We see that rotation of the spin relaxation tensor away from the molecular axes gives rise to the phase shift φ (**Figure S62**). The value of φ is therefore diagnostic of the character of the relaxation process. When φ = 0°, the relaxation process proceeds through localized spin-vibrational coupling, because the spin relaxation tensor is aligned with the local molecular axes. When $\varphi \neq 0°$, the relaxation process proceeds through spin-phonon coupling with delocalized phonons, because the spin-relaxation aligns with the lattice rather than the molecular axes.

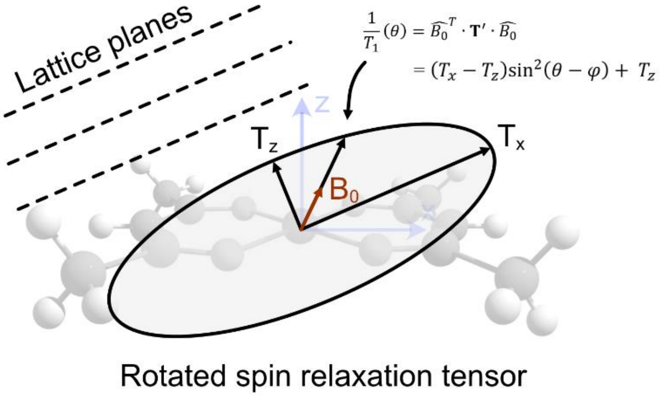

Figure S62: Schematic representation of the spin relaxation tensor rotated to align with the orientation of a key lattice plane dictating the delocalized spin-phonon coupling. The fastest and slowest rates of spin relaxation no longer correspond to the axes of the molecular frame. The phase shift φ denotes the angle between T$_z$ and z produced by the tensor rotation, while the orientation θ denotes the angle between B_0 and z. In the drawing, $\varphi \approx -15°$ and $\theta \approx +20°$.

11. Single-crystal anisotropy at 10 K

In addition to the main text experiments at 100 and 20 K, single crystal anisotropy was also acquired at 10 K. Owing to the long saturation recovery periods required, the signal-to-noise ratio is significantly worse at this temperature. However, the results may still be used to infer a phase-shift ϕ of 42 degrees, greater than at 20 K.

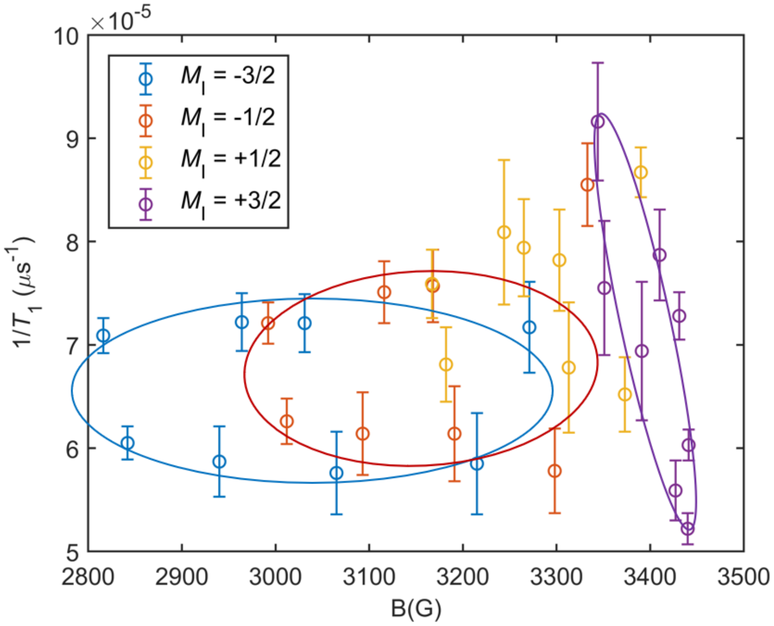

Figure S63: 10 K single-crystal T_1 anisotropy vs. B_0 for 0.1% $Cu(acac)_2$ in $Pd(acac)_2$. Elliptical traces as a guide for the eye.

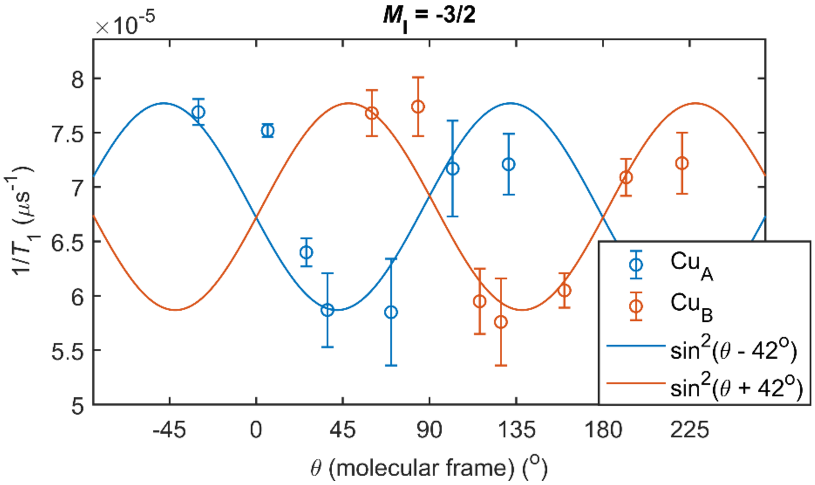

Figure S64: 10 K single-crystal T_1 anisotropy (M_I = -3/2) vs. molecular angle θ for 0.1% $Cu(acac)_2$ in $Pd(acac)_2$, and best fit $\sin^2(\theta \pm \phi)$ angular dependence.

12. Computational methods

All computations were performed in Orca 5.0.3.[20] Density functional theory (DFT) calculations employed the B3LYP functional modified with 50% exact Hartree-Fock exchange added. This calibrates the calculations to most closely match the experimental g_z value, in accordance with an established procedure.[21–23] All calculations used the RIJCOSX approximation, the ZORA relativistic correction, ZORA-def2-TZVP basis set on all atoms, SARC/J auxiliary basis set, TIGHTSCF convergence criteria, SLOWCONV convergence method, and DEFGRID3 grid precision.

A slight rhombicity has been experimentally observed in the $Cu(acac)_2$ g-tensor,[1,24] but to the best of our knowledge, existing single crystal EPR studies have not assigned the axes corresponding to the x and y *g*-values.[25–27] Therefore, to choose which value to pick for single-crystal EPR orientation refinement, we employed DFT calculations of the *g*-tensor. The calculated vs. experimental[1] *g*-values for $Cu(acac)_2$ are compared below:

Table S7: Experimental g-values for C<u>u</u>(acac)$_2$ (arranged from smallest to largest)

g_1	g_2	g_3
2.048	2.052	2.261

Table S8: DFT-calculated g-values for C<u>u</u>(acac)$_2$ (arranged according to the axes of the molecule)

g_x	g_y	g_z
2.066	2.069	2.248

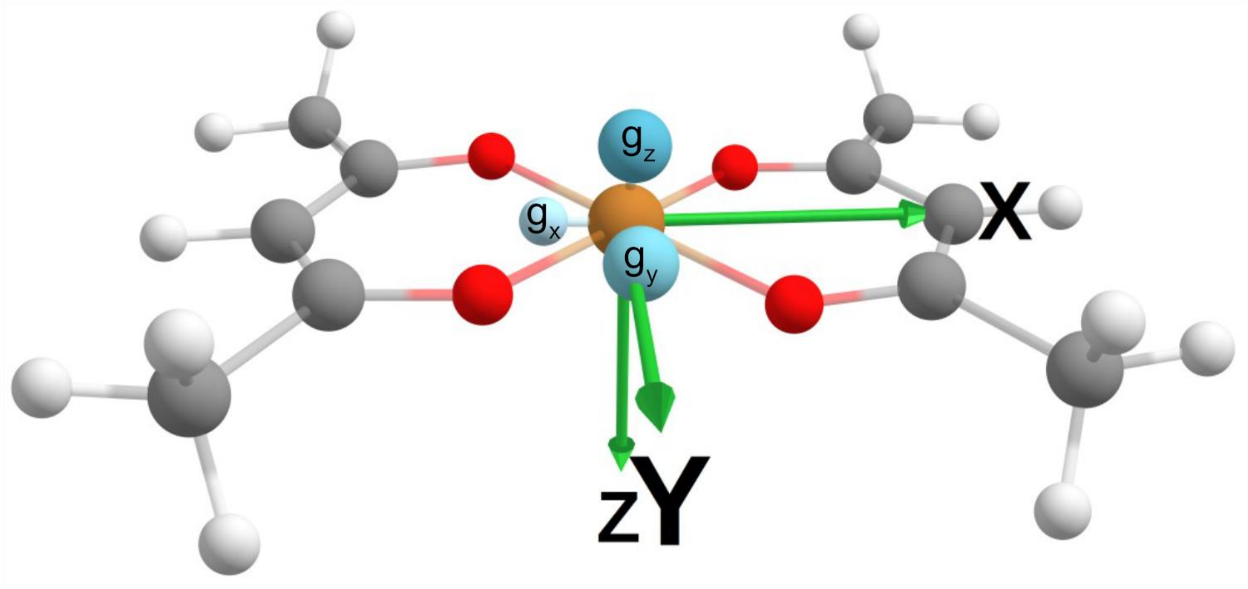

Figure S65: DFT-computed principal tensor axes of the *g*-tensor (spheres), compared to the molecular coordinate frame (arrows).

The calculated g-tensor aligns with the molecular coordinate frame, and indicates that the smallest g-value is aligned along the molecular x-axis. Rotation around the [1 0 -1] crystal direction iteratively aligns B_0 with the molecular x and z axes (**Figures S46-S47**). Therefore, we expect the *g*-value to reach potential

maximum and minimum values of 2.261 and 2.048 over the course of the crystal rotation. We accomplish this by setting g_x = 2.048, g_z = 2.261, and rotating the molecule in EasySpin about the y axis, which is the second Euler angle.

13. Tabulation of powder T_1 anisotropy data

The following data are used to form the powder T_1 anisotropy series analyzed in the main text. All $Cu(acac)_2$ data were collected at a microwave frequency of 9.7060 GHz, while all CuOEP data were collected at 9.6291 GHz.

Table S9: 10 K saturation recovery T_1 anisotropy data for 0.1% $Cu(acac)_2$ in $Pd(acac)_2$ (powder polycrystalline sample)

B (G)	T_1 (ns)	% uncertainty	β (stretching factor)
2805.0	9.805E+06	3.4	0.747
2831.9	9.670E+06	4.5	0.762
2858.8	9.765E+06	4.2	0.762
2885.6	9.782E+06	4.4	0.762
2912.5	9.616E+06	3.0	0.730
2939.4	9.394E+06	3.9	0.699
2966.3	9.117E+06	3.8	0.697
2993.1	1.025E+07	2.6	0.765
3020.0	1.050E+07	2.9	0.764
3046.9	1.060E+07	3.1	0.758
3073.8	1.054E+07	3.2	0.750
3100.6	1.050E+07	3.4	0.744
3127.5	1.041E+07	3.4	0.735
3154.4	1.043E+07	2.8	0.757
3181.3	1.035E+07	2.4	0.779
3208.1	1.032E+07	2.4	0.776
3235.0	1.017E+07	2.2	0.767
3261.9	1.011E+07	1.9	0.767
3288.8	9.967E+06	1.7	0.766
3315.6	9.966E+06	1.7	0.782
3342.5	9.648E+06	1.5	0.792
3369.4	9.451E+06	1.5	0.768
3376.0	9.928E+06	1.5	0.811
3383.0	1.044E+07	1.1	0.865
3396.3	9.040E+06	1.6	0.730
3410.0	9.816E+06	1.9	0.773
3423.1	1.222E+07	1.2	0.876
3437.0	1.318E+07	1.1	0.901
3450.0	1.303E+07	0.8	0.906

Table S10: 15 K saturation recovery T_1 anisotropy data for 0.1% $Cu(acac)_2$ in $Pd(acac)_2$ (powder polycrystalline sample)

B (G)	T_1 (ns)	% uncertainty	β (stretching factor)
2805.0	9.779E+05	2.3	0.859
2818.2	9.805E+05	2.7	0.867
2831.3	9.768E+05	2.0	0.860
2844.5	9.629E+05	2.2	0.862
2857.7	9.736E+05	2.0	0.842
2870.8	9.791E+05	2.1	0.842
2884.0	9.697E+05	2.2	0.841

2897.1	9.723E+05	1.8	0.852
2910.3	9.682E+05	2.0	0.823
2923.5	9.473E+05	1.8	0.827
2936.6	9.504E+05	1.9	0.797
2949.8	9.391E+05	1.5	0.799
2963.0	9.451E+05	1.9	0.793
2976.1	9.903E+05	1.4	0.849
2989.3	1.017E+06	1.4	0.860
3002.4	1.020E+06	1.4	0.860
3015.6	1.027E+06	1.3	0.864
3028.8	1.034E+06	1.4	0.855
3041.9	1.029E+06	1.5	0.861
3055.1	1.037E+06	1.5	0.848
3068.3	1.017E+06	1.2	0.855
3081.4	1.023E+06	1.4	0.848
3094.6	1.023E+06	1.3	0.848
3107.8	1.022E+06	1.3	0.846
3120.9	1.014E+06	1.4	0.839
3134.1	1.012E+06	1.5	0.839
3147.2	1.007E+06	1.4	0.841
3160.4	1.031E+06	1.3	0.866
3173.6	1.026E+06	1.2	0.875
3186.7	1.027E+06	1.3	0.871
3199.9	1.023E+06	1.1	0.871
3213.1	1.024E+06	1.2	0.872
3226.2	1.018E+06	1.2	0.868
3239.4	1.021E+06	1.2	0.865
3252.6	1.016E+06	1.2	0.863
3265.7	1.020E+06	1.2	0.860
3278.9	1.021E+06	1.1	0.861
3292.0	1.022E+06	1.2	0.862
3305.2	1.025E+06	1.2	0.867
3318.4	1.039E+06	1.2	0.874
3331.5	1.061E+06	1.2	0.888
3344.7	1.026E+06	1.3	0.877
3357.9	1.057E+06	1.2	0.901
3371.0	1.015E+06	1.4	0.866
3384.2	1.121E+06	1.2	0.924
3397.3	9.154E+05	0.8	0.831
3410.5	9.924E+05	1.5	0.862
3423.7	1.150E+06	1.2	0.922
3436.8	1.216E+06	1.1	0.940
3450.0	1.151E+06	0.8	0.945

**Table S11**: 20 K saturation recovery T_1 anisotropy data for 0.1% Cu(acac)$_2$ in Pd(acac)$_2$ (powder polycrystalline sample)			
B (G)	T_1 (ns)	% uncertainty	β (stretching factor)
2805.0	2.428E+05	1.80	0.920

2818.2	2.434E+05	1.34	0.914
2831.3	2.448E+05	1.52	0.921
2844.5	2.437E+05	1.22	0.924
2857.7	2.437E+05	1.68	0.912
2870.8	2.436E+05	1.22	0.904
2884.0	2.444E+05	1.26	0.903
2897.1	2.407E+05	1.54	0.904
2910.3	2.378E+05	1.26	0.907
2923.5	2.365E+05	1.44	0.901
2936.6	2.339E+05	1.26	0.901
2949.8	2.315E+05	1.34	0.888
2963.0	2.259E+05	1.21	0.890
2976.1	2.433E+05	0.97	0.917
2989.3	2.456E+05	0.90	0.926
3002.4	2.467E+05	0.81	0.925
3015.6	2.455E+05	0.88	0.924
3028.8	2.448E+05	0.99	0.924
3041.9	2.441E+05	1.06	0.920
3055.1	2.435E+05	0.99	0.923
3068.3	2.417E+05	1.00	0.918
3081.4	2.405E+05	0.74	0.913
3094.6	2.392E+05	0.90	0.915
3107.8	2.374E+05	0.81	0.918
3120.9	2.354E+05	0.99	0.911
3134.1	2.337E+05	0.97	0.914
3147.2	2.317E+05	0.93	0.916
3160.4	2.390E+05	0.94	0.932
3173.6	2.384E+05	0.88	0.933
3186.7	2.376E+05	0.93	0.932
3199.9	2.368E+05	0.87	0.930
3213.1	2.357E+05	0.92	0.928
3226.2	2.345E+05	0.93	0.927
3239.4	2.339E+05	0.93	0.927
3252.6	2.334E+05	0.88	0.926
3265.7	2.327E+05	0.93	0.926
3278.9	2.317E+05	0.89	0.925
3292.0	2.306E+05	0.95	0.928
3305.2	2.308E+05	0.91	0.931
3318.4	2.310E+05	0.92	0.938
3331.5	2.346E+05	0.82	0.946
3344.7	2.326E+05	0.84	0.939
3357.9	2.358E+05	0.92	0.952
3371.0	2.330E+05	1.05	0.930
3384.2	2.464E+05	0.82	0.964
3397.3	2.247E+05	0.88	0.902
3410.5	2.290E+05	1.25	0.927
3423.7	2.479E+05	0.86	0.963
3436.8	2.603E+05	0.79	0.973
3450.0	2.461E+05	0.55	0.973

**Table S12**: 25 K saturation recovery T_1 anisotropy data for 0.1% $Cu(acac)_2$ in $Pd(acac)_2$ (powder polycrystalline sample)			
B (G)	T_1 (ns)	% uncertainty	β (stretching factor)
2805.0	9.960E+04	1.29	0.953
2818.2	9.955E+04	1.67	0.951
2831.3	9.827E+04	1.32	0.956
2844.5	9.801E+04	1.54	0.941
2857.7	9.703E+04	1.68	0.946
2870.8	9.775E+04	1.70	0.942
2884.0	9.666E+04	1.19	0.946
2897.1	9.691E+04	1.41	0.943
2910.3	9.432E+04	1.43	0.946
2923.5	9.312E+04	1.64	0.937
2936.6	9.294E+04	1.56	0.935
2949.8	9.212E+04	1.09	0.929
2963.0	9.090E+04	1.02	0.921
2976.1	9.696E+04	1.08	0.950
2989.3	9.767E+04	0.90	0.959
3002.4	9.707E+04	0.88	0.953
3015.6	9.648E+04	1.02	0.956
3028.8	9.611E+04	1.00	0.959
3041.9	9.523E+04	1.00	0.956
3055.1	9.484E+04	0.92	0.959
3068.3	9.428E+04	0.98	0.952
3081.4	9.316E+04	1.15	0.950
3094.6	9.304E+04	0.87	0.952
3107.8	9.170E+04	0.81	0.950
3120.9	9.085E+04	0.96	0.946
3134.1	8.955E+04	1.10	0.950
3147.2	8.917E+04	0.92	0.950
3160.4	9.295E+04	0.77	0.964
3173.6	9.244E+04	0.89	0.965
3186.7	9.157E+04	0.79	0.966
3199.9	9.046E+04	0.79	0.966
3213.1	8.965E+04	0.79	0.966
3226.2	8.883E+04	0.79	0.966
3239.4	8.823E+04	0.77	0.963
3252.6	8.728E+04	0.77	0.965
3265.7	8.676E+04	0.79	0.964
3278.9	8.610E+04	0.91	0.964
3292.0	8.551E+04	0.81	0.964
3305.2	8.529E+04	0.90	0.966
3318.4	8.471E+04	0.92	0.970
3331.5	8.575E+04	0.84	0.976
3344.7	8.649E+04	0.86	0.971
3357.9	8.672E+04	0.85	0.979
3371.0	8.767E+04	1.03	0.963

3384.2	8.945E+04	0.79	0.987
3397.3	8.961E+04	0.66	0.932
3410.5	8.628E+04	1.21	0.967
3423.7	8.953E+04	0.89	0.985
3436.8	9.320E+04	0.76	0.993
3450.0	8.874E+04	0.45	0.989

Table S13: 30 K saturation recovery T_1 anisotropy data for 0.1% $Cu(acac)_2$ in $Pd(acac)_2$ (powder polycrystalline sample)

B (G)	T_1 (ns)	% uncertainty	β (stretching factor)
2805.0	5.073E+04	2.01	0.954
2818.2	4.967E+04	1.49	0.951
2831.3	5.018E+04	1.67	0.956
2844.5	4.948E+04	2.12	0.966
2857.7	5.004E+04	1.75	0.970
2870.8	4.851E+04	2.16	0.945
2884.0	4.809E+04	1.52	0.957
2897.1	4.766E+04	2.07	0.969
2910.3	4.677E+04	1.93	0.957
2923.5	4.690E+04	2.13	0.947
2936.6	4.546E+04	1.76	0.958
2949.8	4.473E+04	1.70	0.946
2963.0	4.438E+04	1.81	0.944
2976.1	4.728E+04	1.47	0.965
2989.3	4.781E+04	1.68	0.968
3002.4	4.740E+04	1.39	0.971
3015.6	4.717E+04	1.45	0.972
3028.8	4.674E+04	1.28	0.969
3041.9	4.657E+04	1.46	0.973
3055.1	4.580E+04	1.35	0.965
3068.3	4.527E+04	1.66	0.971
3081.4	4.453E+04	1.57	0.973
3094.6	4.419E+04	1.60	0.977
3107.8	4.383E+04	1.56	0.978
3120.9	4.330E+04	1.45	0.969
3134.1	4.281E+04	1.57	0.964
3147.2	4.228E+04	1.66	0.971
3160.4	4.443E+04	1.44	0.981
3173.6	4.429E+04	1.49	0.982
3186.7	4.362E+04	1.48	0.986
3199.9	4.295E+04	1.60	0.983
3213.1	4.250E+04	1.51	0.984
3226.2	4.183E+04	1.55	0.983
3239.4	4.135E+04	1.54	0.985
3252.6	4.069E+04	1.58	0.985
3265.7	4.029E+04	1.69	0.986
3278.9	3.963E+04	1.68	0.986
3292.0	3.927E+04	1.72	0.986

3305.2	3.895E+04	1.79	0.987
3318.4	3.869E+04	1.91	0.989
3331.5	3.907E+04	1.84	0.991
3344.7	3.975E+04	1.86	0.986
3357.9	3.968E+04	1.84	0.992
3371.0	4.070E+04	1.95	0.984
3384.2	4.042E+04	1.70	1.000
3397.3	4.306E+04	0.85	0.943
3410.5	4.022E+04	2.28	0.993
3423.7	4.057E+04	1.88	1.000
3436.8	4.186E+04	1.74	1.006
3450.0	4.016E+04	1.31	0.994

Table S14: 40 K saturation recovery T_1 anisotropy data for 0.1% Cu(acac)$_2$ in Pd(acac)$_2$ (powder polycrystalline sample)

B (G)	T_1 (ns)	% uncertainty	β (stretching factor)
2805.0	2.019E+04	1.69	0.933
2831.9	1.950E+04	1.75	0.929
2858.8	1.930E+04	1.54	0.935
2885.6	1.840E+04	1.61	0.926
2912.5	1.778E+04	1.52	0.924
2939.4	1.717E+04	1.77	0.929
2966.3	1.661E+04	1.43	0.914
2993.1	1.809E+04	0.71	0.936
3020.0	1.751E+04	1.06	0.933
3046.9	1.696E+04	0.89	0.936
3073.8	1.636E+04	0.91	0.930
3100.6	1.579E+04	1.06	0.935
3127.5	1.528E+04	0.78	0.929
3154.4	1.555E+04	1.05	0.929
3181.3	1.593E+04	1.00	0.933
3208.1	1.535E+04	0.96	0.937
3235.0	1.480E+04	0.88	0.940
3261.9	1.431E+04	0.88	0.941
3288.8	1.378E+04	0.85	0.947
3315.6	1.342E+04	0.76	0.950
3342.5	1.401E+04	0.81	0.946
3369.4	1.472E+04	0.92	0.938
3376.0	1.438E+04	0.67	0.957
3383.0	1.400E+04	0.90	0.960
3396.3	1.556E+04	2.12	0.893
3410.0	1.469E+04	0.74	0.946
3423.1	1.399E+04	0.69	0.961
3437.0	1.432E+04	0.87	0.967
3450.0	1.374E+04	1.35	0.947

**Table S15**: 60 K saturation recovery T_1 anisotropy data for 0.1% $Cu(acac)_2$ in $Pd(acac)_2$ (powder polycrystalline sample)			
B (G)	T_1 (ns)	% uncertainty	β (stretching factor)
2805.0	6109	2.49	0.925
2831.9	5804	1.90	0.970
2858.8	5702	2.01	0.970
2885.6	5323	2.66	0.948
2912.5	5015	1.66	0.926
2939.4	4735	1.73	0.959
2966.3	4516	1.87	0.944
2993.1	5081	1.61	0.940
3020.0	4862	1.53	0.932
3046.9	4571	1.24	0.940
3073.8	4329	1.41	0.936
3100.6	4110	1.17	0.953
3127.5	3924	1.66	0.947
3154.4	4044	1.58	0.940
3181.3	4184	1.37	0.937
3208.1	3964	1.56	0.942
3235.0	3758	1.12	0.954
3261.9	3570	0.96	0.954
3288.8	3411	1.04	0.966
3315.6	3282	0.92	0.974
3342.5	3513	1.21	0.952
3369.4	3742	1.16	0.937
3376.0	3621	0.95	0.966
3383.0	3427	0.94	0.979
3396.3	3916	3.64	0.889
3410.0	3797	0.89	0.959
3423.1	3456	0.83	0.979
3437.0	3462	0.95	0.991
3450.0	3296	2.03	0.975

**Table S16**: 100 K saturation recovery T_1 anisotropy data for 0.1% $Cu(acac)_2$ in $Pd(acac)_2$ (powder polycrystalline sample)			
B (G)	T_1 (ns)	% uncertainty	β (stretching factor)
2805.0	1729	5.5	1.013
2831.9	1656	3.2	1.071
2858.8	1504	3.4	1.054
2885.6	1405	6.4	0.984
2912.5	1280	6.3	0.978
2939.4	1202	4.1	0.947
2966.3	1206	8.0	0.945
2993.1	1365	3.4	0.969
3020.0	1268	2.1	0.981
3046.9	1171	2.7	0.974
3073.8	1086	3.6	0.945
3100.6	1006	3.8	0.936

3127.5	962	4.4	0.956
3154.4	1030	3.1	0.908
3181.3	1051	3.4	0.905
3208.1	957	3.8	0.904
3235.0	910	3.2	0.925
3261.9	857	3.0	0.926
3288.8	812	3.4	0.935
3315.6	771	3.7	0.926
3342.5	822	4.8	0.882
3369.4	910	3.8	0.885
3396.3	991	2.5	0.950
3423.1	818	3.6	0.922
3450.0	736	2.5	0.962

Table S17: 150 K inversion recovery T_1 anisotropy data for 0.1% $Cu(acac)_2$ in $Pd(acac)_2$ (powder polycrystalline sample)

B (G)	T_1 (ns)	% uncertainty	β (stretching factor)
2805.0	646	8.0	1.009
2831.9	594	8.2	0.982
2858.8	563	7.2	1.027
2885.6	510	7.6	1.004
2912.5	473	6.8	1.047
2939.4	433	7.2	1.016
2966.3	431	5.8	1.060
2993.1	466	3.4	0.958
3020.0	437	3.3	1.005
3046.9	408	3.2	1.007
3073.8	370	3.2	0.990
3100.6	352	3.1	1.009
3127.5	325	3.0	1.008
3154.4	343	2.5	0.921
3181.3	339	2.1	0.934
3208.1	313	2.0	0.947
3235.0	295	1.8	0.971
3261.9	279	1.8	0.994
3288.8	259	2.9	0.987
3315.6	252	2.3	1.024
3342.5	251	2.2	0.921
3369.4	269	2.6	0.923
3396.3	347	5.0	0.971
3423.1	260	1.5	1.008
3450.0	236	3.5	0.990

Table S18: 10 K saturation recovery T_1 anisotropy data for 1% CuOEP in ZnOEP (powder polycrystalline sample)

B (G)	T_1 (ns)	% uncertainty	β (stretching factor)
2830.0	1.076E+07	2.19	0.916

2853.4	1.044E+07	1.57	0.938
2876.8	1.081E+07	1.21	0.926
2900.3	1.085E+07	1.09	0.922
2923.7	1.114E+07	1.27	0.911
2947.1	1.132E+07	1.06	0.914
2970.6	1.142E+07	1.22	0.911
2994.0	1.154E+07	1.23	0.907
3017.5	1.141E+07	0.99	0.915
3041.0	1.138E+07	1.96	0.915
3064.5	1.126E+07	1.33	0.927
3087.9	1.154E+07	1.34	0.919
3111.4	1.141E+07	1.20	0.914
3134.8	1.146E+07	1.13	0.918
3158.3	1.131E+07	1.20	0.912
3181.7	1.122E+07	1.07	0.896
3205.2	1.089E+07	1.03	0.901
3228.6	1.042E+07	0.94	0.896
3252.1	9.853E+06	0.84	0.890
3275.5	9.356E+06	0.88	0.884
3299.0	8.984E+06	0.78	0.866
3322.4	8.608E+06	0.79	0.849
3345.9	8.266E+06	0.98	0.832
3369.3	8.029E+06	1.10	0.832
3392.8	8.479E+06	1.15	0.846
3416.2	1.011E+07	1.07	0.877
3439.7	1.188E+07	0.63	0.907
3463.1	1.204E+07	0.71	0.933
3486.6	1.251E+07	0.70	0.937
3510.0	1.278E+07	2.07	0.909

**Table S19**: 15 K saturation recovery T_1 anisotropy data for 1% CuOEP in ZnOEP (powder polycrystalline sample)			
B (G)	T_1 (ns)	% uncertainty	β (stretching factor)
2830.0	2.830E+06	2.64	0.933
2853.4	2.896E+06	1.47	0.941
2876.9	3.010E+06	1.58	0.915
2900.3	3.047E+06	1.31	0.917
2923.8	3.075E+06	1.31	0.922
2947.2	3.131E+06	1.35	0.922
2970.7	3.213E+06	1.44	0.913
2994.1	3.230E+06	1.17	0.919
3017.6	3.214E+06	0.97	0.933
3041.0	3.177E+06	0.83	0.935
3064.5	3.163E+06	0.81	0.938
3087.9	3.206E+06	0.96	0.935
3111.4	3.256E+06	0.90	0.931
3134.8	3.322E+06	0.84	0.926
3158.3	3.374E+06	0.92	0.924

3181.7	3.396E+06	0.89	0.923
3205.2	3.372E+06	0.80	0.923
3228.6	3.303E+06	0.74	0.925
3252.1	3.218E+06	0.83	0.927
3275.5	3.143E+06	0.85	0.931
3299.0	3.096E+06	1.02	0.926
3322.4	3.044E+06	1.01	0.919
3345.9	2.986E+06	1.07	0.917
3369.3	2.993E+06	1.30	0.920
3392.8	3.091E+06	1.24	0.928
3416.2	3.427E+06	1.17	0.927
3439.7	3.933E+06	0.81	0.917
3463.1	3.596E+06	0.71	0.942
3486.6	3.589E+06	0.66	0.947
3510.0	3.618E+06	1.05	0.934

**Table S20**: 20 K saturation recovery T_1 anisotropy data for 1% CuOEP in ZnOEP (powder polycrystalline sample)			
B (G)	T_1 (ns)	% uncertainty	β (stretching factor)
2830.0	1.190E+06	3.14	0.939
2853.4	1.235E+06	2.11	0.935
2876.9	1.227E+06	1.56	0.951
2900.3	1.241E+06	1.34	0.952
2923.8	1.265E+06	1.67	0.945
2947.2	1.280E+06	1.49	0.951
2970.7	1.315E+06	1.33	0.955
2994.1	1.332E+06	1.14	0.950
3017.6	1.336E+06	1.26	0.951
3041.0	1.330E+06	0.99	0.961
3064.5	1.339E+06	0.91	0.964
3087.9	1.345E+06	0.75	0.965
3111.4	1.359E+06	0.94	0.962
3134.8	1.372E+06	0.81	0.963
3158.3	1.386E+06	0.93	0.962
3181.7	1.401E+06	0.75	0.956
3205.2	1.398E+06	0.74	0.953
3228.6	1.394E+06	0.64	0.952
3252.1	1.372E+06	0.62	0.953
3275.5	1.356E+06	0.64	0.955
3299.0	1.343E+06	0.68	0.955
3322.4	1.331E+06	0.75	0.950
3345.9	1.319E+06	0.74	0.949
3369.3	1.333E+06	0.92	0.948
3392.8	1.357E+06	0.82	0.951
3404.0	1.406E+06	0.98	0.952
3416.2	1.462E+06	1.01	0.945
3421.0	1.527E+06	1.10	0.928
3427.0	1.704E+06	1.19	0.903

3439.7	1.661E+06	1.04	0.918
3451.0	1.501E+06	0.89	0.951
3463.1	1.478E+06	0.79	0.963
3486.6	1.469E+06	0.67	0.966
3510.0	1.478E+06	1.02	0.950

Table S21: 30 K saturation recovery T_1 anisotropy data for 1% CuOEP in ZnOEP (powder polycrystalline sample)

B (G)	T_1 (ns)	% uncertainty	β (stretching factor)
2830.0	4.101E+05	2.07	0.954
2853.4	4.139E+05	1.51	0.974
2876.9	4.169E+05	1.48	0.976
2900.3	4.135E+05	1.16	0.968
2923.8	4.113E+05	1.45	0.962
2947.2	4.129E+05	1.34	0.977
2970.7	4.163E+05	0.89	0.961
2994.1	4.160E+05	1.33	0.961
3017.6	4.181E+05	0.94	0.984
3041.0	4.218E+05	0.98	0.974
3064.5	4.215E+05	0.88	0.979
3087.9	4.170E+05	0.81	0.979
3111.4	4.172E+05	0.94	0.979
3134.8	4.174E+05	0.81	0.981
3158.3	4.161E+05	0.88	0.975
3181.7	4.151E+05	0.82	0.975
3205.2	4.142E+05	0.82	0.971
3228.6	4.157E+05	0.88	0.969
3252.1	4.129E+05	0.83	0.969
3275.5	4.100E+05	0.80	0.970
3299.0	4.061E+05	0.83	0.968
3322.4	4.026E+05	0.81	0.966
3345.9	3.998E+05	0.75	0.967
3369.3	3.991E+05	0.93	0.967
3392.8	4.034E+05	0.77	0.963
3416.2	4.264E+05	0.84	0.958
3439.7	4.856E+05	1.53	0.914
3463.1	4.347E+05	0.81	0.977
3486.6	4.335E+05	0.75	0.981
3510.0	4.314E+05	0.96	0.974

Table S22: 35 K saturation recovery T_1 anisotropy data for 1% CuOEP in ZnOEP (powder polycrystalline sample)

B (G)	T_1 (ns)	% uncertainty	β (stretching factor)
2830.0	2.596E+05	1.95	0.964
2853.4	2.616E+05	1.45	0.973
2876.9	2.625E+05	1.28	0.972
2900.3	2.595E+05	1.04	0.967

2923.8	2.570E+05	1.10	0.975
2947.2	2.560E+05	1.33	0.971
2970.7	2.538E+05	1.32	0.971
2994.1	2.541E+05	1.29	0.969
3017.6	2.547E+05	1.21	0.970
3041.0	2.550E+05	1.08	0.970
3064.5	2.560E+05	1.04	0.969
3087.9	2.544E+05	1.15	0.968
3111.4	2.520E+05	1.07	0.968
3134.8	2.499E+05	1.23	0.968
3158.3	2.481E+05	1.16	0.966
3181.7	2.467E+05	1.05	0.965
3205.2	2.463E+05	1.05	0.967
3228.6	2.466E+05	1.08	0.968
3252.1	2.462E+05	1.11	0.970
3275.5	2.436E+05	1.13	0.970
3299.0	2.398E+05	1.07	0.969
3322.4	2.374E+05	1.06	0.968
3345.9	2.354E+05	1.04	0.969
3369.3	2.329E+05	1.13	0.969
3392.8	2.355E+05	1.00	0.964
3416.2	2.473E+05	1.02	0.962
3439.7	2.794E+05	1.55	0.922
3463.1	2.570E+05	1.06	0.976
3486.6	2.582E+05	0.99	0.976
3510.0	2.506E+05	1.37	0.968

Table S23: 40 K saturation recovery T_1 anisotropy data for 1% CuOEP in ZnOEP (powder polycrystalline sample)

B (G)	T_1 (ns)	% uncertainty	β (stretching factor)
2830.0	1.783E+05	1.88	0.984
2853.4	1.759E+05	1.46	0.982
2876.9	1.757E+05	1.26	0.978
2900.3	1.726E+05	1.54	0.978
2923.8	1.706E+05	1.30	0.975
2947.2	1.685E+05	1.28	0.973
2970.7	1.666E+05	1.17	0.973
2994.1	1.646E+05	1.48	0.970
3017.6	1.671E+05	1.20	0.976
3041.0	1.671E+05	1.04	0.976
3064.5	1.665E+05	1.03	0.975
3087.9	1.639E+05	1.07	0.974
3111.4	1.613E+05	1.04	0.976
3134.8	1.592E+05	1.21	0.977
3158.3	1.562E+05	1.16	0.977
3181.7	1.537E+05	1.15	0.975
3205.2	1.540E+05	1.08	0.973
3228.6	1.550E+05	1.04	0.975

3252.1	1.549E+05	1.04	0.975
3275.5	1.529E+05	0.99	0.976
3299.0	1.495E+05	1.04	0.978
3322.4	1.477E+05	1.07	0.977
3345.9	1.457E+05	1.05	0.978
3369.3	1.435E+05	1.15	0.979
3392.8	1.451E+05	1.07	0.973
3416.2	1.529E+05	1.08	0.969
3439.7	1.735E+05	1.75	0.928
3463.1	1.611E+05	1.18	0.981
3486.6	1.623E+05	1.01	0.980
3510.0	1.566E+05	1.20	0.984

Table S24: 60 K saturation recovery T_1 anisotropy data for 1% CuOEP in ZnOEP (powder polycrystalline sample)

B (G)	T_1 (ns)	% uncertainty	β (stretching factor)
2830.0	4.304E+04	1.74	0.988
2853.4	4.313E+04	0.72	0.980
2876.9	4.277E+04	1.03	0.972
2900.3	4.043E+04	0.84	0.987
2923.8	3.911E+04	0.62	0.975
2947.2	3.798E+04	0.77	0.981
2970.7	3.662E+04	0.74	0.985
2994.1	3.615E+04	0.69	0.977
3017.6	3.643E+04	0.70	0.966
3041.0	3.665E+04	0.57	0.975
3064.5	3.627E+04	0.42	0.970
3087.9	3.523E+04	0.57	0.972
3111.4	3.412E+04	0.52	0.971
3134.8	3.292E+04	0.51	0.973
3158.3	3.190E+04	0.51	0.972
3181.7	3.100E+04	0.62	0.972
3205.2	3.088E+04	0.53	0.973
3228.6	3.132E+04	0.47	0.970
3252.1	3.143E+04	0.44	0.969
3275.5	3.068E+04	0.42	0.973
3299.0	2.970E+04	0.47	0.977
3322.4	2.906E+04	0.53	0.979
3345.9	2.841E+04	0.44	0.979
3369.3	2.774E+04	0.65	0.985
3392.8	2.820E+04	0.46	0.973
3416.2	2.998E+04	0.36	0.971
3439.7	3.337E+04	1.30	0.945
3463.1	3.249E+04	0.46	0.981
3486.6	3.283E+04	0.38	0.972
3510.0	3.138E+04	0.47	0.979

**Table S25**: 100 K saturation recovery T_1 anisotropy data for 1% CuOEP in ZnOEP (powder polycrystalline sample)			
B (G)	T_1 (ns)	% uncertainty	β (stretching factor)
2830.0	6619	5.30	0.904
2853.4	6622	3.48	0.965
2876.9	6345	2.35	0.980
2900.3	6092	3.19	0.991
2923.8	5838	2.99	0.956
2947.2	5486	2.56	0.960
2970.7	5297	2.22	0.980
2994.1	5134	2.23	0.977
3017.6	5343	1.52	0.986
3041.0	5391	1.15	0.985
3064.5	5365	1.25	0.970
3087.9	5136	1.04	0.977
3111.4	4903	1.18	0.984
3134.8	4668	1.07	0.997
3158.3	4449	1.47	0.997
3181.7	4270	1.44	1.007
3205.2	4275	1.36	0.994
3228.6	4370	1.18	0.986
3252.1	4376	1.45	0.979
3275.5	4231	1.50	0.987
3299.0	4071	1.56	0.993
3322.4	3937	1.26	1.004
3345.9	3808	1.35	1.003
3369.3	3727	2.03	1.011
3392.8	3779	1.30	1.005
3416.2	4114	1.63	0.983
3439.7	4554	1.92	0.953
3463.1	4473	1.85	0.992
3486.6	4466	1.29	0.995
3510.0	4255	2.48	1.017

**Table S26**: 150 K saturation recovery T_1 anisotropy data for 1% CuOEP in ZnOEP (powder polycrystalline sample)			
B (G)	T_1 (ns)	% uncertainty	β (stretching factor)
2830.0	2040	2.70	1.021
2853.4	2022	1.64	0.998
2876.9	1916	1.74	0.994
2900.3	1801	1.72	0.992
2923.8	1699	1.97	0.989
2947.2	1598	1.86	0.977
2970.7	1536	2.01	0.967
2994.1	1503	1.38	0.993
3017.6	1537	1.08	0.977
3041.0	1589	1.27	0.963
3064.5	1568	1.32	0.969

3087.9	1491	1.27	0.975
3111.4	1395	1.31	0.971
3134.8	1325	1.17	0.976
3158.3	1256	1.19	0.979
3181.7	1201	1.41	0.973
3205.2	1202	1.46	0.961
3228.6	1238	1.86	0.947
3252.1	1244	1.71	0.947
3275.5	1195	1.77	0.957
3299.0	1138	1.50	0.963
3322.4	1097	1.41	0.975
3345.9	1052	1.17	0.984
3369.3	1033	1.37	0.980
3392.8	1041	1.67	0.971
3416.2	1140	2.13	0.940
3439.7	1247	3.32	0.927
3463.1	1264	1.80	0.955
3486.6	1249	1.34	0.967
3510.0	1193	3.21	0.936

Table S27: 294 K inversion recovery T_1 anisotropy data for 1% CuOEP in ZnOEP (powder polycrystalline sample)

B (G)	T_1 (ns)	% uncertainty	β (stretching factor)
2830.0	418	22.5	1.14
2853.4	324	16.6	0.98
2876.9	285	20.5	0.77
2900.3	281	15.3	0.88
2923.8	253	14.4	0.93
2947.2	246	14.3	0.97
2970.7	267	11.5	1.09
2994.1	225	14.3	0.94
3017.6	216	12.1	0.87
3041.0	251	6.8	1.01
3064.5	240	6.0	0.98
3087.9	216	6.2	0.92
3111.4	198	6.1	0.94
3134.8	186	6.2	0.92
3158.3	177	5.6	0.96
3181.7	168	5.5	0.95
3205.2	167	4.6	0.95
3228.6	168	3.7	0.92
3252.1	166	3.2	0.90
3275.5	162	2.4	0.94
3299.0	154	2.2	0.95
3322.4	155	2.0	0.99
3345.9	147	1.7	0.98
3369.3	146	2.2	1.00
3392.8	146	2.7	0.98

3416.2	152	2.7	0.91
3439.7	169	2.4	0.87
3463.1	175	2.7	0.94
3486.6	179	4.4	0.95
3510.0	189	21.8	1.07

14. Tabulation of single-crystal T_1 anisotropy data

Whenever the spectrum is selective between ^{63}Cu and ^{65}Cu, all peaks in **Tables S28 – S30** are measured at peaks corresponding to ^{63}Cu.

Table S28: 100 K single-crystal T_1 anisotropy for 0.1% $Cu(acac)_2$ in $Pd(acac)_2$. Ω indicates sample rod angle in the laboratory frame. Δ indicates 95% confidence interval width. Relative site indexes the lower and higher field Cu contributions at each angle, while absolute site indicates the assignment of these to sites in the crystal lattice. θ gives the molecular frame angle to B_0 after phase unwrapping. Overlap denotes if two peaks occupy the same field position.

Ω (°)	B (G)	$1/T_1$ (μs^{-1})	Δ $1/T_1$ (μs^{-1})	Relative site	M_I	Overlap	Absolute site	θ (°)
105	2819	5.33E-01	3.4E-02	1	-1.5	0	2	168.0
105	2992	5.17E-01	3.3E-02	1	-0.5	0	2	168.0
105	3167	5.45E-01	2.2E-02	1	0.5	0	2	168.0
105	3272	1.42E+00	7.3E-02	2	-1.5	0	1	75.5
105	3330	1.37E+00	6.4E-02	2	-0.5	1	1	75.5
105	3341	5.91E-01	2.6E-02	1	1.5	1	2	168.0
105	3383	1.32E+00	2.7E-02	2	0.5	0	1	75.5
105	3432	1.36E+00	6.3E-02	2	1.5	0	1	75.5
145	2838	6.44E-01	3.3E-02	1	-1.5	0	2	199.0
145	3010	6.50E-01	2.4E-02	1	-0.5	0	2	199.0
145	3182	6.56E-01	3.2E-02	1	0.5	0	2	199.0
145	3221	1.45E+00	1.2E-01	2	-1.5	0	1	110.0
145	3305	1.48E+00	8.8E-02	2	-0.5	0	1	110.0
145	3353	6.81E-01	2.6E-02	1	1.5	0	2	199.0
145	3381	1.43E+00	4.7E-02	2	0.5	1	1	110.0
145	3449	1.46E+00	4.0E-02	2	1.5	0	1	110.0
15	2816	5.60E-01	3.9E-02	1	-1.5	0	1	-12.0
15	2992	5.74E-01	2.2E-02	1	-0.5	0	1	-12.0
15	3166	5.69E-01	2.0E-02	1	0.5	0	1	-12.0
15	3273	1.43E+00	9.0E-02	2	-1.5	0	2	76.5
15	3335	1.41E+00	8.5E-02	2	-0.5	1	2	76.5
15	3342	6.31E-01	3.3E-02	1	1.5	1	1	-12.0
15	3389	1.41E+00	1.1E-01	2	0.5	0	2	76.5
15	3440	1.36E+00	9.5E-02	2	1.5	0	2	76.5
165	2983	1.01E+00	7.6E-02	1	-1.5	0	1	137.0
165	3013	1.16E+00	1.5E-01	2	-1.5	0	2	226.5
165	3130	9.71E-01	5.9E-02	1	-0.5	0	1	137.0
165	3154	1.07E+00	4.0E-02	2	-0.5	0	2	226.5
165	3275	1.08E+00	5.0E-02	1	0.5	0	1	137.0
165	3292	1.15E+00	5.9E-02	2	0.5	0	2	226.5
165	3414	1.03E+00	7.5E-02	1	1.5	1	1	137.0
165	3424	1.11E+00	5.1E-02	2	1.5	1	2	226.5
180	2891	7.58E-01	5.7E-02	1	-1.5	0	1	150.0
180	3055	7.77E-01	4.3E-02	1	-0.5	0	1	150.0
180	3126	1.22E+00	9.7E-02	2	-1.5	0	2	239.5
180	3218	8.42E-01	6.3E-02	1	0.5	0	1	150.0
180	3240	1.34E+00	8.1E-02	2	-0.5	0	2	239.5
180	3347	1.30E+00	7.8E-02	2	0.5	0	2	239.5

180	3379	8.43E-01	3.4E-02	1	1.5	0	1	150.0
180	3448	1.32E+00	5.8E-02	2	1.5	0	2	239.5
45	2812	5.13E-01	2.1E-02	1	-1.5	0	1	10.0
45	2987	5.00E-01	2.4E-02	1	-0.5	0	1	10.0
45	3161	5.25E-01	1.5E-02	1	0.5	0	1	10.0
45	3288	1.42E+00	9.8E-02	2	-1.5	0	2	102.0
45	3338	9.75E-01	6.4E-02	2	-0.5	1	2	102.0
45	3386	1.39E+00	5.7E-02	2	0.5	0	2	102.0
45	3429	1.37E+00	5.1E-02	2	1.5	0	2	102.0
75	2926	7.19E-01	3.6E-02	1	-1.5	0	1	34.0
75	3082	8.45E-01	3.0E-02	1	-0.5	1	1	34.0
75	3204	1.08E+00	6.2E-02	2	-0.5	0	2	126.0
75	3235	7.91E-01	3.2E-02	1	0.5	0	1	34.0
75	3320	1.06E+00	5.4E-02	2	0.5	0	2	126.0
75	3384	8.01E-01	3.2E-02	1	1.5	0	1	34.0
75	3430	1.13E+00	4.3E-02	1	1.5	0	1	34.0
90	2948	7.59E-01	6.8E-02	1	-1.5	0	2	142.5
90	3056	1.02E+00	9.8E-02	2	-1.5	0	1	51.0
90	3100	7.68E-01	3.9E-02	1	-0.5	0	2	142.5
90	3184	1.04E+00	5.2E-02	2	-0.5	0	1	51.0
90	3249	8.39E-01	4.1E-02	1	0.5	0	2	142.5
90	3306	1.04E+00	6.8E-02	2	0.5	0	1	51.0
90	3393	8.26E-01	2.9E-02	1	1.5	0	2	142.5
90	3424	1.09E+00	5.3E-02	1	1.5	0	2	142.5
105	2819	5.33E-01	3.4E-02	1	-1.5	0	2	168.0
105	2992	5.17E-01	3.3E-02	1	-0.5	0	2	168.0
105	3167	5.45E-01	2.2E-02	1	0.5	0	2	168.0
105	3272	1.42E+00	7.3E-02	2	-1.5	0	1	75.5
105	3330	1.37E+00	6.4E-02	2	-0.5	1	1	75.5
105	3341	5.91E-01	2.6E-02	1	1.5	1	2	168.0
105	3383	1.32E+00	2.7E-02	2	0.5	0	1	75.5
105	3432	1.36E+00	6.3E-02	2	1.5	0	1	75.5
145	2838	6.44E-01	3.3E-02	1	-1.5	0	2	199.0
145	3010	6.50E-01	2.4E-02	1	-0.5	0	2	199.0
145	3182	6.56E-01	3.2E-02	1	0.5	0	2	199.0
145	3221	1.45E+00	1.2E-01	2	-1.5	0	1	110.0
145	3305	1.48E+00	8.8E-02	2	-0.5	0	1	110.0
145	3353	6.81E-01	2.6E-02	1	1.5	0	2	199.0
145	3381	1.43E+00	4.7E-02	2	0.5	1	1	110.0
145	3449	1.46E+00	4.0E-02	2	1.5	0	1	110.0

Table S29: 20 K single-crystal T_1 anisotropy for 0.1% $Cu(acac)_2$ in $Pd(acac)_2$. Ω indicates sample rod angle in the laboratory frame. Δ indicates 95% confidence interval width. Relative site indexes the lower and higher field Cu contributions at each angle, while absolute site indicates the assignment of these to sites in the crystal lattice. θ gives the molecular frame angle to B_0 after phase unwrapping. Overlap denotes if two peaks occupy the same field position.

**Ω (°)**	**B (G)**	**$1/T_1$ (μs^{-1})**	**Δ $1/T_1$ (μs^{-1})**	**Relative site**	**M_I**	**Overlap**	**Absolute site**	**θ (°)**

105	2824	3.07E-03	6.6E-05	1	-1.5	0	1	166.0
105	2998	3.11E-03	4.2E-05	1	-0.5	0	1	166.0
105	3171	3.15E-03	4.8E-05	1	0.5	0	1	166.0
105	3255	3.78E-03	6.0E-05	2	-1.5	0	2	254.0
105	3322	3.90E-03	4.9E-05	2	-0.5	0	2	254.0
105	3344	3.31E-03	4.4E-05	1	0.5	0	1	166.0
105	3382	3.79E-03	3.8E-05	2	0.5	0	2	254.0
105	3435	3.75E-03	5.0E-05	2	1.5	0	2	254.0
135	2807	3.47E-03	5.4E-05	1	-1.5	0	1	188.0
135	2984	3.56E-03	5.6E-05	1	-0.5	0	1	188.0
135	3161	3.56E-03	3.2E-05	1	0.5	0	1	188.0
135	3298	4.63E-03	6.2E-05	2	-1.5	0	2	280.0
135	3338	3.90E-03	5.7E-05	1	1.5	1	1	188.0
135	3348	4.54E-03	4.5E-05	2	-0.5	1	2	280.0
135	3391	4.32E-03	4.3E-05	2	0.5	0	2	280.0
135	3434	4.01E-03	4.0E-05	2	1.5	0	2	280.0
15	2823	3.66E-03	6.8E-05	1	-1.5	0	2	165.0
15	2998	3.80E-03	5.7E-05	1	-0.5	0	2	165.0
15	3173	4.05E-03	6.1E-05	1	0.5	0	2	165.0
15	3247	4.72E-03	8.9E-05	2	-1.5	0	1	73.0
15	3321	4.66E-03	6.1E-05	2	-0.5	0	1	73.0
15	3347	4.40E-03	7.1E-05	1	1.5	0	2	165.0
15	3387	4.39E-03	5.2E-05	2	0.5	0	1	73.0
15	3445	4.29E-03	7.2E-05	2	1.5	0	1	73.0
165	2964	4.21E-03	7.8E-05	1	-1.5	0	1	220.5
165	3032	4.44E-03	1.1E-04	2	-1.5	0	2	311.5
165	3116	4.28E-03	7.3E-05	1	-0.5	0	1	220.5
165	3169	4.42E-03	9.5E-05	2	-0.5	0	2	311.5
165	3265	4.38E-03	6.9E-05	1	0.5	0	1	220.5
165	3303	4.52E-03	7.8E-05	2	0.5	0	2	311.5
165	3410	4.44E-03	6.0E-05	1	1.5	0	1	220.5
165	3432	4.37E-03	4.3E-05	2	1.5	1	2	311.5
45	2801	3.24E-03	5.2E-05	1	-1.5	0	2	182.0
45	2978	3.26E-03	4.9E-05	1	-0.5	0	2	182.0
45	3156	3.26E-03	3.7E-05	1	0.5	0	2	182.0
45	3323	4.11E-03	4.3E-05	2	-1.5	1	1	94.0
45	3334	3.49E-03	4.1E-05	1	1.5	1	2	182.0
45	3355	3.96E-03	4.2E-05	2	-0.5	0	1	94.0
45	3386	4.06E-03	6.4E-05	2	0.5	0	1	94.0
45	3421	3.93E-03	5.6E-05	2	1.5	0	1	94.0
75	2906	3.06E-03	7.2E-05	1	-1.5	0	2	211.5
75	3066	3.06E-03	5.3E-05	1	-0.5	0	2	211.5
75	3109	3.33E-03	7.9E-05	2	-1.5	0	1	123.0
75	3223	3.16E-03	4.1E-05	1	0.5	1	2	211.5
75	3333	3.49E-03	4.1E-05	2	0.5	0	1	123.0
75	3379	3.21E-03	4.6E-05	1	1.5	0	2	211.5
75	3435	3.37E-03	3.8E-05	2	1.5	0	1	123.0
90	2934	2.99E-03	1.0E-04	1	-1.5	0	1	144.0
90	3071	3.24E-03	8.3E-05	2	-1.5	1	2	233.0

90	3089	3.12E-03	5.8E-05	1	-0.5	1	1	144.0
90	3196	3.26E-03	6.6E-05	2	-0.5	0	2	233.0
90	3241	3.11E-03	4.0E-05	1	0.5	0	1	144.0
90	3316	3.48E-03	5.5E-05	2	0.5	0	2	233.0
90	3389	3.18E-03	4.5E-05	1	1.5	0	1	144.0
90	3429	3.33E-03	2.8E-05	2	1.5	0	2	233.0
0	2928	4.45E-03	9.8E-05	1	-1.5	0	2	145.0
0	3081	4.72E-03	6.0E-05	2	-1.5	1	1	55.0
0	3088	4.67E-03	5.2E-05	1	-0.5	1	2	145.0
0	3207	4.93E-03	6.6E-05	2	-0.5	0	1	55.0
0	3244	4.76E-03	6.1E-05	1	0.5	0	2	145.0
0	3332	5.03E-03	5.6E-05	2	0.5	0	1	55.0
0	3399	4.77E-03	5.4E-05	1	1.5	0	2	145.0
0	3446	4.80E-03	5.3E-05	2	1.5	0	1	55.0
-225	2818	3.90E-03	6.2E-05	1	-1.5	0	1	-167.0
-225	2995	3.90E-03	5.4E-05	1	-0.5	0	1	-167.0
-225	3171	4.00E-03	6.3E-05	1	0.5	0	1	-167.0
-225	3270	4.93E-03	1.2E-04	2	-1.5	0	2	-76.0
-225	3334	4.88E-03	6.0E-05	2	-0.5	0	2	-76.0
-225	3346	4.22E-03	5.8E-05	1	1.5	1	1	-167.0
-225	3393	4.54E-03	3.2E-05	2	0.5	0	2	-76.0
-225	3446	4.35E-03	5.0E-05	2	1.5	0	2	-76.0
-195	2933	4.41E-03	1.0E-04	1	-1.5	0	1	-143.5
-195	3074	4.78E-03	1.1E-04	2	-1.5	1	2	-54.0
-195	3091	4.51E-03	6.0E-05	1	-0.5	1	1	-143.5
-195	3202	4.85E-03	8.8E-05	2	-0.5	0	2	-54.0
-195	3249	4.61E-03	8.3E-05	1	0.5	0	1	-143.5
-195	3327	4.94E-03	6.4E-05	2	0.5	0	2	-54.0
-195	3401	4.67E-03	6.5E-05	1	1.5	0	1	-143.5
-195	3444	4.71E-03	6.7E-05	2	1.5	0	2	-54.0
-180	2934	4.47E-03	7.5E-05	1	-1.5	0	2	-36.0
-180	3073	4.77E-03	7.2E-05	2	-1.5	1	1	-126.0
-180	3091	4.54E-03	7.0E-05	1	-0.5	1	2	-36.0
-180	3203	4.77E-03	5.0E-05	2	-0.5	0	1	-126.0
-180	3248	4.61E-03	6.5E-05	1	0.5	0	2	-36.0
-180	3327	4.91E-03	4.7E-05	2	0.5	0	1	-126.0
-180	3400	4.70E-03	5.1E-05	1	1.5	0	2	-36.0
-180	3444	4.67E-03	4.3E-05	2	1.5	0	1	-126.0
-105	2916	3.20E-03	4.0E-05	1	-1.5	0	2	33.0
-105	3074	3.23E-03	2.6E-05	1	-0.5	0	2	33.0
-105	3105	3.42E-03	3.8E-05	2	-1.5	0	1	-56.5
-105	3221	3.48E-03	4.8E-05	2	-0.5	0	1	-56.5
-105	3231	3.32E-03	4.8E-05	1	0.5	0	2	33.0
-105	3333	3.56E-03	3.7E-05	2	0.5	0	1	-56.5
-105	3384	3.39E-03	3.4E-05	1	1.5	0	2	33.0
-105	3437	3.43E-03	3.1E-05	2	1.5	0	1	-56.5
-135	2804	3.45E-03	4.3E-05	1	-1.5	0	2	5.0
-135	2982	3.42E-03	3.1E-05	1	-0.5	0	2	5.0
-135	3159	3.48E-03	3.0E-05	1	0.5	0	2	5.0

-135	3329	4.20E-03	3.4E-05	2	-1.5	1	1	-86.0
-135	3337	3.69E-03	3.1E-05	1	1.5	1	2	5.0
-135	3359	4.15E-03	2.6E-05	2	-0.5	1	1	-86.0
-135	3389	4.06E-03	3.2E-05	2	0.5	0	1	-86.0
-135	3423	3.95E-03	2.6E-05	2	1.5	0	1	-86.0
-15	2944	4.50E-03	8.6E-05	1	-1.5	0	1	38.0
-15	3061	4.82E-03	8.6E-05	2	-1.5	0	2	127.5
-15	3101	4.56E-03	5.9E-05	1	-0.5	0	1	38.0
-15	3192	4.90E-03	7.3E-05	2	-0.5	0	2	127.5
-15	3256	4.78E-03	5.9E-05	1	0.5	0	1	38.0
-15	3319	4.93E-03	9.9E-05	2	0.5	0	2	127.5
-15	3405	4.73E-03	8.8E-05	1	1.5	0	1	38.0
-15	3441	4.72E-03	6.5E-05	2	1.5	0	2	127.5
-165	2833	3.85E-03	5.5E-05	1	-1.5	0	2	-17.5
-165	3006	3.90E-03	4.4E-05	1	-0.5	0	2	-17.5
-165	3181	3.95E-03	3.3E-05	1	0.5	0	2	-17.5
-165	3237	4.71E-03	5.4E-05	2	-1.5	0	1	-108.0
-165	3316	4.64E-03	4.6E-05	2	-0.5	0	1	-108.0
-165	3354	4.28E-03	4.2E-05	1	1.5	0	2	-17.5
-165	3388	4.47E-03	3.5E-05	2	0.5	0	1	-108.0
-165	3452	4.28E-03	2.8E-05	2	1.5	0	1	-108.0
-45	2813	3.85E-03	3.8E-05	1	-1.5	0	1	11.0
-45	2990	3.87E-03	4.1E-05	1	-0.5	0	1	11.0
-45	3167	3.95E-03	3.5E-05	1	0.5	0	1	11.0
-45	3285	4.94E-03	4.9E-05	2	-1.5	0	2	102.0
-45	3344	4.34E-03	3.2E-05	1	1.5	1	1	11.0
-45	3394	4.48E-03	3.9E-05	2	0.5	0	2	102.0
-45	3443	4.30E-03	4.3E-05	2	1.5	0	2	102.0
-75	2854	3.20E-03	4.4E-05	1	-1.5	0	1	-22.0
-75	3024	3.21E-03	3.6E-05	1	-0.5	0	1	-22.0
-75	3191	3.35E-03	2.8E-05	1	0.5	1	1	-22.0
-75	3198	3.44E-03	3.0E-05	2	-1.5	1	2	67.0
-75	3288	3.76E-03	3.8E-05	2	-0.5	0	2	67.0
-75	3358	3.42E-03	3.7E-05	1	1.5	0	1	-22.0
-75	3370	3.79E-03	3.4E-05	2	0.5	1	2	67.0
-75	3444	3.67E-03	2.6E-05	2	1.5	0	2	67.0
-90	2953	3.18E-03	3.5E-05	1	-1.5	0	1	-38.0
-90	3057	3.33E-03	3.9E-05	2	-1.5	0	2	51.0
-90	3103	3.21E-03	5.1E-05	1	-0.5	0	1	-38.0
-90	3186	3.39E-03	4.1E-05	2	-0.5	0	2	51.0
-90	3253	3.30E-03	3.5E-05	1	0.5	0	1	-38.0
-90	3312	3.53E-03	3.6E-05	2	0.5	0	2	51.0
-90	3397	3.36E-03	3.3E-05	1	1.5	0	1	-38.0
-90	3428	3.41E-03	3.0E-05	2	1.5	0	2	51.0

Table S30: 10 K single-crystal T_1 anisotropy for 0.1% $Cu(acac)_2$ in $Pd(acac)_2$. Ω indicates sample rod angle in the laboratory frame. Δ indicates 95% confidence interval width. Relative site indexes the lower and higher field Cu contributions at each angle, while absolute site indicates the assignment of

these to sites in the crystal lattice. θ gives the molecular frame angle to B_0 after phase unwrapping. Overlap denotes if two peaks occupy the same field position.								
**Ω (°)**	**B (G)**	**1/T_1 (μs^{-1})**	**Δ 1/T_1 (μs^{-1})**	**Relative site**	**M_I**	**Overlap**	**Absolute site**	**θ (°)**
100	2842	6.05E-05	1.6E-06	1	-1.5	0	2	160.5
100	3012	6.26E-05	2.2E-06	1	-0.5	0	2	160.5
100	3182	6.81E-05	3.6E-06	1	0.5	0	2	160.5
100	3215	5.85E-05	4.9E-06	2	-1.5	0	1	69.0
100	3298	5.78E-05	4.1E-06	2	-0.5	0	1	69.0
100	3351	7.55E-05	6.5E-06	1	1.5	0	2	160.5
100	3373	6.52E-05	3.6E-06	2	0.5	1	1	69.0
100	3440	5.22E-05	1.5E-06	2	1.5	0	1	69.0
135	2816	7.09E-05	1.7E-06	1	-1.5	0	2	192.0
135	2992	7.21E-05	2.0E-06	1	-0.5	0	2	192.0
135	3167	7.59E-05	3.3E-06	1	0.5	0	2	192.0
135	3271	7.17E-05	4.4E-06	2	-1.5	0	1	104.0
135	3333	8.55E-05	4.0E-06	2	-0.5	1	1	104.0
135	3344	9.16E-05	5.7E-06	1	1.5	1	2	192.0
135	3390	8.67E-05	2.4E-06	2	0.5	0	1	104.0
135	3441	6.03E-05	1.5E-06	2	1.5	0	1	104.0
165	2964	7.22E-05	2.8E-06	1	-1.5	0	2	220.5
165	3031	7.21E-05	2.8E-06	2	-1.5	0	1	131.5
165	3116	7.51E-05	3.0E-06	1	-0.5	0	2	220.5
165	3168	7.57E-05	3.5E-06	2	-0.5	0	1	131.5
165	3265	7.94E-05	4.7E-06	1	0.5	0	2	220.5
165	3303	7.82E-05	4.9E-06	2	0.5	0	1	131.5
165	3410	7.87E-05	4.4E-06	1	1.5	0	2	220.5
165	3431	7.28E-05	2.3E-06	2	1.5	1	1	131.5
90	2940	5.87E-05	3.4E-06	1	-1.5	0	1	36.5
90	3065	5.76E-05	4.0E-06	2	-1.5	0	2	128.0
90	3093	6.14E-05	4.0E-06	1	-0.5	0	1	36.5
90	3191	6.14E-05	4.6E-06	2	-0.5	0	2	128.0
90	3244	8.09E-05	7.0E-06	1	0.5	0	1	36.5
90	3313	6.78E-05	6.3E-06	2	0.5	0	2	128.0
90	3391	6.94E-05	6.7E-06	1	1.5	0	1	36.5
90	3427	5.59E-05	2.9E-06	2	1.5	0	2	128.0
75	2872	6.40E-05	1.3E-06	1	-1.5	0	1	25.5
75	3039	6.54E-05	1.5E-06	1	-0.5	0	1	25.5
75	3163	5.95E-05	3.0E-06	2	-1.5	0	2	117.0
75	3202	6.88E-05	2.4E-06	1	0.5	0	1	25.5
75	3261	5.78E-05	2.9E-06	2	-0.5	0	2	117.0
75	3355	6.80E-05	3.1E-06	2	0.5	0	2	117.0
75	3365	7.36E-05	3.9E-06	1	1.5	0	1	25.5
75	3440	5.50E-05	8.0E-07	2	1.5	0	2	117.0
15	2891	7.69E-05	1.2E-06	1	-1.5	0	1	-30.0
15	3056	8.11E-05	1.5E-06	1	-0.5	0	1	-30.0
15	3126	7.68E-05	2.1E-06	2	-1.5	0	2	59.5
15	3220	8.81E-05	2.4E-06	1	0.5	0	1	-30.0
15	3239	7.79E-05	2.2E-06	2	-0.5	0	2	59.5

15	3349	7.99E-05	2.6E-06	2	0.5	0	2	59.5
15	3381	8.52E-05	3.6E-06	1	1.5	0	1	-30.0
15	3450	7.35E-05	7.0E-07	2	1.5	0	2	59.5
45	2804	7.52E-05	6.0E-07	1	-1.5	0	1	5.0
45	2981	7.69E-05	1.1E-06	1	-0.5	0	1	5.0
45	3158	7.95E-05	1.9E-06	1	0.5	0	1	5.0
45	3311	7.74E-05	2.7E-06	2	-1.5	0	2	82.0
45	3336	0.000101	3.4E-06	1	1.5	0	1	5.0
45	3354	8.39E-05	1.6E-06	2	-0.5	1	2	82.0
45	3391	9.44E-05	1.5E-06	2	0.5	0	2	82.0
45	3429	6.64E-05	1.0E-06	2	1.5	0	2	82.0

15. Matlab code for VTVH-T_1 powder anisotropy factor analysis

The following code was used to conduct the T_1 anisotropy factor analysis for powder polycrystalline samples (here, for CuOEP).

```
% CuOEP_modelfree_tempdepT1fitting_MCRALS_vFinal.m
%
% Script for performing soft-modeling of the CuOEP VTVH T1 anisotropy data
% (powder anisotropy) using alternating least-squares.
%
% Nathanael Kazmierczak, 06/19/2024

close all
clear
clc

fns = {'CuOEP_10K_1perc_compiled_errorbars';
    'CuOEP_15K_1perc_allF_errorbars';
    'CuOEP_20K_1perc_allF_errorbars';
    'CuOEP_30K_1perc_allF_errorbars';
    'CuOEP_35K_1perc_allF_errorbars';
    'CuOEP_40K_1perc_allF_errorbars';
    'CuOEP_60K_1perc_allF_errorbars';
    'CuOEP_100K_satrec_witherrorbars';
    'CuOEP_150K_satrec_witherrorbars';
    'CuOEP_290K_invrec_witherrorbars-compiled'};
delete_fields_range = [3425,3440];  % remove fields where organic radicals
arise
DO_SVD = true;
DO_MCRALS = true;
N_ALS_FACTORS = 3;
N_MECHANISMS = 3;
T = [10,15,20,30,35,40,60,100,150,294];
legendtext = {'10 K','15 K','20 K','30 K','35 K','40 K','60 K','100 K','150
K','294 K'};
ANALYSIS_POINT_RANGE = 1:10;
legendtext = legendtext(ANALYSIS_POINT_RANGE);
T = T(ANALYSIS_POINT_RANGE);
fns = fns(ANALYSIS_POINT_RANGE);

%% Load precomputed sin^2theta functional form for initial guess
% The method for computing these functions is detailed in the following
% reference: Kazmierczak, N. P.; Hadt, R. G. J. Am. Chem. Soc. 2022, 144
(45), 20804-20814.
functionsfn = 'CuOEP_35knotscluster_nofigure.mat';
load(functionsfn,'Bgrid','Bused','Bsim','sin2theta_used','sin2thetagrid_stor'
);
sin2theta = sin2theta_used;
sin2theta_grid = sin2thetagrid_stor;
```

```
switch N_ALS_FACTORS
    case 2
        factor_legend = {'1','2'};
    case 3
        factor_legend = {'1','2','3'};
end

%% Load saturation recovery data
% (previously fit to extract spin relaxation rates in the variable
% "storparams")
nf = numel(fns);
stackB = cell(1,nf);
stackStorparams = cell(1,nf);
for i = 1:nf
    clearvars B storparams
    load(fns{i},'B','storparams');
    badinds = B >= delete_fields_range(1) & B <= delete_fields_range(2);
    B(badinds) = [];
    storparams(badinds,:) = [];
    stackB{i} = B;
    stackStorparams{i} = storparams;
end
storT1 = horzcat(stackB',stackStorparams');
allfields = sort(unique([stackB{:}]));  % for interpolation in cases where
there are different B values at different temperatures.
Bused = allfields;

%% Interpolate simulated sin^2theta and saturation recovery data onto
consistent grid
interpinvtau = zeros(numel(Bused),nf);
for i = 1:nf
    interpinvtau(:,i) =
interp1(stackB{i},1000./stackStorparams{i}(:,2),Bused,'linear','extrap');
end
sin2theta = interp1(Bgrid*10,sin2theta_grid,Bused)';

figure;
plot(Bused,sin2theta);
xlabel('B (G)');
ylabel('1/{\itT_1} (norm.)');
title('Computed sin^2\theta functional form');
figure;
plot(Bused,interpinvtau);
xlabel('B (G)');
ylabel('1/{\itT_1} (\mus^{-1})');
title('Unscaled saturation recovery data');

%% Analysis
% For the shapes analysis, fit the anisotropic component of spin relaxation
```

```
%
% First, normalize to the maximum 1/T1 at each temperature, which removes
% the exponential thermal scaling. Now there will be an isotropic and an
% anisotropic component, each with magnitude in the range [0,1].
%
% Next, subtract off the minimum 1/T1, which discards the isotropic
% component of spin relaxation. The magnitude of the remaining anisotropic
% component can beinterpreted as the fraction of spin relaxation that is
% anisotropic at each temperature.
norminvtau = interpinvtau./max(interpinvtau);
norminvtau = norminvtau - min(norminvtau);

figure;
plot(Bused,norminvtau);
legend(legendtext);
xlabel('B (G)');
ylabel('1/{\itT_1} (norm.)');
title('Scaled anisotropic saturation recovery data');

%% Perform SVD analysis to indicate the number of contributing factors
if DO_SVD
    [U,S,V] = svds(norminvtau,7);

    % Singular value scree plot
    figure;
    semilogy(diag(S),'-o');
    xlabel('Factor number');
    ylabel('log singular value');

    % Figure for looking at the factor spectral curves
    figure;
    t = tiledlayout(2,4);
    nexttile;
    plot(Bused,U);
    title('All factors');
    legend('1','2','3','4','5','6','7','Location','northwestoutside');
    xlabel('B (G)');
    ylabel('1/{\itT_1} (norm.)');
    for i = 1:7
        nexttile;
        plot(Bused,U(:,i));
        title(sprintf('Factor %d',i));
        xlabel('B (G)');
        ylabel('1/{\itT_1} (norm.)');
    end
    f = gcf;
    f.Position = [121.8000 341.8000 1.3432e+03 492.0000];
    f.Color = 'w';

    % Figure for looking at the factor contribution profiles
```

```
figure;
t = tiledlayout(2,4);
nexttile;
plot(T',V);
title('All factors');
legend('1','2','3','4','5','6','7','Location','northwestoutside');
xlabel('T (K)');
ylabel('Factor contribution (a.u.)');
for i = 1:7
    nexttile;
    plot(T',V(:,i));
    title(sprintf('Factor %d',i));
    xlabel('T (K)');
    ylabel('Factor contribution (a.u.)');
end
f = gcf;
f.Position = [121.8000 341.8000 1.3432e+03 492.0000];
f.Color = 'w';

% Check data reconstruction for the various rank approximations
figure;
t = tiledlayout(2,4);
nexttile;
plot(Bused,norminvtau);
title('Data');
legend(legendtext,'Location','northwestoutside');
xlabel('B (G)');
ylabel('1/{\itT_1} (norm.)');
for i = 1:7
    nexttile;
    rep = U(:,1:i)*S(1:i,1:i)*(V(:,1:i))';
    plot(Bused,rep);
    title(sprintf('Reconstructed to %dth factor',i));
    xlabel('B (G)');
    ylabel('1/{\itT_1} (norm.)');
end
f = gcf;
f.Position = [121.8000 341.8000 1.3432e+03 492.0000];
f.Color = 'w';

% Plot residuals for various rank approximations
figure;
t = tiledlayout(2,4);
nexttile;
plot(Bused,norminvtau);
title('Data');
legend(legendtext,'Location','northwestoutside');
xlabel('B (G)');
ylabel('1/{\itT_1} (norm.)');
for i = 1:7
```

```
        nexttile;
        rep = U(:,1:i)*S(1:i,1:i)*(V(:,1:i))';
        residuals = norminvtau - rep;
        plot(Bused,residuals);
        title(sprintf('Rank %d residuals',i));
        xlabel('B (G)');
        ylabel('1/{\itT_1} (norm.)');
        ylim([-0.2,0.2]);
    end
    f = gcf;
    f.Position = [121.8000 341.8000 1.3432e+03 492.0000];
    f.Color = 'w';
end


%% Main soft-modeling for the anisotropy shapes
if DO_MCRALS
    D = norminvtau;
    [U,S,V] = svds(norminvtau,N_ALS_FACTORS);
    reference_residuals = D - U*S*V';
    reference_rmsr = sqrt(mean(mean(reference_residuals.^2)))

    % Need initial guesses for the anisotropy factor spectra
    % For initial guess, project the computed sin^2theta functional form
    % onto the SVD factor space.
    projsin2theta = U*U'*sin2theta;

    figure;
    plot(Bused,sin2theta);
    hold on;
    plot(Bused,projsin2theta);
    legend('sin^2\theta','Projected sin^2\theta');
    xlabel('B (G)');
    ylabel('1/{\itT_1} (norm.)');

    % Initialize the full factor spectra guess matrix
    switch N_ALS_FACTORS
        case 2
            Rinit = [projsin2theta,D(:,[1])];
        case 3
            Rinit = [projsin2theta,D(:,[1,3])];
    end
    figure;
    plot(Bused,Rinit);
    xlabel('B (G)');
    ylabel('1/{\itT_1} (norm.)');
    title('Initial factor spectra guesses');

    % Run alternating least squares to refine the factor decomposition
    stillgoing = true;
```

```
maxiter = 1000;
conv_thresh = 1e-10;
count = 1;
this_rmsr = nan;
last_rmsr = nan;
rmsr_storage = zeros(maxiter,1);
R = Rinit;
% Normalize all factor spectra. This way, the contributions from each
% factor will be directly comparable (to each other, and also to the
% isotropic component of spin relaxation, which is the vector of ones)
R = R./max(R);
% ALS loop
while stillgoing
    C = R\D;
    % Enforce nonnegativity on contributions via truncation
    C(C < 0) = 0;
    R = D/C;
    % Enforce nonnegativity on spectra via trunction
    R(R < 0) = 0;
    % Enforce spectra normalization
    R = R./max(R);
    count = count + 1;

    % Check for convergence criteria
    this_rmsr = rms(rms(D - R*C));
    if count > maxiter
        break
    end
    if count > 1
        if abs(this_rmsr - last_rmsr) < conv_thresh
            last_rmsr = this_rmsr;
            break
        end
    end
    last_rmsr = this_rmsr;
    rmsr_storage(i) = this_rmsr;
end
% end ALS

% Compute final data reproduction and residuals
Drep_shapes = R*C;
residuals = D - Drep_shapes;
als_rmsr = sqrt(mean(mean(residuals.^2)))

% Plot the quality of data reproduction
figure;
sp1 = subplot(1,2,1);
plot(Bused,D);
ax1 = gca;
ylval = ax1.YLim;
```

```
    ylim(ylval + 0.05*[-1,1].*ylval);
    xlabel('B (G)');
    ylabel('1/{\itT}_1 (norm.)');
    title('Original data');
    legend(legendtext);
    sp2 = subplot(1,2,2);
    plot(Bused,Drep_shapes);
    ylim(ylval + 0.05*[-1,1].*ylval);
    ax2 = gca;
    title(sprintf('%d-factor ALS reproduced data',N_ALS_FACTORS));
    legend(legendtext);
    xlabel('B (G)');
    ylabel('1/{\itT}_1 (norm.)');
    f = gcf;
    f.Position  = [118.6000 390.6000 1.1744e+03 444.8000];

    % Plot the quality of data fit via shapes for each temperature
    figure
    t = tiledlayout(2,5);
    for i = 1:numel(fns)
        nexttile
        scatter(Bused,D(:,i));
        hold on;
        plot(Bused,Drep_shapes(:,i)); % this is essentially the fit
        if i == 1
            legend('Expt.','Shapes fit','Location','southwest');
        end
        xlabel('B(G)');
        ylabel('1/{\itT}_1 (norm.)');
        title(legendtext{i});
    end
    f = gcf;
    f.Position = [-5.400000000000000 2.474000000000000e+02 1528
5.736000000000000e+02];

    % Plot the R and C factors (including the normalized contribution of
    % the isotropic factor)
    isotropic_vals = min(interpinvtau./max(interpinvtau));
    R = [R,ones(size(R,1),1)];
    C = [C;isotropic_vals];

    figure;
    subplot(1,2,1);
    plot(Bused,R);
    legend([factor_legend,{'constant'}]);
    xlabel('B (G)');
    ylim([-0.05,1.05]);
    ylabel('1/{\itT}_1 (norm.)');
    title('Shapes');
    subplot(1,2,2);
```

```
    plot(T,C);
    legend([factor_legend,{'constant'}]);
    xlabel('T (K)');
    ylabel('Factor contribution');
    title('Shape contributions');
    f = gcf;
    f.Position  = [118.6000 390.6000 1.1744e+03 444.8000];

end

%% Mechanism analysis
% Now that the pure anisotropy shapes have been determined, these shall be
% combined along with the isotropic relaxation into mechanisms. A mechanism
% represents the contribution of a particular vibrational mode, which may
% contain both isotropic and anisotropic components of relaxation. The
% combination of shapes into mechanisms proceeds through solving a least
% squares linear combination problem.

D_nosubtraction = interpinvtau./max(interpinvtau);  % without subtraction of
the isotropic component.

switch N_MECHANISMS
    case 2
        cguess = [2,0,0,1];
        optimfun = @(c)
produceMechanismFromCoefs_costfun_2factor(c,R,D_nosubtraction);
    case 3
        cguess = [2,0,1,0,0.3,0,0,0,0.6];
        optimfun = @(c)
produceMechanismFromCoefs_costfun(c,R,D_nosubtraction);
end
options = optimset('lsqnonlin');
options.MaxFunctionEvaluations = 1e4;
options.MaxFunEvals = 1e4;
options.TolFun = 1e-10;
options.TolX = 1e-10;
options.MaxIter = 1e4;
% Solve the least squares problem
cbest =
lsqnonlin(optimfun,cguess,zeros(size(cguess)),inf(size(cguess)),options);
% obtain the residuals from the best fit
switch N_MECHANISMS
    case 2
        [M,A,mechanism_residuals,mechanism_rmsr] =
produceMechanismFromCoefs_2factor(cbest,R,D_nosubtraction);
    case 3
        [M,A,mechanism_residuals,mechanism_rmsr] =
produceMechanismFromCoefs(cbest,R,D_nosubtraction);
end
mechanism_rmsr
```

```
Drep_mechanisms = M*A;

% Plot the mechanism spectra and contributions
figure;
subplot(1,2,1);
plot(Bused,M);
xlabel('B (G)');
ylim([-0.05,1.05]);
ylabel('1/{\itT}_1 (norm.)');
legend('Mechanism 1','Mechanism 2','Mechanism 3');
title('Mechanisms (isotropic + anisotropic)');
subplot(1,2,2);
plot(T,A);
xlabel('T (K)');
% ylim([-0.05,1.05]);
legend('Mechanism 1','Mechanism 2','Mechanism 3');
title('Factor contributions');
ylabel('Contribution');
f = gcf;
f.Position  = [118.6000 390.6000 1.1744e+03 444.8000];

% Plot the quality of the mechanism fit for each individual spectrum
figure
t = tiledlayout(2,5);
for i = 1:numel(fns)
    nexttile
    scatter(Bused,D_nosubtraction(:,i));
    hold on;
    plot(Bused,Drep_mechanisms(:,i)); % this is essentially the fit
    if i == 1
        legend('Expt.','Mechanism fit','Location','southwest');
    end
    xlabel('B(G)');
    ylabel('1/{\itT}_1  (norm.)');
    title(legendtext{i});
end
f = gcf;
f.Position = [-5.400000000000000 2.474000000000000e+02 1528
5.736000000000000e+02];

% % % % % End main script % % % % %


%% Helper functions

% function [residuals] =
produceMechanismFromCoefs_costfun(c,R,D_nosubtraction)
% % Helper function for forming mechanisms.
% % Nathanael Kazmierczak, 06/19/2024
% [M,A,residuals,rmsr] = produceMechanismFromCoefs(c,R,D_nosubtraction);
```

```
% end

% function [M,A,residuals,rmsr] =
produceMechanismFromCoefs(c,R,D_nosubtraction)
% % Function for combining anisotropy shapes into mechanisms.
% % Nathanael Kazmierczak, 06/19/2024
%
% % construct a positive-valued linear combination of anisotropy shapes +
% % isotropic component to form mechanism spectra M.
% c(c < 0) = 0;
% K = [c(1:3),1;c(4:6),1;c(7:9),1];
% M = R*K';
% M = M./max(M);  % mechanism spectra are normalized, like shapes
% % compute contributions of the mechanism spectra
% A = M\D_nosubtraction;
% A(A < 0) = 0;  % enforce contribution non-negativity through truncation
%
% residuals = D_nosubtraction - M*A;
% rmsr = rms(rms(residuals));
%
% end
```

16. References

(1) Kazmierczak, N. P.; Hadt, R. G. Illuminating Ligand Field Contributions to Molecular Qubit Spin Relaxation via T1 Anisotropy. *J. Am. Chem. Soc.* **2022**, *144* (45), 20804–20814. https://doi.org/10.1021/jacs.2c08729.

(2) Kazmierczak, N. P.; E. Lopez, N.; M. Luedecke, K.; G. Hadt, R. Determining the Key Vibrations for Spin Relaxation in Ruffled Cu(II) Porphyrins via Resonance Raman Spectroscopy. *Chem. Sci.* **2024**, *15* (7), 2380–2390. https://doi.org/10.1039/D3SC05774G.

(3) Hamid, M.; Zeller, M.; Hunter, A. D.; Mazhar, M.; Tahir, A. A. Redetermination of Bis(2,4-Pentanedionato)Palladium(II). *Acta Crystallogr. Sect. E Struct. Rep. Online* **2005**, *61* (11), m2181–m2183. https://doi.org/10.1107/S1600536805030692.

(4) Kazmierczak, N. P.; Luedecke, K. M.; Gallmeier, E. T.; Hadt, R. G. T1 Anisotropy Elucidates Spin Relaxation Mechanisms in an S = 1 Cr(IV) Optically Addressable Molecular Qubit. *J. Phys. Chem. Lett.* **2023**, 7658–7664. https://doi.org/10.1021/acs.jpclett.3c01964.

(5) Kazmierczak, N. P.; Vander Griend, D. A. Properly Handling Negative Values in the Calculation of Binding Constants by Physicochemical Modeling of Spectroscopic Titration Data. *J. Chemom.* **2019**, *33* (11), e3183. https://doi.org/10.1002/cem.3183.

(6) Malinowski, E. R. *Factor Analysis in Chemistry*, 3rd edition.; Wiley: New York, 2002.

(7) Golshan, A.; Abdollahi, H.; Maeder, M. Resolution of Rotational Ambiguity for Three-Component Systems. *Anal. Chem.* **2011**, *83* (3), 836–841. https://doi.org/10.1021/ac102429q.

(8) Wang, Y.-X.; Zhang, Y.-J. Nonnegative Matrix Factorization: A Comprehensive Review. *IEEE Trans. Knowl. Data Eng.* **2013**, *25* (6), 1336–1353. https://doi.org/10.1109/TKDE.2012.51.

(9) de Juan, A.; Casassas, E.; Tauler, R. Soft Modeling of Analytical Data. In *Encyclopedia of Analytical Chemistry*; John Wiley & Sons, Ltd, 2006. https://doi.org/10.1002/9780470027318.a5208.

(10) de Juan, A.; Tauler, R. Multivariate Curve Resolution: 50 Years Addressing the Mixture Analysis Problem – A Review. *Anal. Chim. Acta* **2021**, *1145*, 59–78. https://doi.org/10.1016/j.aca.2020.10.051.

(11) Mason, C.; Maeder, M.; Whitson, A. Resolving Factor Analysis. *Anal. Chem.* **2001**, *73* (7), 1587–1594. https://doi.org/10.1021/ac991141q.

(12) Lebrun, P. C.; Lyon, W. D.; Kuska, H. A. Crystal Structure of Bis(2,4-Pentanedionato)Copper(II). *J. Crystallogr. Spectrosc. Res.* **1986**, *16* (6), 889–893. https://doi.org/10.1007/BF01188194.

(13) Stoll, S.; Schweiger, A. EasySpin, a Comprehensive Software Package for Spectral Simulation and Analysis in EPR. *J. Magn. Reson.* **2006**, *178* (1), 42–55. https://doi.org/10.1016/j.jmr.2005.08.013.

(14) Kiel, A.; Mims, W. B. Paramagnetic Relaxation Measurements on Ce, Nd, and Yb in CaWO4 by an Electron Spin-Echo Method. *Phys. Rev.* **1967**, *161* (2), 386–397. https://doi.org/10.1103/PhysRev.161.386.

(15) Buß, J. H.; Rudolph, J.; Natali, F.; Semond, F.; Hägele, D. Temperature Dependence of Electron Spin Relaxation in Bulk GaN. *Phys. Rev. B* **2010**, *81* (15), 155216. https://doi.org/10.1103/PhysRevB.81.155216.

(16) Nielsen, R. D.; Robinson, B. H. The Spherical Tensor Formalism Applied to Relaxation in Magnetic Resonance. *Concepts Magn. Reson. Part A* **2006**, *28A* (4), 270–290. https://doi.org/10.1002/cmr.a.20055.

(17) Cartoixà, X.; Ting, D. Z.-Y.; Chang, Y.-C. Suppression of the D'yakonov-Perel' Spin-Relaxation Mechanism for All Spin Components in [111] Zincblende Quantum Wells. *Phys. Rev. B* **2005**, *71* (4), 045313. https://doi.org/10.1103/PhysRevB.71.045313.

(18) Levitt, M. H. Chapter 9: Internal Spin Interactions. In *Spin Dynamics: Basics of Nuclear Magnetic Resonance*; Wiley: Chichester, England ; Hoboken, NJ, 2008; p 199.

(19) Thorne, K. S.; Blandford, R. D. 1.3: Tensor Algebra without a Coordinate System. In *Modern Classical Physics: Optics, Fluids, Plasmas, Elasticity, Relativity, and Statistical Physics*; Princeton University Press: Princeton, 2017; p 11.

(20) Neese, F. Software Update: The ORCA Program System—Version 5.0. *WIREs Comput. Mol. Sci.* **2022**, *n/a* (n/a), e1606. https://doi.org/10.1002/wcms.1606.
(21) Szilagyi, R. K.; Metz, M.; Solomon, E. I. Spectroscopic Calibration of Modern Density Functional Methods Using [CuCl4]2-. *J. Phys. Chem. A* **2002**, *106* (12), 2994–3007. https://doi.org/10.1021/jp014121c.
(22) Mirzoyan, R.; Hadt, R. G. The Dynamic Ligand Field of a Molecular Qubit: Decoherence through Spin–Phonon Coupling. *Phys. Chem. Chem. Phys.* **2020**, *22* (20), 11249–11265. https://doi.org/10.1039/D0CP00852D.
(23) Kazmierczak, N. P.; Mirzoyan, R.; Hadt, R. G. The Impact of Ligand Field Symmetry on Molecular Qubit Coherence. *J. Am. Chem. Soc.* **2021**, *143* (42), 17305–17315. https://doi.org/10.1021/jacs.1c04605.
(24) Yen, T. F. *Electron Spin Resonance of Metal Complexes: Proceedings of the Symposium on ESR of Metal Chelates at the Pittsburgh Conference on Analytical Chemistry and Applied Spectroscopy, Held in Cleveland, Ohio, March 4-8, 1968*; Springer US: Boston, MA, 1969.
(25) Maki, A. H.; McGarvey, B. R. Electron Spin Resonance in Transition Metal Chelates. I. Copper (II) Bis-Acetylacetonate. *J. Chem. Phys.* **1958**, *29* (1), 31–34. https://doi.org/10.1063/1.1744456.
(26) Soo, H.; Belford, R. L. Information from Forbidden Hyperfine Lines in Electron Paramagnetic Resonance. Copper Complexes. *J. Am. Chem. Soc.* **1969**, *91* (9), 2392–2394. https://doi.org/10.1021/ja01037a046.
(27) Pilbrow, J. R.; Lowrey, M. R. Low-Symmetry Effects in Electron Paramagnetic Resonance. *Rep. Prog. Phys.* **1980**, *43* (4), 433–495. https://doi.org/10.1088/0034-4885/43/4/002.
