# Supplementary material for: Spectroscopic Signatures of Phonon Character in Molecular Electron Spin Relaxation
Source: ACS Cent Sci. 2024 Dec 11;10(12):2353–62. doi: 10.1021/acscentsci.4c01177 (PMC11672536; doi:10.1021/acscentsci.4c01177)
Supplement: Supplementary file 2 — oc4c01177_si_002.pdf [file oc4c01177_si_002.pdf]

oc-2024-011778.R1

Name: Peer Review Information for "Spectroscopic Signatures of Phonon Character in Molecular Electron Spin Relaxation"

First Round of Reviewer Comments

Reviewer: 1

Comments to the Author

This paper by Kazmierczak et al focuses on understanding a fundamental process relevant to nearly all spin-based applications: spin lattice relaxation. There has been a substantial amount of debate in the literature about this process, and this paper provides a new technique to help settle that debate. They perform orientation-dependence experiments in two ways, one by measuring a powder T1 as a function of field, and then second by measuring a single crystal as a function of rotation in a magnetic field. Together, these measurements allow them to show a crossover point as a function of temperature in the mechanism of spin relaxation in a way that no one has done before. In doing so, they formulate a new way of visualizing spin relaxation with a “spin relaxation tensor” that I think will be an exciting new lens to apply to the challenge of understanding T1. Indeed, a change in the shape of this tensor as a function of temperature was the tell-tale sign of a mechanistic change in T1. I can easily see this paper as “THE” paper for orientation dependence in EPR, and I will certainly point all my students to it. It was a nice change of pace to review a great paper like this and I am happy to suggest it for publication with very minor changes.

I have only two critiques: For one, the characterization of the dilution level seems a little scant. Even if you start with a set stoichiometry, how do you know the crystals are the same stoichiometry as what you started with? Everything about the EPR is so thorough for this paper! I think it would be great to see this section of the experimental a little fleshed out, so that the readers know the authors did the due diligence in sample preparation as well. You have some data from single-crystal measurements, perhaps a few sentences about the dilutions matching previous results, unit cell axes matching what you’d expect for a 1:100 mix, or something like that would be good enough.

Second, I love the saturation v. inversion recovery detail of the paper. I can’t help but wonder to what extent the difference between these measurements is possibly from shot repetition times being inadequately long for the inversion recovery experiments. I think the general prevailing wisdom is that SRT = 5 x T1 is a good choice, though I’ve seen a relatively recent paper

(10.1021/acs.jpcc.2c01090) that suggests SRTs should be 10 x T1 to avoid affecting echo decay shapes. I can't find SRT information in the paper. Maybe I overlooked it. But that should be listed, if not in the experimental, then in figure captions alongside whatever other EPR details once would normally input.

Smaller points:

The arrangement of supporting information section. In the main text, first SI section 12 is discussed and then section 5 and 6 and then 3-4. If the authors can rearrange the section accordingly, it would be great. However, at the present, the SI on its own seems to be correctly arranged.

The computational section is not discussed into the main text. However, as the crystal chosen for the single-crystal EPR is based on the g values obtained through DFT, a couple of lines related to this should go into section 2.3.

Figure S57 and 58, 'mI' should be 'MI' with capital M, I believe.

Reviewer: 2

Comments to the Author

Kazmierczak et al. presents an experimental spectroscopic study of two magnetic molecules -each one defining a spin qubit- based on standard pulse-EPR spectroscopy to elucidate spin-lattice relaxation. During the reviewing process, I found several major concerns -which can be found below- that, in my opinion, preclude publication in ACS Central Science of the manuscript in its current version.

---Minor revisions---

1. Introduction

"In each case, information is encoded through the orientation of the spin,..."

-> Quantum information is not only encoded through the spin orientation (classical information) but also in the relative phase between the two qubits states (phase information).

2. Results

"Figure 2"

-> Caption of Figure 2 should be further elaborated and duplicities should be removed. What are the blue and red arrows? What is B0 in the rightmost C plot?

2.2 Comparison to temperature-dependent T1 fitting

"Yet, as demonstrated, powder VTVH-T1 measurements are able to multiple mechanistic regimes for both..."

-> It seems there is a missing verb. For instance: "Yet, as demonstrated, powder VTVH-T1 measurements are able to unveil multiple mechanistic regimes for both..."

-> On the other hand, given that both the mechanism plot F in Figure 2 and the fitting of T1 vs T for CuOEP with a power law + a molecular vibration are not independent information -since they both are extracted from the same T1 vs T evolution- the claim "suggesting that the mechanism crossover detected is likely the same in both the temperature dimension and the anisotropy dimension." is probably not unexpected. Should it be unexpected in general?

---Major revisions---

**Concerning novelty and impact:

1. Introduction

"The standard measurement has been to determine T1 by pulse EPR at a fixed field position and analyze how it scales as a function of temperature."

-> I am sure there are several old works in the literatura already studying T1 as a function of both the magnitude and the direction of the magnetic field. The authors should compile and cite them.

"New spectroscopic approaches remain called for to clarify between competing hypotheses."

-> I do not see the author's method is sophisticated enough to call it a new spectroscopic approach. It seems rather standard pulse-EPR spectroscopy as all actually boils down to implement well-known pulse sequences to extract T1 at different temperatures and fields. For instance, how does the present manuscript compare to the cited work by Eaton and Eaton (1995) in terms of novelty?

**Concerning how much complementary the presented experimental method is respect to first-principles (computational) calculations:

1. Introduction

"However, developing informative spectroscopic probes for detecting spin relaxation contributions remains an ongoing challenge."

"Yet several different theoretical models can be used to fit these data, many of which do not agree on the underlying mechanism of spin-phonon coupling.12,21–25 Disagreements also exist regarding the character of the molecular vibration and/or lattice phonon modes driving relaxation.16,26 New spectroscopic approaches remain called for to clarify between competing hypotheses. Recently, T1 anisotropy has been introduced as a new spectroscopic observable for probing mechanisms of spin relaxation.27,28"

-> I guess that the mentioned theoretical models are the well-known parametric functions that are fitted to experimental data such as 1/T1 vs Temp. However, we all nowadays have well-developed first-principles computational methods that allows to characterize spin-lattice/phonon relaxation without the need of only relying on the said semi-empirical fitting models. So, what can we get out of the proposed spectroscopic method that we cannot get out of state-of-the-art computational methods? Could not we just rely on current benchmarked first-principles calculations?

3. Discussion

-> Concerning the claim "Powder VTVH-T1 anisotropy contains broad potential to elucidate spin relaxation regimes not accessible by conventional local mode fitting.", the state of the art first-principles models and calculations already show nowadays that the single-mode picture is an oversimplification -being mostly the said mode a rather effective non-existing mode-, meaning that trying to analyze relaxation to suppress it in terms of the said picture can be quite unrealistic, specially when modes are not actually independent one from another within the vibration spectrum (one cannot modify a molecule and to expect that the whole vibration spectrum will not change but at a specific target energy window). Hence, the said calculations can also independently contribute to elucidate those spin relaxation regimes.

**Concerning introduced/employed concepts:

1. Introduction

"For powder samples of both compounds, the shape of the T1 anisotropy changes between the high and low temperature limits, which enables assignment of different regimes of spin Relaxation within a given compound."

-> Which are those regimes? Do we need, for powder samples, to probe T1 anisotropy to learn that there are different spin-relaxation regimes vs temp., or would it be just enough with the thermal dependency of T1?

"VTVH-T1 provides a unique spectroscopic method to ascertain the localized vs. delocalized character of the vibrational modes driving spin relaxation."

-> The separation localized-delocalized is useful yet could actually also be fictitious. How would the proposed spectroscopic method help in case the said separation is not clear? If this is a limitation, it should elaborated on the Discussion section. On the other hand, why is it important to know whether relaxation proceeds either via localized modes or delocalized modes to suppress relaxation? Are the methods to suppress relaxation different depending on the case?

2.1 Powder VTVH-T1

-> The third and fourth paragraphs of this section should be completely rephrased and re-written. It is hard to understand what the authors are doing and conveying. What the "shape analysis" is

useful for? How do the deconvolved shapes are computed? (including a description from scratch understandable for a broad audience and, perhaps, bringing part of the information contained in SI to the main text would be great) Which meaningul physical information do we get out of them? Do each shape have a unique connection to a physical mechanism or are the shapes just an arbitrary mathematical way of deconvolving the measured 1/T1 evolution? For instance, the authors say that the T1*sin^2theta pattern is assigned to the impact of totally symmetric metal-ligand bond stretching vibrations. Can this here be confirmed? On the other hand, what do the authors mean by "mechanism"? How are they determined in plots C and F of Figure 2? One may distinguish between a more or less anisotropic/isotropic mechanism but, which is the actual mechanism? Is it a real physical mechanism or just a renaming of the "shapes"? Are the leftmost B, C, E, F plots determined for some fixed working temperature? Are the rightmost B, C, E, F plots determined for some fixed working field?

2.2 Comparison to temperature-dependent T1 fitting

"Thus, the anisotropy information in VTVH-T1 contains unique mechanistic insights not present in the traditional 1/T1 vs. T fitting approach."

-> Again, the issue is that no information on any physical mechanism is given once one observes the temperature crossover. What are those mechanisms? If we do not have any information on the said mechanisms, how can we propose particular and relevant molecular/lattice modifications to minimize spin-lattice relaxation?

2.3 Single-cristal T1 anisotropy

"Figure 4"

-> D plot. Has this elliptic pattern been published previously in the literature? Has it been found in a different molecule?

-> Captions of F and G plots. Are these claims (see also abstract) found and reported in the literature?

3. Discussion (see also Abstract and 1. Introduction, e.g. "Single-crystal measurements further reveal a change in spin-phonon coupling symmetry from the molecular point group at high temperatures to the crystal packing at low temperatures.")

-> The authors should define precisely what the so-called "spin-relaxation tensor" is. Without even having a precise definition of it, the Discussion section cannot provide a meaningful insight on what is happening nor a trustworthy physical interpretation of the experimental observations. How do the authors know that "At 100 K, the spin relaxation tensor obeys the symmetry of the molecular point group, and the orientation of slowest spin relaxation coincides with the molecular z-axis (Figure 4F)." by lacking a proper definition of the said tensor? Moreover, why "This indicates that the dominant spin-phonon coupling process at 100 K is localized on individual molecules."? Can the authors provide first-principles calculations to verify it and support the correlation between the spin-relaxation tensor orientation/symmetry -respect to the molecular/crystal frame- and the localization/delocalization of the relaxing vibration modes? These questions also apply for the claims made subsequently at 20 K (e.g. "Because the spin relaxation tensor responds to the intermolecular crystal packing rather than the intramolecular bonding, this indicates that the dominant spin-phonon coupling process at 20 K is delocalized across multiple molecules.").

->In particular, since:

"Plots of 1/T1 vs. the laboratory frame orientation of the crystal (Ω) confirm that Ω does not determine 1/T1 at 100 K, while it partially determines 1/T1 at 20 K, and completely determines 1/T1 at 10 K, consistent with a dominant effect of the lattice orientation at low temperatures"

and

"The localized (100 K) and delocalized (20 K) phonon assignments obtained by single-crystal T1 anisotropy correspond to the high- and low-temperature spin relaxation regimes obtained from powder VTVH-T1 (Figure 2C). Therefore, the impact of different atomic motions can be disentangled based on their VTVH-T1 spectroscopic signatures."

are crucial claims for the whole manuscript, they deserve to be confirmed -or not- with first-principles calculations either within the explored molecules or, at least, by employing a made-up toy model. Although even with that confirmation for the particular systems studied, it is still open whether one could find other molecules for which the opposite would be observed: delocalized modes determining relaxation with a spin-relaxation tensor aligned with the molecular frame, and localized modes determining relaxation with a spin-relaxation tensor aligned with the crystal axis. Could this happen?

-> Overall, the concept of symmetry seems to be used vaguely: what does it mean that the symmetry of the spin-relaxation tensor follows either that of the molecular point group or that of the crystal packing? Do the authors particularly refer rather to the orientation of the main axes of the spin-relaxation tensor relative to the molecular plane / a crystal plane? What if using less-symmetric non-planar molecules / crystal packing? How to define the parallel and perpendicular directions of the molecular frame to compare with the main axis of the spin-relaxation tensor? Would the proposed spectroscopic method be useful in these cases? If this could become a limitation, the authors should elaborate on this in the Disussion section.

Reviewer: 3

Comments to the Author

The authors have extended their previous investigation of the anisotropy of the spin-lattice relaxation in S01/2 systems by comparing the behavior of two copper complexes. In EPR information on the anisotropy can also be detected in randomly oriented samples thanks to the selection of a subpopulation of molecular species by selecting the magnetic field resonance. This is commonly done to extract spin hamiltonian anisotropy but can be extended to spin dynamics measurements as in this and previous investigations of the authors on both molecules presented here. See ref. 16, 27, 28.

The novelty here is that they have employed a single crystal of Cu(acac)2 and thus determined the anisotropy more accurately, even if the presence of two molecular orientations in the crystallography cell does not allow for unambiguous assignment.

The take-home message is something that the community has already digested. Low-temperature relaxation is driven by those phonons that have lower energy (the only significantly populated at low temperature), while at high temperature, more efficient local modes get populated and dominate the relaxation process. The former also has a more anisotropic character in energy, as can be seen by any phonon-dispersion plots either computed or measured, even if the authors only quote one of these articles in a different context. For the latter, what dominates is the spin-phonon coupling. The authors could add a simple sentence explaining the sin2tetha dependence instead of the more critical "Analysis of T1 anisotropy collected at 100 K has revealed deficiencies in contemporary spin Hamiltonian theories of spin relaxation, pointing instead to a unique spinorbit wavefunction coupling mechanism.27", especially for a general audience journal.

The investigation has been competently performed and the investigation of T1 anisotropy in single crystals is a powerful technique (though the need of relatively large crystals seems necessary) for better understanding the relaxation mechanism. The manuscript thus appears highly qualified for a more specialized journal such as J. Phys. Chem, but it does not convey the groundbreaking message that is required for an ACS Central publication.

Some comments about the manuscript.

1) Why the authors have not investigated tetraisopropylporphyrin (CuTiPP). In their recent Chem. Sci. these authors have shown that CuTiPP has a much more pronounced anisotropy at low temperature of T_1 (Figure3b/c)? As they have not done any single crystal measurement on the porphyrin molecule, it would be interesting to discuss this molecule as well in this manuscript.

2) The single crystal investigation part needs some improvements: Page 7, line 28. The authors are incorrect here. If the two molecules are reported by the binary axis along b of the P21/n space group, it is not true that the split signal should be observed at any orientation. Indeed along the axis b they are equivalent and according to the SI Figure S41 this direction should be encountered in the rotation.

3) page 7 line 48: Cu(acac)2 does not have two distinct geometries but orientation.

4) Figure 4 should be reported in a clearer way, clarifying also the crystallographic directions and not only the molecular ones. A 3d plot of the complete EPR rotation with the associated points where T1 has been measured would be helpful

5) The manuscript stresses the role of low-energy phonons but does not provide any insight in which is the origin of this anisotropy and which information we can extract beyond the fact that phonons (delocaluized vibrations) are relevant at low temperature. The state-of-the-art experimental and theoretical analysis of low energy phonons can provide their q dependence and anisotropy. A complete picture of the low-energy modes of these simple molecules is within reach and is mandatory for a high-impact publication.

6) Figure 2b-f is particularly difficult to follow.

Author's Response to Peer Review Comments:

Division of Chemistry
and Chemical Engineering

Ryan G. Hadt
1200 E. California Blvd., MC 127-72
Pasadena, CA 91125
(626) 395-6079
rghadt@caltech.edu

Deputy Editor
ACS Central Science

September 29, 2024

Dear Editor,

We greatly appreciate the reviews of our manuscript oc-2024-011778 titled: "Spectroscopic Signatures of Phonon Character in Molecular Electron Spin Relaxation", as well as the opportunity to revise it. Your comments, as well as comments from the reviewers, were helpful, and we believe the corresponding revisions have improved the manuscript.

You will find on the following pages our point-by-point answers to reviewers' remarks. We have also included a copy of the manuscript and supporting information in which all emendations are given in a track changes document for easier identification.

Thank you for considering our research for publication in *ACS Central Science*.

With Best Regards,

Ryan G. Hadt
Assistant Professor of Chemistry
California Institute of Technology

Responses to Reviewers

(Original review comments are printed in black, while author responses are printed in red.)

Editorial Comments:

In addition to addressing the reviewers' detailed comments, I ask that you consider revision of the paper to improve its broad appeal and interest. In particular, I find that the abstract, introduction and conclusions are written more for a specialist reader, and could be improved with respect to making the broader impacts of the work more evident to a non-specialist. This point is important, given that the intent of ACS Central Science is to publish articles that are of broad and general interest.

We thank the Editor for this comment. We have extensively revised the abstract, introduction, and conclusion for better communication to a broad audience. These revisions can be found explicitly in the track changes document of the main text.

Formatting Needs:

AUTHOR AFFILIATION LABELS: Author names and affiliations should be present at the beginning of the manuscript underneath the title. Each affiliation should have a label (for example 1, 2, 3 etc), and the labels should be present by the authors' names.
We have made this change in both the main text and supporting information.

ABSTRACT WORD COUNT: Please make sure the word count does not exceed 200 words.
The new abstract is 185 words in length.

SYNOPSIS MISSING: The synopsis should be no more than 200 characters (including spaces) and should reasonably correlate with the TOC graphic. The synopsis is intended to explain the importance of the article to a broader readership across the sciences. Please place your synopsis in the manuscript file after the TOC graphic, and label it as "Synopsis."
A Synopsis section has been added to the main manuscript file.

Reviewer(s)' Comments to Author:
Reviewer: 1
Recommendation: Publish in ACS Central Science after minor revisions noted.

Comments:
This paper by Kazmierczak et al focuses on understanding a fundamental process relevant to nearly all spin-based applications: spin lattice relaxation. There has been a substantial amount of debate in the literature about this process, and this paper provides a new technique to help settle that debate. They perform orientation-dependence experiments in two ways, one by measuring a powder T1 as a function of field, and then second by measuring a single crystal as a function of rotation in a magnetic field. Together, these

measurements allow them to show a crossover point as a function of temperature in the mechanism of spin relaxation in a way that no one has done before. In doing so, they formulate a new way of visualizing spin relaxation with a "spin relaxation tensor" that I think will be an exciting new lens to apply to the challenge of understanding T1. Indeed, a change in the shape of this tensor as a function of temperature was the tell-tale sign of a mechanistic change in T1. I can easily see this paper as "THE" paper for orientation dependence in EPR, and I will certainly point all my students to it. It was a nice change of pace to review a great paper like this and I am happy to suggest it for publication with very minor changes.

We thank Reviewer 1 for their support of our manuscript.

I have only two critiques: For one, the characterization of the dilution level seems a little scant. Even if you start with a set stoichiometry, how do you know the crystals are the same stoichiometry as what you started with? Everything about the EPR is so thorough for this paper! I think it would be great to see this section of the experimental a little fleshed out, so that the readers know the authors did the due diligence in sample preparation as well. You have some data from single-crystal measurements, perhaps a few sentences about the dilutions matching previous results, unit cell axes matching what you'd expect for a 1:100 mix, or something like that would be good enough.

Reviewer 1 is correct that the stoichiometry of the final crystal could differ from the stoichiometry of the crystal growth solution. However, we can provide an upper bound on the paramagnetic concentration of the single-crystal samples by comparison to the powder concentration dependence series (Supporting Information Section 7). Powdered samples are prepared by fast rotary evaporation, so the nominal stoichiometry should closely match the actual paramagnetic concentration. Indeed, changes in the doubly-integrated intensity of the CW EPR spectra of the powdered samples matched the changes in nominal paramagnetic concentration well. Changes in the peak width in the EDFS for these powdered samples indicates the degree of dipolar broadening to be expected at a given paramagnetic concentration. As shown in Figure S16D, the narrow powder line peak at 3386 G exhibits a full-width-at-half-max of about 8.5 G for the 1:3000 and 1:10 000 dilution levels. This peak broadens to 9.5 G at 1:300, and 13 G at 1:100. By contrast, the single crystal sample possesses a 5 G FWHM for the sharp line centered at 3390 G (Figure 4A), indicating that it is unlikely to be as concentrated as the broadened 1:100 sample. Thus, the single crystal EPR signal is consistent with the nominal single crystal stoichiometry of 1:1000.

We note that if anything, slow co-crystallization of a mixture with a small amount of paramagnetic dopant is likely to exclude the dopant, due to greater fractional solubility. This tends to decrease the paramagnetic concentration of the single crystal. Since spin dynamics are not dependent on paramagnetic concentration in the spin-dilute regime, we are confident that the single crystal results are not adversely affected by paramagnetic concentration.

We have added this discussion to the sample preparation section of the SI (Supporting Information Section 1, page S2).

Second, I love the saturation v. inversion recovery detail of the paper. I can't help but wonder to what extent the difference between these measurements is possibly from shot repetition times being inadequately long for the inversion recovery experiments. I think the general prevailing wisdom is that SRT = 5 x T1 is a good choice, though I've seen a relatively recent paper (10.1021/acs.jpcc.2c01090) that suggests SRTs should be 10 x T1 to avoid affecting echo decay shapes. I can't find SRT information in the paper. Maybe I overlooked

it. But that should be listed, if not in the experimental, then in figure captions alongside whatever other EPR details once would normally input.

The SRT was set to at least 5 times the value of T_1, though in many cases, it was set much longer. For example, Figure S1 displays an example inversion recovery trace with T_1 = 62 μs, but the SRT was set to 5000 μs. Therefore, it is unlikely that the SRT effects cause the difference between saturation recovery and inversion recovery. We believe the more traditional explanation of spectral diffusion contributions to inversion recovery is more probable (see, for example, Ref. 34 in the main text). We have added a description of the SRT criteria to the pulse EPR methods in the SI (Supporting Information Section 2, page 3).

Smaller points:

The arrangement of supporting information section. In the main text, first SI section 12 is discussed and then section 5 and 6 and then 3-4. If the authors can rearrange the section accordingly, it would be great. However, at the present, the SI on its own seems to be correctly arranged.

We have arranged the SI for intelligibility to the reader, rather than according to the sequence of callouts in the main text. In particular, Supporting Information Sections 12-13 (now 13-14 in the revised document) contain a large number of tables. If these sections were moved to the front of the SI, the reader would have to scroll past 15 pages of tables before reaching the description of the experimental methods. We feel the present arrangement of a standalone document will be more helpful to the reader than a chronological presentation.

The computational section is not discussed into the main text. However, as the crystal chosen for the single-crystal EPR is based on the g values obtained through DFT, a couple of lines related to this should go into section 2.3.

We have added a discussion and callout to Supporting Information Section 12 on page 8 of the main text, in Section 2.3.

Figure S57 and 58, ‘mI’ should be ‘MI’ with capital M, I believe.

We have fixed this typographical error.

Reviewer: 2

Recommendation: Reconsider after major revisions noted.

Comments:
Kazmierczak et al. presents an experimental spectroscopic study of two magnetic molecules -each one defining a spin qubit- based on standard pulse-EPR spectroscopy to elucidate spin-lattice relaxation. During the reviewing process, I found several major concerns -which can be found below- that, in my opinion, preclude publication in ACS Central Science of the manuscript in its current version.

We thank Reviewer 2 for their detailed comments on our manuscript. While we have responded to each comment in detail, we believe Reviewer 2's comments can be summarized under three major themes:

1) Reviewer 2 claims that established first-principles calculations can reveal much of the same information content that is contained in our spectroscopic approach. We strongly disagree with this characterization of the theoretical spin relaxation literature. While there exist a plethora of first-principles spin relaxation studies, these papers are rife with disagreement regarding the correct spin-phonon coupling Hamiltonian to employ for S = ½ molecules. Each different Hamiltonian describes the physics leading to spin relaxation differently, consequently predicting qualitatively different vibrational modes to drive spin relaxation. Because there exists no consensus regarding the correct model to pick, first-principles calculations do not currently reveal reliable mechanistic information competitive with our spectroscopic results.

A brief survey of the S = ½ spin relaxation theoretical literature makes this point clear. There exist two main types of S = ½ spin relaxation theories: those employing the spin Hamiltonian for the coupling terms, and those using a non-spin Hamiltonian approach. Of the former, at least four distinct models for spin-phonon coupling have been proposed:

(i) Spin-phonon coupling through the second derivative of the g-tensor (d^2g_i/dQ_2), which predicts *ungerade* bending modes to drive spin relaxation. (Escalera-Moreno, et al., *J. Phys. Chem. Lett.* **2017**, 8, 1695
(ii) Spin-phonon coupling through the first derivative of the full g-tensor ($d\mathbf{g}/dQ$), which predicts ultralow energy rotational modes (Santanni et al., *Inorg. Chem.* **2021**, 60, 140-151).
(iii) Spin-phonon coupling through the first derivative of the principal *g* values (dg_i/dQ), which predicts totally-symmetric metal-ligand stretching modes to drive spin relaxation. (Kazmierczak et al., *J. Am. Chem. Soc.* **2021**, 143, 17305; Kazmierczak et al., *Chem. Sci.* **2024**).
(iv) Spin-phonon coupling through the first derivative of the full hyperfine tensor ($d\mathbf{A}/dQ$), which predicts ultralow energy rotational modes to drive spin relaxation (Lunghi et al., *Sci. Adv.* **2019**, 5, eaax7163; Garlatti et al., *Nat. Commun.* **2023**, 14, 1653).

However, recent EPR spectroscopy results have demonstrated that none of the spin Hamiltonian approaches to relaxation capture the correct mechanism of spin relaxation. In particular, it is known that spin Hamiltonian approaches completely fail to model T_1 anisotropy (Kazmierczak et al., *J. Am. Chem. Soc.* **2022**, 144, 20804), incorrectly model the dependence of T_1 on the strength of the applied magnetic field (Lunghi et al., *Sci. Adv.* **2019**, 5, eaax7163; also Mariano et al., arXiv **2024**, http://arxiv.org/abs/2407.01380), and fail to account for insensitivity to hyperfine changes via isotopic substitution experiments (Mariano et al., arXiv **2024**, http://arxiv.org/abs/2407.01380). Thus, new

theoretical models of spin relaxation have been developed that do not employ the spin Hamiltonian. Of these, there are at least three distinct proposed models for spin-phonon coupling:

(i) Spin-phonon coupling through crystal field modulation of spin-orbit wavefunctions, which predicts totally-symmetric metal-ligand stretching modes to drive spin relaxation (Kazmierczak et al., *J. Am. Chem. Soc.* **2022**, 144, 20804).
(ii) Spin-phonon coupling through non-adiabatic spin-vibrational orbit coupling, which predicts degenerate e_g and e_u modes to drive spin relaxation (Shushkov, *J. Chem. Phys.* **2024**, 160, 164105).
(iii) Spin-phonon coupling through virtual excitations to ligand field excited states, which predicts a variety of low-energy modes to drive spin relaxation (Mariano et al., arXiv **2024**, http://arxiv.org/abs/2407.01380).

Each of these seven models contains a fundamentally different description of the mechanism for S = ½ spin relaxation physics. We can appreciate from this list that new ideas are being developed at a rapid pace, including multiple new contributions in 2024. This list includes both newcomers and established theoreticians; notably, Mariano et al.'s 2024 preprint completely revises the essential relaxation physics as compared to previous papers from the same research group (Lunghi et al., *Sci. Adv.* **2019**, 5, eaax7163; Garlatti et al., *Nat. Commun.* **2023**, 14, 1653). Therefore, while theoretical investigations of spin relaxation are valuable, there does not yet exist a consensus on the correct way to model S = ½ spin relaxation from first principles. There is no substitute for experimental spectroscopy as the most reliable probe of mechanistic information.

We have included an abbreviated, less-technical version of this response on page 2 of the main text in the Introduction. This survey will show a broad audience why computational spin relaxation theories have less reliability than information from experimental spectroscopy.

2) Reviewer 2 appears to have misunderstood the information content extracted from powder VTVH-T_1 anisotropy data, specifically the analysis surrounding Figure 2. Many of Reviewer 2's concerns stem from this root cause. We note that Reviewer 3 also commented on the difficulty of following the analysis in Figure 2. We have therefore significantly revised Figure 2 and the associated discussion to make it more accessible for a broad audience.

3) Reviewer 2 has questioned both the method of extracting the localized vs. delocalized mechanistic separation, as well as the broader value of this mechanistic insight. We have enhanced our discussion of the "spin relaxation tensor" concept to address Reviewer 2's concerns on the technical method. Because of the contradictory state of the computational literature, such spectroscopically-driven mechanistic insights are the main reliable source of information for formulating spin relaxation design principles.

---Minor revisions---

1. Introduction

"In each case, information is encoded through the orientation of the spin,..."

-> Quantum information is not only encoded through the spin orientation (classical information) but also in the relative phase between the two qubits states (phase information).

Reviewer 2 is correct that quantum information is encoded in the qubit phase. However, in the Bloch sphere representation of a qubit state, the phase information can be represented by the azimuthal orientation of the wavefunction vector.

We have revised this sentence on page 1 to read "…information is encoded through the orientation and/or phase of the spin, so any process that dynamically alters the spin orientation on the Bloch sphere will have a deleterious impact on the proposed quantum technology."

2. Results

"Figure 2"

-> Caption of Figure 2 should be further elaborated and duplicities should be removed. What are the blue and red arrows? What is B0 in the rightmost C plot?

We have significantly altered Figure 2, the caption, and the surrounding discussion to address this and other reviewer comments.

The blue and red arrows indicate decreasing and increasing anisotropy as the temperature is reduced, respectively. They serve merely as guides to the eye.

The former "rightmost C plot" is now labeled panel D. In Figure 2D (and also Figure 2F), there is not a single value of B_0. These traces are not single-B_0 cuts out of the panel A data. Instead, these plots show bilinear factor analysis decompositions of the temperature-dependent T_1 anisotropy, which represent the data as the matrix product of T_1 anisotropy shapes vs. B_0 and temperature-dependent contributions vs. T. Thus, the rightmost panel in C tracks the contributions of the *entire curves* in the leftmost panel of C; these curves represent an anisotropy pattern, which spans all possible values of B_0.

We have updated the caption to Figure 2 to emphasize the bilinear nature of the factor analysis decomposition, and we have overhauled the main text discussion of Figure 2. We note that the factor analysis procedure is described in full technical detail in Supporting Information Section 8.

2.2 Comparison to temperature-dependent T1 fitting

"Yet, as demonstrated, powder VTVH-T1 measurements are able to multiple mechanistic regimes for both..."

-> It seems there is a missing verb. For instance: "Yet, as demonstrated, powder VTVH-T1 measurements are able to unveil multiple mechanistic regimes for both..."

We thank the reviewer for catching this typo; we have fixed it accordingly.

-> On the other hand, given that both the mechanism plot F in Figure 2 and the fitting of T1 vs T for CuOEP with a power law + a molecular vibration are not independent information -since they both are extracted from the same T1 vs T evolution- the claim "suggesting that the mechanism crossover detected is likely the same in both the temperature dimension and the anisotropy dimension." is probably not unexpected. Should it be unexpected in general?

On the contrary, Figure 2F and the fitting of T_1 vs. T for CuOEP do indeed provide independent information. The fits in Figure 2F are not extracted from the T_1 vs. T evolution; rather, they are extracted from the evolution of *T_1 anisotropy* vs. T. All T_1 anisotropy data are first normalized by dividing out the average value of T_1 at that temperature, so the T_1 anisotropy data in Figure 2 do not contain information about the average T_1 vs. T evolution. Put differently, the T_1 anisotropy and the T_1 vs. T information can both be extracted from the same pulse EPR dataset we have collected, but we are analyzing these two aspects of the data separately to interrogate the unique information content found in both. This enables us to ascertain the advantage of the variable-temperature T_1 anisotropy methodology.

The independent information content obtained from T_1 anisotropy is the key fact that makes VTVH-T_1 a powerful new spectroscopic approach for spin relaxation. We feel that Reviewer 2 has not appreciated this point, which has led to several Reviewer 2's other comments as well. Our revisions of Figure 2 and the surrounding discussion (pages 3-5) have clarified these points and improved the paper.

---Major revisions---

**Concerning novelty and impact:

1. Introduction

"The standard measurement has been to determine T1 by pulse EPR at a fixed field position and analyze how it scales as a function of temperature."

-> I am sure there are several old works in the literatura already studying T1 as a function of both the magnitude and the direction of the magnetic field. The authors should compile and cite them.

We have already compiled, cited, and discussed several such references in the Discussion section, pages 10-11. These are references 9 and 38-41. It is appropriate to place these remarks in the Discussion section rather than the Introduction because (a) the cited papers do not closely impinge on the VTVH-T_1 methodology we have developed, and (b) it is necessary to keep to Introduction accessible to a broad audience, rather than discussing the minutiae of the older EPR literature.

In this Introduction sentence (page 2), we have replaced "standard" with "most common" to avoid confusion.

"New spectroscopic approaches remain called for to clarify between competing hypotheses."

-> I do not see the author's method is sophisticated enough to call it a new spectroscopic approach. It seems rather standard pulse-EPR spectroscopy as all actually boils down to implement well-known pulse sequences to extract T1 at different temperatures and fields. For instance, how does the present manuscript compare to the cited work by Eaton and Eaton (1995) in terms of novelty?

Our work presents (1) a new experimental design for collecting a 2D spin relaxation dataset, (2) new methods for analyzing this data based on factor analysis and the spin relaxation tensor concept, and (3) new conclusions about spin relaxation mechanisms that cannot be extracted from standard pulse EPR experiments. The combination of these novelties renders this work a new spectroscopic approach.

There are three central novelties to our work that are not present in Ref. 41 (Eaton and Eaton 1995). *First*, we have profiled the complete variation of T_1 against two independent axes, temperature and field, constituting a full 2D dataset that can be represented by a matrix. This has revealed for the first time that T_1 anisotropy changes systematically as a function of temperature, a previously unknown phenomenon. Ref. 41 did not acquire T_1 anisotropy measurements at different temperatures, and so only constitutes a 1D dataset (a vector) not capable of probing anisotropy changes with temperature. *Second*, we analyze our pulse EPR data through a bilinear factor analysis decomposition that allows independent mechanistic information to be extracted from the anisotropy dimension. Ref. 41 does not contain such an analysis, because it does not contain the requisite 2D dataset. As indicated by Reviewer 2's previous comments, Reviewer 2 has not understood the value of this data analysis approach. *Third*, we have acquired variable-temperature anisotropy measurements on single crystal samples and analyzed them through a novel spin relaxation tensor concept. These measurements have revealed an unprecedented change in the symmetry of the spin relaxation tensor. Neither this type of measurement nor this type of analysis exists in Ref. 41.

We have added surface plots of the powder VTVH-T_1 data to the Supporting Information (Figure S18) to emphasize that this is a 2D experimental methodology producing a data matrix, as opposed to traditional vector-valued 1D T_1 data.

**Concerning how much complementary the presented experimental method is respect to first-principles (computational) calculations:

1. Introduction

"However, developing informative spectroscopic probes for detecting spin relaxation contributions remains an ongoing challenge."

"Yet several different theoretical models can be used to fit these data, many of which do not agree on the underlying mechanism of spin-phonon coupling.12,21–25 Disagreements also exist regarding the character of the molecular vibration and/or lattice phonon modes driving relaxation.16,26 New spectroscopic approaches remain called for to clarify between competing hypotheses. Recently, T1 anisotropy has been introduced as a new spectroscopic observable for probing mechanisms of spin relaxation.27,28"

-> I guess that the mentioned theoretical models are the well-known parametric functions that are fitted to experimental data such as 1/T1 vs Temp. However, we all nowadays have well-developed first-principles computational methods that allows to characterize spin-lattice/phonon relaxation without the need of only relying on the said semi-empirical fitting models. So, what can we get out of the proposed spectroscopic method that we cannot get out of state-of-the-art computational methods? Could not we just rely on current benchmarked first-principles calculations?

The theoretical models mentioned are not the parametric functions, but in fact the first-principles computational methods themselves. Contrary to Reviewer 2's assertion, first-principles calculations are rife with disagreement as to the mechanism of spin-lattice relaxation. We refer to our introductory responses to Reviewer 2 on pages 4-5 of this document. We also refer to Figure 1 of Ref. 19 in the main text, and the surrounding discussion in that reference.

It is startling that the reviewer would suggest relying on computation in place of developing new experimental methods. Importantly, given that there are multiple contradictory theoretical approaches in

the literature, we cannot simply rely on first-principles calculations. New mechanistic insights must be obtained from experimental spectroscopy, and the first-principles computational methods must be distinguished on the basis of agreement or disagreement with experiment. By introducing a new spectroscopic observable, our work will enable further benchmarking and discrimination between competing computational models. As such, this work represents an important contribution to the contemporary molecular quantum information science literature seeking to understand the mechanism(s) of spin-phonon coupling and their contribution to spin relaxation processes.

To avoid confusion with the parametric fitting functions, we have replaced the word “fit” with “predict” in the sentence in question on page 2. This section has also been extensively revised to include a discussion of the shortcomings of S = ½ spin relaxation theories.

3. Discussion

-> Concerning the claim "Powder VTVH-T1 anisotropy contains broad potential to elucidate spin relaxation regimes not accessible by conventional local mode fitting.", the state of the art first-principles models and calculations already show nowadays that the single-mode picture is an oversimplification -being mostly the said mode a rather effective non-existing mode-, meaning that trying to analyze relaxation to suppress it in terms of the said picture can be quite unrealistic, specially when modes are not actually independent one from another within the vibration spectrum (one cannot modify a molecule and to expect that the whole vibration spectrum will not change but at a specific target energy window). Hence, the said calculations can also independently contribute to elucidate those spin relaxation regimes.

The referenced sentence is not talking about first-principles calculations, but rather about local mode fitting. Local mode fitting is a parametric fitting approach, and the analysis in Figure 3 demonstrates that local mode fitting cannot access the same information as VTVH-T_1 anisotropy. Thus, our claim is correct as currently written.

Reviewer 2 may be suggesting that we include an additional comparison between the information content from powder VTVH-T_1 anisotropy and first-principles computations. However, as discussed above, S = ½ computational methods are insufficiently reliable to extract this sort of information. Thus, our VTVH-T_1 anisotropy method accesses information content that cannot currently be reliably obtained in any other way.

**Concerning introduced/employed concepts:

1. Introduction

"For powder samples of both compounds, the shape of the T1 anisotropy changes between the high and low temperature limits, which enables assignment of different regimes of spin Relaxation within a given compound."

-> Which are those regimes? Do we need, for powder samples, to probe T1 anisotropy to learn that there are different spin-relaxation regimes vs temp., or would it be just enough with the thermal dependency of T1?

The separation between the regimes is indicated in the revised Figure 2D, 2F, which arises at 36 K and 47 K in each compound. The analysis of $Cu(acac)_2$ presented in the text clearly shows that T_1 anisotropy can

yield unique information not found in the thermal dependence of T_1. The Cu(acac)$_2$ powder sample does not display multiple spin relaxation regimes in the thermal dependence (Figures 3A-B), but it does display multiple regimes in the anisotropy (Figure 3C; also the revised Figure 2C-D).

We have added a sentence at the end of the second-to-last paragraph of Section 2.1 (page 5) that explicitly defines the low- and high-temperature regimes.

"VTVH-T1 provides a unique spectroscopic method to ascertain the localized vs. delocalized character of the vibrational modes driving spin relaxation."

-> The separation localized-delocalized is useful yet could actually also be fictitious. How would the proposed spectroscopic method help in case the said separation is not clear? If this is a limitation, it should elaborated on the Discussion section. On the other hand, why is it important to know whether relaxation proceeds either via localized modes or delocalized modes to suppress relaxation? Are the methods to suppress relaxation different depending on the case?

The localized vs. delocalized separation is obtained directly through analysis of the single-crystal VTVH-T_1 data (Section 2.3) and comparison to the powder VTVH-T_1 regimes (Section 2.1). As discussed at length, delocalization is detected when the fastest and slowest spin relaxation no longer occurs at the principal axes of the g-tensor. There is no limitation to conducting this analysis so long as the requisite data are collected.

Indeed, discrimination between the impact of localized and delocalized phonons does affect the design criteria for suppressing spin relaxation. If spin relaxation dominantly proceeds through localized modes, then the first coordination sphere of the molecule should be altered to suppress relaxation. Relevant strategies have been described in the literature, including stiffening the ligand framework to raise the metal-ligand stretching frequencies and strengthening the ligand field to reduce the orbital contribution to the g value. Conversely, if delocalized modes dominantly drive spin relaxation, then intermolecular contacts / crystal packing effects should exhibit a greater impact. Relevant design criteria are less-well characterized, but may include the symmetry of the space group and the rotational freedom of the molecule in the lattice.

We have added a description of these design principles to the Discussion section on page 11.

2.1 Powder VTVH-T1

-> The third and fourth paragraphs of this section should be completely rephrased and re-written. It is hard to understand what the authors are doing and conveying.

We thank Reviewer 2 for drawing our attention to the difficulty in understanding Figure 2 and the surrounding discussion. We have extensively re-written Section 2.1 as requested, as well as modifying and simplifying Figure 2. Below, we provide a point-by-point response to Review 2's several questions in this paragraph.

- What the "shape analysis" is useful for?
    - To avoid confusion, we have removed the "shape analysis" from the revised Figure 2 and relegated this discussion to the Supporting Information. The "mechanisms analysis" presented in the revised Figure 2 is sufficient for all the conclusions of the main text.

- How do the deconvolved shapes are computed? (including a description from scratch understandable for a broad audience and, perhaps, bringing part of the information contained in SI to the main text would be great)
    - The shapes (mechanisms) are both computed as a bilinear matrix factorization of the spin relaxation dataset. This is a common data analysis procedure in chemistry, used for global analysis of kinetics, titration, chromatography, and time-resolved spectroscopic data. We have added an enhanced description of this process in the main text, and we have also added a schematic to the SI (Figure S17) to explain the matrix factorization procedure graphically to a broad audience.
- Which meaningul physical information do we get out of them? Do each shape have a unique connection to a physical mechanism or are the shapes just an arbitrary mathematical way of deconvolving the measured 1/T1 evolution?
    - The "mechanism" anisotropy patterns presented in the revised Figure 2 correspond to unique physical mechanisms.
- For instance, the authors say that the T1*sin^2theta pattern is assigned to the impact of totally symmetric metal-ligand bond stretching vibrations. Can this here be confirmed?
    - This has already been established in previously published literature. See Kazmierczak, N. P., et al. *J. Am. Chem. Soc.* **2022**, *144* (45), 20804, as well as Kazmierczak, N. P., et al. *J. Phys. Chem. Lett.* **2023**, 7658.
- On the other hand, what do the authors mean by "mechanism"? How are they determined in plots C and F of Figure 2?
    - The mechanisms are determined by the factor analysis procedure described in the text and in Supporting Information Section 8.
- One may distinguish between a more or less anisotropic/isotropic mechanism but, which is the actual mechanism? Is it a real physical mechanism or just a renaming of the "shapes"?
    - These correspond to physical relaxation mechanisms, as described in the revised text.
- Are the leftmost B, C, E, F plots determined for some fixed working temperature? Are the rightmost B, C, E, F plots determined for some fixed working field?
    - No; factor analysis works by representing the primary anisotropy data (revised Figures 2A-B) as the sum of fundamental anisotropy patterns (revised Figures 2C, 2E) that each possess their own unique temperature-dependent contributions (revised Figures 2D, 2F). Each fundamental anisotropy pattern spans across the full range of B_0 values. Thus, the temperature-dependent contributions track the evolution of the entire normalized anisotropy shape (many values of B_0), which is different than extracting the variation of T_1 at a fixed field. Likewise, the anisotropy patterns are extracted from over the entire range of temperature values. We have clarified this point in the main text of Section 2.1, page 5. We have also added a schematic to the SI (Figure S17) that graphically shows how the factor analysis procedure works.

2.2 Comparison to temperature-dependent T1 fitting

"Thus, the anisotropy information in VTVH-T1 contains unique mechanistic insights not present in the traditional 1/T1 vs. T fitting approach."

-> Again, the issue is that no information on any physical mechanism is given once one observes the temperature crossover. What are those mechanisms? If we do not have any information on the said

mechanisms, how can we propose particular and relevant molecular/lattice modifications to minimize spin-lattice relaxation?

Our claim is correct as currently written. The observation of temperature crossover between two mechanisms of relaxation cannot be observed by T_1 thermal dependence for $Cu(acac)_2$, but it can be observed though the change in the T_1 anisotropy. Thus, the powder VTVH-T_1 enable the *detection* of two relaxation mechanisms.

Reviewer 2 appears to be questioning the value of this insight. As discussed previously, the localized vs. delocalized phonon character of the spin relaxation mechanisms can subsequently be identified from the single-crystal T_1 anisotropy patterns (Section 2.3). Thus, single-crystal VTVH-T_1 enables the *assignment* of the two relaxation mechanisms to localized vs. delocalized phonons. Therefore, we do in fact obtain information on both the presence and nature of the spin relaxation.

We have altered paragraph 3 of Section 3 (page 10) to clearly describe this workflow for obtaining mechanistic information.

2.3 Single-cristal T1 anisotropy

"Figure 4"

-> D plot. Has this elliptic pattern been published previously in the literature? Has it been found in a different molecule?

To the best of our knowledge, the elliptic pattern in Figure 4D has not been published previously. It is a key novelty of this study. It indicates that delocalized phonons drive spin relaxation at 20 K, thereby providing mechanistic insight. We have added a phrase on Page 9 to emphasize this novelty.

-> Captions of F and G plots. Are these claims (see also abstract) found and reported in the literature?

To the best of our knowledge, these claims are not reported in the literature. They also constitute a key novelty of this work. This concept of a spin-relaxation tensor that can either be aligned or non-aligned with the *g*-tensor is what enables the assignment of localized vs. delocalized vibrational modes in spin relaxation. As mentioned by Reviewer 1, this concept "will be an exciting new lens to apply to the challenge of understanding T_1". We have modified the beginning of the Discussion section (pages 9-10) and also included language on page S42 to make clear that we are proposing the Cartesian spin relaxation tensor as a novel concept.

3. Discussion (see also Abstract and 1. Introduction, e.g. "Single-crystal measurements further reveal a change in spin-phonon coupling symmetry from the molecular point group at high temperatures to the crystal packing at low temperatures.")

-> The authors should define precisely what the so-called "spin-relaxation tensor" is. Without even having a precise definition of it, the Discussion section cannot provide a meaningful insight on what is happening nor a trustworthy physical interpretation of the experimental observations. How do the authors know that "At 100 K, the spin relaxation tensor obeys the symmetry of the molecular point group, and the orientation of slowest spin relaxation coincides with the molecular z-axis (Figure 4F)." by lacking a proper definition

of the said tensor? Moreover, why "This indicates that the dominant spin-phonon coupling process at 100 K is localized on individual molecules."? Can the authors provide first-principles calculations to verify it and support the correlation between the spin-relaxation tensor orientation/symmetry -respect to the molecular/crystal frame- and the localization/delocalization of the relaxing vibration modes? These questions also apply for the claims made subsequently at 20 K (e.g. "Because the spin relaxation tensor responds to the intermolecular crystal packing rather than the intramolecular bonding, this indicates that the dominant spin-phonon coupling process at 20 K is delocalized across multiple molecules.").

The “spin relaxation tensor" concept is defined by analogy to the *g*-tensor. Just as the *g*-tensor indicates how the Zeeman splitting changes as the magnetic field rotates relative to the molecular frame, so too the “spin relaxation tensor” indicates how the spin relaxation rate changes as the magnetic field rotates relative to the molecular frame. A formal definition is now given in Supporting Information Section 10. The *g*-tensor has principal axes, which indicate the orientations of largest and smallest Zeeman splitting. Likewise, the spin relaxation tensor has principal axes, which indicate the fastest and slowest spin relaxation rates.

The key question is whether the principal axes of the *g*-tensor and spin relaxation tensor align; i.e., does the fastest spin relaxation orientation coincide with the orientation of largest or smallest Zeeman splitting? Because the *g*-tensor axes for $Cu(acac)_2$ align with the first coordination sphere (Supporting Information Section 12), this is equivalent to asking whether the spin relaxation tensor aligns to the molecular coordinate frame. If it does, it is likely that spin relaxation is driven by vibrations that are localized to the first coordination sphere. These localized vibrations have the greatest effect on the *g*-tensor. If the orientations do not coincide, then spin relaxation must be driven by vibrations that are not localized to the first coordination sphere. This is because the spin relaxation orientation dependence does not match the orientation of the first coordination sphere axes or *g*-tensor principal axes.

We have revised the discussion of the spin relaxation tensor on pages 9-10. We have additionally added a new section to the Supporting Information (Section 10: “Discussion of the Spin Relaxation Tensor”; pages S42-S45) to describe the spin relaxation tensor concept with full formal rigor.

->In particular, since:

"Plots of 1/T1 vs. the laboratory frame orientation of the crystal (Ω) confirm that Ω does not determine 1/T1 at 100 K, while it partially determines 1/T1 at 20 K, and completely determines 1/T1 at 10 K, consistent with a dominant effect of the lattice orientation at low temperatures"

and

"The localized (100 K) and delocalized (20 K) phonon assignments obtained by single-crystal T1 anisotropy correspond to the high- and low-temperature spin relaxation regimes obtained from powder VTVH-T1 (Figure 2C). Therefore, the impact of different atomic motions can be disentangled based on their VTVH-T1 spectroscopic signatures."

are crucial claims for the whole manuscript, they deserve to be confirmed -or not- with first-principles calculations either within the explored molecules or, at least, by employing a made-up toy model. Although even with that confirmation for the particular systems studied, it is still open whether one could find other molecules for which the opposite would be observed: delocalized modes determining relaxation with a spin-

relaxation tensor aligned with the molecular frame, and localized modes determining relaxation with a spin-relaxation tensor aligned with the crystal axis. Could this happen?

As discussed in detail above, first-principles calculations are not helpful because of the lack of consensus in the theoretical literature on the mechanism of spin-phonon coupling. However, we appreciate the suggestion to add a toy model to illustrate the origins of this behavior. To that end, we have added a simple model to the Supporting Information (pages S44-S45) showing how delocalized lattice phonons would result in the observed spin relaxation results. By using the spin relaxation tensor concept and applying the mathematics of tensor rotations, we demonstrate an analytical prediction of a $\sin^2(\theta - \varphi)$ T_1 anisotropy functional form (Equation S9). This is the exact anisotropy functional form that is observed at 20 K for the $Cu(acac)_2$ single crystal sample. Thus, our assignments of localized vs. delocalized vibrations are in full agreement with this analytical model.

As this is the first demonstration of the variable-temperature T_1 anisotropy methodology, general conclusions about behavior in other systems are beyond the scope of this study. However, we conjecture that delocalized modes would only determine relaxation aligned with the molecular frame in the event that the relevant lattice planes and the molecular frame are precisely aligned. In such a special case, there is no difference between a spin relaxation tensor aligned with the spin relaxation tensor alignments discussed in the new Figures S61 and S62. Under this conjecture, it may be possible for a delocalized mode to determine relaxation aligned with the molecular frame, but it should not be possible for a localized mode to determine relaxation aligned with the crystal axis. However, further experiments and theory are needed to probe the behavior of variable-temperature T_1 anisotropy in other systems.

We have added the analytical prediction of T_1 anisotropy for delocalized vs. localized relaxation to the new Supporting Information Section 10 (pages 44-45).

-> Overall, the concept of symmetry seems to be used vaguely: what does it mean that the symmetry of the spin-relaxation tensor follows either that of the molecular point group or that of the crystal packing? Do the authors particularly refer rather to the orientation of the main axes of the spin-relaxation tensor relative to the molecular plane / a crystal plane? What if using less-symmetric non-planar molecules / crystal packing? How to define the parallel and perpendicular directions of the molecular frame to compare with the main axis of the spin-relaxation tensor? Would the proposed spectroscopic method be useful in these cases? If this could become a limitation, the authors should elaborate on this in the Disussion section.

As Reviewer 2 correctly states, the "tensor symmetry" refers to the alignment of the principal tensor axes with either the molecular plane or a crystal plane. However, to avoid ambiguity, we have replaced the language of "tensor symmetry" with "tensor alignment" or "tensor orientation" (pages 9-10; also page S42).

In principle, there is no limitation for a molecule with less-than-axial symmetry. Rather than considering parallel and perpendicular orientations, one would then consider the x, y, and z orientations separately, as the three principal tensor axes would all be inequivalent. The localization vs. delocalization analysis would remain the same. If the spin relaxation tensor axes (x, y, and z) fail to align with the *g*-tensor axes (x, y, and z), this would still constitute evidence for a delocalized phonon interaction. We have added a comment on this in the Discussion section on Page 10.

Reviewer: 3

Recommendation: Publish elsewhere J. Phys. Chem.

Comments:
The authors have extended their previous investigation of the anisotropy of the spin-lattice relaxation in S01/2 systems by comparing the behavior of two copper complexes. In EPR information on the anisotropy can also be detected in randomly oriented samples thanks to the selection of a subpopulation of molecular species by selecting the magnetic field resonance. This is commonly done to extract spin hamiltonian anisotropy but can be extended to spin dynamics measurements as in this and previous investigations of the authors on both molecules presented here. See ref. 16, 27, 28. The novelty here is that they have employed a single crystal of Cu(acac)2 and thus determined the anisotropy more accurately, even if the presence of two molecular orientations in the crystallography cell does not allow for unambiguous assignment.

Contrary to Reviewer 3, the novelty of this work extends far beyond the mere use of a single crystal for anisotropy characterization. (1) We have profiled the complete variation of T_1 in powder samples against two independent axes, temperature and field, constituting a full 2D dataset that can be represented by a matrix. This experimental design has not been previously employed. (2) We have developed a factor analysis procedure to extract mechanistic information from VTVH-T_1 data. This technique is novel, and permits unprecedented direct experimental insight into regimes of spin relaxation. (3) We have developed the novel concept of a spin relaxation tensor to explain an unprecedented change in the symmetry of low-temperature relaxation. Single crystal experiments do not merely "determine the anisotropy more accurately" – they unveil a molecular spin relaxation behavior that was previously unknown. These points are also discussed in the responses to Reviewer 2.

We have added a data surface plot in Figure S18 to emphasize the 2D nature of the VTVH-T_1 experimental design. We have added a factor analysis schematic in Figure S17 to show how this 2D nature is essential to the novel data analysis approach developed. We have also clarified the novelty of the spin relaxation tensor concept in the Discussion section, as well as the new Supporting Information Section 10.

The take-home message is something that the community has already digested. Low-temperature relaxation is driven by those phonons that have lower energy (the only significantly populated at low temperature), while at high temperature, more efficient local modes get populated and dominate the relaxation process. The former also has a more anisotropic character in energy, as can be seen by any phonon-dispersion plots either computed or measured, even if the authors only quote one of these articles in a different context. For the latter, what dominates is the spin-phonon coupling. The authors could add a simple sentence explaining the sin2tetha dependence instead of the more critical "Analysis of T1 anisotropy collected at 100 K has revealed deficiencies in contemporary spin Hamiltonian theories of spin relaxation, pointing instead to a unique spinorbit wavefunction coupling mechanism.27", especially for a general audience journal.

The investigation has been competently performed and the investigation of T1 anisotropy in single crystals is a powerful technique (though the need of relatively large crystals seems necessary) for better understanding the relaxation mechanism. The manuscript thus appears highly qualified for a more specialized journal such as J. Phys. Chem, but it does not convey the groundbreaking message that is required for an ACS Central publication.

We thank Reviewer 3 for their appreciation of the technical rigor of our manuscript. We disagree that the findings of this study have already been digested by the spin relaxation community, on three counts:

1) Until now, there has been no experimental spectroscopic method for directly interrogating the character of the phonons causing spin relaxation. Information about spin relaxation has only been inferred indirectly from the temperature dependence of T_1. Yet different classes of phonons produce different anisotropy signatures, which can be used to directly detect competing spin relaxation mechanisms. The realization that spin relaxation anisotropy can serve as a direct spectroscopic probe for phonon character is highly novel, and constitutes a groundbreaking message suitable for *ACS Central Science*.

We have modified the introduction on pages 2-3 to make it clear that the main novelty arises from constructing a direct spectroscopic approach for interrogating phonon character in spin relaxation. We have also added material to the Conclusion (page 12) that re-emphasizes this central novelty for a broad audience.

2) Reviewer 3 suggests that the separation between low-temperature phonon relaxation and high-temperature local mode relaxation is widely accepted. On the contrary, several important recent studies have claimed that low-energy phonons dominate across the entire temperature range of spin relaxation in S = ½ molecules (for example, Garlatti, E., et al. *Nat. Commun.* **2023**, *14*, 1653.; Mariano et al., arXiv **2024**, http://arxiv.org/abs/2407.01380). These studies have rejected the distinction between local modes and low energy phonons, claiming that all relaxation dominantly arises from low-energy phonons. Thus, our work provides an important rebuttal in an ongoing debate. VTVH-T_1 demonstrates the relevance of higher-energy molecular vibrations even for molecules that do not display clear local mode behavior in temperature-dependent T_1 measurements, such as Cu(acac)$_2$. Our work contradicts a view of spin relaxation that is commonly proposed in the contemporary literature.

We have discussed the correspondence between this work and one such low-energy phonon theory in the final paragraph of the Discussion (Section 3, pages 11-12). We also refer to the lack of consensus regarding which vibrations drive spin relaxation in the third paragraph of the Introduction (Section 1, page 2).

3) Reviewer 3 suggests that spin relaxation anisotropy at low temperatures can be simply understood through the energy anisotropy of the phonon dispersion. On the contrary, it is far from obvious whether this simple argument can successfully predict the spin relaxation anisotropy. We are not aware of any theoretical studies employing the phonon dispersion that successfully predict VTVH-T_1 anisotropy behavior. Furthermore, the anisotropic energy in the phonon dispersion referenced by Reviewer 3 exists in momentum space, but this is not the same thing as anisotropy in spin relaxation, which exists due to the real space orientation of the magnetic field. Phonons at all momentum points are populated regardless of the real space orientation of the magnetic field. Thus, it is unclear whether the energy levels of a phonon dispersion translate into anisotropic spin relaxation on their own, and this merits further study by comparison to experiment. By presenting a new spectroscopic observable for spin relaxation, our study opens up an entirely new approach into studying the fundamental process of spin-lattice relaxation.

Some comments about the manuscript.
1) Why the authors have not investigated tetraisopropylporphyrin (CuTiPP). In their recent Chem. Sci. these authors have shown that CuTiPP has a much more pronounced anisotropy at low temperature of T_1 (Figure3b/c)? As they have not done any single crystal measurement on the porphyrin molecule, it would be interesting to discuss this molecule as well in this manuscript.

The CuTiPP molecule is not planar in the crystal structure. In this study, both $Cu(acac)_2$ and copper octaethylporphyrin (CuOEP) are planar in the crystal structure. Since the planarity of a molecule can have significant impacts on its T_1, CuOEP is a better comparison to $Cu(acac)_2$ than CuTiPP. We have added a mention of the planarity of both molecules in the Supporting Information on page S2.

2) The single crystal investigation part needs some improvements: Page 7, line 28. The authors are incorrect here. If the two molecules are reported by the binary axis along b of the P21/n space group, it is not true that the split signal should be observed at any orientation. Indeed along the axis b they are equivalent and according to the SI Figure S41 this direction should be encountered in the rotation.

We thank Reviewer 3 for pointing out this misstatement. Indeed, there exist special orientations of the crystal for which the Cu_A and Cu_B resonance positions are exactly equivalent, and we do encounter cases where the Cu_A and Cu_B resonances are close to coincidence. However, for the vast majority of the crystal directions, the Cu_A and Cu_B signals are widely split.

We have modified the text in the second paragraph of Section 2.3 (page 8) to indicate that the Cu_A and Cu_B split signals are observed at "almost" every orientation, while indicating the special condition that can cause them to coincide.

3) page 7 line 48: Cu(acac)2 does not have two distinct geometries but orientation.

We have changed this wording accordingly.

4) Figure 4 should be reported in a clearer way, clarifying also the crystallographic directions and not only the molecular ones. A 3d plot of the complete EPR rotation with the associated points where T1 has been measured would be helpful.

To clarify the crystallographic directions in Figure 4G, we have replaced the generic hkl labels in Figure 4G with the exact crystal plane axes visualized in the panel. We note that plots of T_1 versus the laboratory-frame angle of the crystal are provided in Supporting Information Section 9, Figures S56-S58. Owing to the near-axial spin Hamiltonian of $Cu(acac)_2$, the crystal was only rotated in one plane, so there is only 2D rotation information to report. This is visualized in these plots of $1/T_1$ versus laboratory frame angle Ω. In addition, a diagram of the rotation axis is given in Figure S46.

5) The manuscript stresses the role of low-energy phonons but does not provide any insight in which is the origin of this anisotropy and which information we can extract beyond the fact that phonons (delocaluized vibrations) are relevant at low temperature. The state-of-the-art experimental and theoretical analysis of low energy phonons can provide their q dependence and anisotropy. A complete picture of the low-energy modes of these simple molecules is within reach and is mandatory for a high-impact publication.

We disagree that contemporary theoretical analysis can reliably model the $S = ½$ spin relaxation anisotropy for low-energy phonons. As discussed at length in the responses to Reviewer 2, there is currently no theoretical consensus of how to model $S = ½$ spin relaxation from first principles, so speculative computational analysis would not significantly add to the value of our spectroscopic insights. We have discussed the shortcomings of current computational models in paragraph 4 of the introduction (Page 2).

6) Figure 2b-f is particularly difficult to follow.

We thank Reviewer 3 for calling our attention to the difficulty in understanding this analysis. We have significantly revamped the presentation of Figure 2 and the surrounding discussion (pages 4-5) in the revised manuscript.

oc-2024-011778.R2

Name: Peer Review Information for "Spectroscopic Signatures of Phonon Character in Molecular Electron Spin Relaxation"

Second Round of Reviewer Comments

Reviewer: 3

Comments to the Author

I thank the authors for the detailed rebuttal letter. I have appreciated the changes made to the text, even if I would have preferred that the annotated copy contains the new parts in a different color, not simply the print of the revision mode of the Word file.

I am still not as enthusiastic as reviewer 1 but not as negative as reviewer 2. Experiments are fundamental, and even if computational methods have significantly advanced, their validation by comparison with experimental observables is a must.

However, in their response, I feel that the authors want to convince us that they have solved an issue. I would say that they provide additional data with which theories of spin-lattice relaxation can be confronted. I would have not doubt accepting this manuscript if it had presented a full picture, including the discrimination of the theoretical models that can better reproduce the new data.

For this reason, I have considered that the take-home message remains a bit technical and better suited for JPC journals, but I am not against its publication in ACS Central Science.

The authors have made a certain effort to make the manuscript more accessible, but some parts remain quite difficult to digest. Take, for instance, the part (G) of the caption of Figure 4.

“(G) T1 anisotropy determined by the long-range orientation of the crystal space group can lead to sin2(θ ± ϕ) angular dependence with different phase shifts ϕ for each crystallographic site, characteristic of delocalized spin-phonon coupling at 20 K.”

They have changed it from the previous version, but what do they mean by ‘long-range orientation’ of a ‘crystal space group’? The authors could ask a colleague working in a related field - but not directly involved in the work - to act as a test reader.

Reviewer: 2

Comments to the Author

I appreciate the author's work at improving their manuscript but, unfortunately, my concerns - specially some of the major ones- have not been removed yet.

-> By following their initial breakdown into three major themes:

regarding 1): First of all, if there is a physical system which has been thoroughly studied over time, that is the one of a spin 1/2 system including its Relaxation within the Open Quantum Systems theory, pioneering J H van Vleck (40's) and R Orbach (60's) and, lately, A Lunghi's (e.g. "Toward exact predictions of spin-phonon relaxation times: An ab initio implementation of open quantum systems theory Sci Adv 8(31) 2022" and related) and L Escalera-Moreno's ("Towards the coherent control of robust spin qubits in quantum algorithms arXiv:2303.12655" with the field magnitude and direction, and temperature, as an input to determine T1) works which are not cited but I encourage to cite.

Moreover, what the authors claim as a set of apparently independent spin-lattice relaxation models which can disagree depending on the selected coupling Hamiltonian is not quite right. The use of different coupling Hamiltonians do not correspond to different independent models leading to a different and irreconcilable description of spin-lattice Relaxation, but rather correspond to different working regimes and Relaxation mechanisms. Depending on whether which regime/mechanism is relevant/important enough, researchers may keep some Hamiltonian terms while dropping others (e.g. see the already-cited paper "How do phonons relax molecular spins?"). E.g., if no field is applied, relaxation would mostly proceed through modulation of the hyperfine tensor A for a spin-1/2 molecular qubit (no need to include the g tensor derivatives here). However, if a field is applied and since the g tensor is much more affected by environmental distortions, one can now drop the A tensor derivatives and keep those of the g tensor. On the other hand, First derivatives are employed in the direct (one-phonon) and real two-phonon processes, while second derivatives appear in the modeling of virtual two-phonon processes (see "Spin-lattice relaxation of individual solid-state spins" and arXiv:2303.12655). So, there is no any initial disagreement or contradiction among the several models, or "lack of consensus on which model to pick", all the said coupling Hamiltonians could be used at once if wanted. The authors should identify which working conditions and regimes/mechanisms apply in their case studies to decide which derivatives must be computed.

All in all and not to be offensive here, I find the author's reason of not performing the requested calculations not knowledgeable enough, thus resulting in a poor excuse. We must not give up on performing calculations just because there may still be discrepancies between theory and experiment in some specific reported case studies. I keep thinking that the request of performing

calculations -as found in my previous report- is legit and that they would significantly contribute to improve this manuscript.

In my opinion and from my experience, the benchmark of an allegedly new experimental method against first-principles calculations is a sine qua non to publish in a high-impact journal such as ACS Cent. Sci. If the authors claim that their experimental method is novel in some sense, they must clearly show what their technique is able to find that current theoretical models cannot. How can we actually know that this experimental technique goes beyond the state of the art -according to authors claim- if the technique is not even tested against the said models for the authors PARTICULAR case studies? The case studies in which the mentioned models fail are not the ones of the authors, are they? Either models fail at reproducing T1 anisotropy or not, there is always a useful output: if models fail, the benchmark is useful at helping to identify what could be wrong with those models, thus providing useful insight to theoreticians; if they do not, the experimental technique can still be useful at probing systems that would be intractable from an ab initio computational point of view if those are too much complex and/or the system size is too large. Is this the authors case?

-> Concerning Figure 2: I find that some of my questions may have not been addressed yet, neither in the caption nor in the main text. The use of the term "anisotropy" is quite confusing. Commonly, anisotropy refers to a function of the spatial direction. However, in this Figure, T1 is measured as a function of two scalar magnitudes, namely field magnitude and temperature in powder samples. I did find a proper description of T1 anisotropy as a function of spatial direction in the supplementary section "Discussion of the spin relaxation tensor". If this single-molecule T1 spatial dependency can be extracted from powder measurements even if they are performed against scalar magnitudes (field magnitude and temperature), then the authors should definitely bring and merge (at least part of) the said supplementary discussion to the main text. Otherwise, it is really confusing to see the term "anisotropy" but no reference to any spatial direction. Moreover, this supplementary discussion where the authors develop an effective anisotropy model is what should be connected with first-principles calculations.

The obtention of plot Fig2A from 2C and 2D (and plot Fig2B from 2E and 2F) seems now to be explained in supplementary section "VTVH-T1 powder anisotropy factor analysis". However, since this method is not widely known, (at least part of) the corresponding supplementary discussion should also be brought and merged to the main text.

The authors explain now that the current D and F plots contain actually the whole set of B0 values in the current C and E plots. However, I do not find this elaborated in the main text. Same for the current C and E plots: if they are not determined at a single temperature, the authors should also elaborate on this in the main text.

On the other hand and concerning again Figure 2 and the discussion below it, it is not clear yet what the authors mean by "mechanism" and which are the actual physical origins for the relaxation mechanisms identified (this is what first-principles calculations would be useful for). Regarding the discussion of Fig2C in the current page 5, the authors claim that mechanism #1 of Cu(acac)2 corresponds to the sen^2\theta anisotropy but no physical origin is mentioned on the mechanism #2 nor on the three mechanisms of CuOEP in Fig2E.

The authors say "At this stage of the analysis, the different regimes can be assigned to relaxation dominated by different classes of phonons. Each phonon mechanism possesses its own characteristic anisotropy pattern, and the temperature dependences arise from thermal population of the relevant phonon modes." They should definitely elaborate this sentences.

On the other hand, according to section 2.2, the authors can here identify the physical origin of the relevant mechanisms for CuOEP ("a power law process dominant at low temperatures, and a molecular vibration dominant at high temperatures") since their powder VTVH-T1 measurements also show a crossover as in the standard fitting of the T1 temperature scaling. However, the same standard fitting fail for Cu(acac)2 so, here, an identification like that is not possible. Yet, the powder VTVH measurements do show that there exist multiple mechanistic regimes but, since the said standard fitting fail, how can now the authors provide a physical interpretation on the origin of those unveiled mechanistic regimes for Cu(acac)2 in terms of phonon/molecular vibration processes? Here, first-principles calculations would again be useful for it and for either to confirm or deny the authors claims in the Discussion section on the spin relaxation tensor. For instance, why the fact that "the spin relaxation tensor aligns to the coordinate frame of the molecular point group, and the orientation of slowest spin relaxation coincides with the molecular z-axis" indicates that "the dominant spin-phonon coupling process at 100 K is localized on individual molecules"? (see Discussion section) Also, why "delocalization is detected when the fastest and slowest spin relaxation no longer occurs at the principal axes of the g-tensor."? (see reply to Reviewer 2) How can I trust these claims without any ab initio calculation? How can I confirm that the guess of the alignment between the spin relaxation tensor and the molecule/lattice symmetry is what is really happening in my case studies -and not any other kind of alingnment/misalignment- and is unequivocally determining the observed functional forms sen^2\theta and sen^2(\theta-\phi)? This is why I was asking whether these claims are found and universally proved in the literature, or whether they are a novel result from the current authors work (but not confirmed/benchmarked for their samples), or whether they are just a case-dependent guess to be confirmed with proper calculations in each case study (that is to say, while the observed sen^2\theta and sen^2(\theta-\phi) functional forms in the authors case studies may be explained with the proposed alignments, there could be different origins behind the experimental behavior of other case studies)

-> Regarding "Our work presents (1) a new experimental design for collecting a 2D spin relaxation dataset, (2) new methods for analyzing this data (...), and (3) new conclusions about spin relaxation mechanisms that cannot be extracted from standard pulse EPR experiments. The combination of these novelties renders this work a new spectroscopic approach." (see reply to Reviewer 2)

I understand that (3) is a consequence of (2). Since (1) is still standard EPR spectroscopy, the core contribution is (2) where the authors do provide -I agree- a novel method to analyze EPR data and get info (not benchmarked though) that could not be always obtained from routinely-employed EPR analysis (such as the regular T1 thermal dependence and its functional fitting).

-> Regarding (see reply to Reviewer 2):

"The analysis of Cu(acac)2 presented in the text clearly shows that T1 anisotropy can yield unique information not found in the thermal dependence of T1. The Cu(acac)2 powder sample does not display multiple spin relaxation regimes in the thermal dependence (Figures 3A-B), but it does display multiple regimes in the anisotropy (Figure 3C; also the revised Figure 2C-D)."

Which meaningul physical information do we get out of them? Do each shape have a unique connection to a physical mechanism or are the shapes just an arbitrary mathematical way of deconvolving the measured 1/T1 evolution? "The “mechanism” anisotropy patterns presented in the revised Figure 2 correspond to unique physical mechanisms."

One may distinguish between a more or less anisotropic/isotropic mechanism but, which is the actual mechanism? Is it a real physical mechanism or just a renaming of the "shapes"?

"These correspond to physical relaxation mechanisms, as described in the revised text."

"The observation of temperature crossover between two mechanisms of relaxation cannot be observed by T1 thermal dependence for Cu(acac)2, but it can be observed though the change in the T1 anisotropy. Thus, the powder VTVH-T1 enable the detection of two relaxation mechanisms."

OK, I think I now understand these points as follows. I asked several times in my previous report for elucidating the physical origin of the said "mechanisms" in terms of vibration/phonon and processes and electronic transitions, but did not get any satisfactory reply. So, as far as I understand, what the authors provide is spectroscopic evidence that something -initially undetected via standard EPR data analysis- is happening in the sample. While this is a really good point, however, no evidence is provided on the actual physical origin of it in terms of the mentioned

processes and transitions. And this is the point where I presume that, as one finds in state-of-the-art works on molecular spin relaxation, one requires the use of first-principles computations to unveil such a dynamic information (population flow, thermalization,...). This information -whether obtained experimentally or theoretically- is undoubtedly a requirement to really claim that one has unveiled the relevant relaxation mechanism.

Summarizing, I would really need to have the whole picture. I understand that performing first-principles calculations may be time-demanding. To make a decision and since half of the whole picture is missing, what I can recommend is to agree with reviewer 3 and publish the authors core contribution (2) in a specialized journal. Then, I would really encourage to prepare another manuscript for a high-impact journal (maybe even in the form of short communication) to publish mostly in parallel, where the whole picture (sketched authors novel method + benchmarking of tensor alignment for localized/delocalized relaxation + dynamic information) is shown with just a single case study, with a simpler language (which I still find tough to follow), and elaborating on how this -now benchmarked- approach allows to go beyond the state of the art by providing a complementary side to both standard EPR data analysis and ab initio calculations. In this case, I would agree with reviewer 1 (e.g. "This concept of a spin-relaxation tensor that can either be aligned or non-aligned with the g-tensor is what enables the assignment of localized vs. delocalized vibrational modes (...). As mentioned by Reviewer 1, this concept “will be an exciting new lens to apply to the challenge of understanding T1”.") and would be happy to accept a manuscript like that in a high-impact journal indeed.

Author's Response to Peer Review Comments:

Division of Chemistry
and Chemical Engineering

Ryan G. Hadt
1200 E. California Blvd., MC 127-72
Pasadena, CA 91125
(626) 395-6079
rghadt@caltech.edu

Deputy Editor ACS Central Science

November 17, 2024

Dear Editor

Please find attached our rebuttal to reviewer comments on our manuscript, "Spectroscopic Signatures of Phonon Character in Molecular Electron Spin Relaxation." We believe that the demands made by Reviewer 2 (a) would significantly damage the appeal of the study to a broad audience, and (b) are unreasonable for the scope of a single study. We justify these claims by explaining the current fluxional state of the spin relaxation theoretical literature. Therefore, we have not included *ab initio* calculations in the revised manuscript.

Given the duration of the review process, we respectfully request a timely indication regarding the acceptability of the rebuttal.

Thank you for consideration of this manuscript; I look forward to hearing from you.

Sincerely,

Ryan G. Hadt
Assistant Professor of Chemistry
California Institute of Technology

Reviewer(s)' Comments to Author:

Reviewer: 3

Recommendation: Publish in ACS Central Science after minor revisions noted.

Comments:

I thank the authors for the detailed rebuttal letter. I have appreciated the changes made to the text, even if I would have preferred that the annotated copy contains the new parts in a different color, not simply the print of the revision mode of the Word file.

I am still not as enthusiastic as reviewer 1 but not as negative as reviewer 2. Experiments are fundamental, and even if computational methods have significantly advanced, their validation by comparison with experimental observables is a must.

However, in their response, I feel that the authors want to convince us that they have solved an issue. I would say that they provide additional data with which theories of spin-lattice relaxation can be confronted. I would have not doubt accepting this manuscript if it had presented a full picture, including the discrimination of the theoretical models that can better reproduce the new data.

For this reason, I have considered that the take-home message remains a bit technical and better suited for JPC journals, but I am not against its publication in ACS Central Science.

We thank Reviewer 3 for their appreciation of our revisions. We remain convinced that our findings are well-suited for publication in ACS Central Science. The topic of spin relaxation is of broad contemporary interest, and the development of new spectroscopic approaches for gaining mechanistic insight is of relevance to synthetic, spectroscopic, and theoretical efforts alike in this field.

The authors have made a certain effort to make the manuscript more accessible, but some parts remain quite difficult to digest. Take, for instance, the part (G) of the caption of Figure 4.

"(G) T1 anisotropy determined by the long-range orientation of the crystal space group can lead to sin2(θ ± ϕ) angular dependence with different phase shifts ϕ for each crystallographic site, characteristic of delocalized spin-phonon coupling at 20 K."

They have changed it from the previous version, but what do they mean by 'long-range orientation' of a 'crystal space group'? The authors could ask a colleague working in a related field - but not directly involved in the work - to act as a test reader.

We thank Reviewer 3 for this additional clarification. The phrase "long-range orientation of the crystal space group" is intended to simply indicate the direction of the lattice planes in the unit cell. These lattice plane orientations arise from the crystal space group rather than the molecular point group. We have modified the text to read "T_1 anisotropy oriented along a lattice plane of the crystal unit cell can lead to $\sin^2(\theta \pm \phi)$ angular dependence with different phase shifts ϕ for each crystallographic site."

Additional Questions:

Quality of experimental data, technical rigor: Top 5%

Significance to chemistry researchers in this and related fields: High

Broad interest to other researchers: Moderate

Novelty: High

Is this research study suitable for media coverage or a First Reactions (a News & Views piece in the journal)?: No

Reviewer: 2

Recommendation: Publish elsewhere specialized journal EPR science

Comments:

I appreciate the author's work at improving their manuscript but, unfortunately, my concerns -specially some of the major ones- have not been removed yet.

While we appreciate Reviewer 2's detailed letter, we strongly disagree with many of their statements. There are two main areas of disagreement. First, Reviewer 2 continues to overestimate the capabilities and maturity of *ab initio* spin relaxation theory. The theoretical literature is in a state of flux, and important contributions in 2024 have cast doubt on the models for which Reviewer 2 advocates.[1,2] Second, as a result, Reviewer 2 underestimates the complexity and technicality that would be introduced by inclusion of *ab initio* calculations. There is no leading *ab initio* model to employ. Instead, there are half a dozen effectively independent models with disparate descriptions of the underlying physics.

Any inclusion of *ab initio* calculations would therefore open up a technical, specialized morass that would damage the broad appeal and impact of this work. If we were to choose a single *ab initio* model for comparison, the choice would be arbitrary, and any of the other *ab initio* models would yield different results. If we were to compare against all of the proposed *ab initio* models, the scope of the study would expand well beyond the confines of a single paper. Further testing of *ab initio* theory against our experimental benchmarks will disentangle the confused theoretical web, but this is not the time and forum to pursue such a multi-year project.

-> By following their initial breakdown into three major themes:

regarding 1): First of all, if there is a physical system which has been thoroughly studied over time, that is the one of a spin 1/2 system including its Relaxation within the Open Quantum Systems theory, pioneering J H van Vleck (40's) and R Orbach (60's) and, lately, A Lunghi's (e.g. "Toward exact predictions of spin-phonon relaxation times: An ab initio implementation of open quantum systems theory Sci Adv 8(31) 2022" and related) and L Escalera-Moreno's ("Towards the coherent control of robust spin qubits in quantum algorithms arXiv:2303.12655" with the field magnitude and direction, and temperature, as an input to determine T1) works which are not cited but I encourage to cite.

We have added these citations on page 2 of the main text to give more context for the present debate over spin relaxation mechanisms.

Moreover, what the authors claim as a set of apparently independent spin-lattice relaxation models which can disagree depending on the selected coupling Hamiltonian is not quite right. The use of different coupling Hamiltonians do not correspond to different independent models leading to a different and irreconcilable description of spin-lattice Relaxation, but rather correspond to different working regimes and Relaxation mechanisms. Depending on whether which regime/mechanism is relevant/important enough, researchers may keep some Hamiltonian terms while dropping others (e.g. see the already-cited paper "How do phonons relax molecular spins?"). E.g., if no field is applied, relaxation would mostly proceed through modulation

of the hyperfine tensor A for a spin-1/2 molecular qubit (no need to include the g tensor derivatives here). However, if a field is applied and since the g tensor is much more affected by environmental distortions, one can now drop the A tensor derivatives and keep those of the g tensor. On the other hand, First derivatives are employed in the direct (one-phonon) and real two-phonon processes, while second derivatives appear in the modeling of virtual two-phonon processes (see "Spin-lattice relaxation of individual solid-state spins" and arXiv:2303.12655). So, there is no any initial disagreement or contradiction among the several models, or "lack of consensus on which model to pick", all the said coupling Hamiltonians could be used at once if wanted. The authors should identify which working conditions and regimes/mechanisms apply in their case studies to decide which derivatives must be computed.

Reviewer 2's characterization of the relationship between spin-lattice relaxation models is flawed. The refutation is found from close examination of Reviewer 2's own words: "Depending on whether which regime/mechanism is relevant/important enough, researchers may keep some Hamiltonian terms while dropping others." The problem is that theory cannot yet predict when these different regimes/mechanisms are most relevant. Thus, the inclusion or omission of certain Hamiltonian terms is in practice quite arbitrary, rendering these different models effectively independent as we originally stated.

We will prove this point by considering the very paper Reviewer 2 mentions, "How do phonons relax molecular spins?"[3] (Lunghi, A.; Sanvito, S. *Sci. Adv.* **2019**, *5* (9), eaax7163). Figure 2 from this paper clearly predicts that the hyperfine interaction should dominate the relaxation not just at zero field, but up to a magnetic field as large as 5 Tesla at 20 K. X-band EPR measurements are conducted at a field of 0.3 Tesla, so this model predicts that hyperfine interactions dominate relaxation at X-band. A more recent work from Lunghi[4] (Garlatti, E, et al. *Nat Commun* **2023**, *14* (1), 1653.) has similarly predicted that hyperfine interactions dominate relaxation at temperatures throughout the Raman relaxation regime (Supplementary Figures 14-17 of that study). Yet this conclusion is in conflict with all available experimental evidence. A large body of experimental EPR work from Gareth and Sandra Eaton, stretching back decades, has clearly established that modulation of the g-tensor / orbital angular momentum is responsible for X-band spin-lattice relaxation, and not the hyperfine interaction.[5–7] This is true across a broad range of different systems, including Cu(II) and Cr(V) coordination complexes and nitroxide radicals. Isotopic substitution experiments on Cr(V) S = ½ compounds are particularly convincing: when moving from ^{53}Cr (I=3/2) to natural abundance Cr (>90% I = 0), the spin relaxation rate does not display appreciable changes, even though the dominant hyperfine interaction has been eliminated.[1,6] So, current theoretical predictions of "which regime/mechanism is relevant/important enough" are simply unreliable.

Alessandro Lunghi, whose work Reviewer 2 references, has himself admitted this in an important 2024 study.[1] Owing to discrepancies with experiment, he abandons his previous spin Hamiltonian approaches based on hyperfine and Zeeman interactions (discussed in the previous paragraph) and posits a new virtual excitation mechanism. He writes[1] (page 1, final paragraph):

> "In particular, none of the fundamental interactions involving a typical spin-1/2 system seems to be able to fully explain its dynamics at temperatures above ~20 K (Raman relaxation), with the Zeeman and the hyperfine interactions failing to explain i) the time-scale of relaxation and the absence of a correlation with the external magnetic field intensity[10, 17], and ii) the angular dependence of relaxation rates[18] and their correlation with spin-orbit coupling strength[19], respectively. Overall, such a state of affairs casts serious doubts on our understanding of this fundamental physical process and must be urgently addressed."[1]

Lunghi is a very respected theorist in this area, and rightfully so, as his works have formed the cutting edge of *ab initio* approaches for spin relaxation. Yet Lunghi's own theories are clearly in flux, and he has

abandoned the former spin Hamiltonian mechanism in favor of a fundamentally distinct, non-spin-Hamiltonian mechanism.

We believe Reviewer 2 does not understand the profound state of flux that currently characterizes spin-lattice relaxation models. No consensus exists for how to choose the correct Hamiltonian under a set of experimental conditions. Thus, the relaxation models we described in our first rebuttal letter do indeed function as independent, competing models.

All in all and not to be offensive here, I find the author's reason of not performing the requested calculations not knowledgeable enough, thus resulting in a poor excuse. We must not give up on performing calculations just because there may still be discrepancies between theory and experiment in some specific reported case studies. I keep thinking that the request of performing calculations -as found in my previous report- is legit and that they would significantly contribute to improve this manuscript.

We certainly have no desire to "give up on performing calculations", as we ourselves have also been involved the development of new theoretical models for spin-lattice relaxation.[8–10] However, inclusion of the requested computations would significantly damage the broad interest of this present study, as we describe below.

In my opinion and from my experience, the benchmark of an allegedly new experimental method against first-principles calculations is a sine qua non to publish in a high-impact journal such as ACS Cent. Sci. If the authors claim that their experimental method is novel in some sense, they must clearly show what their technique is able to find that current theoretical models cannot. How can we actually know that this experimental technique goes beyond the state of the art -according to authors claim- if the technique is not even tested against the said models for the authors PARTICULAR case studies? The case studies in which the mentioned models fail are not the ones of the authors, are they? Either models fail at reproducing T1 anisotropy or not, there is always a useful output: if models fail, the benchmark is useful at helping to identify what could be wrong with those models, thus providing useful insight to theoreticians; if they do not, the experimental technique can still be useful at probing systems that would be intractable from an ab initio computational point of view if those are too much complex and/or the system size is too large. Is this the authors case?

No computational model has predicted the multiple regimes of T_1 anisotropy that we have experimentally found with our new method, nor provided the information content we extract. This is true even though many theoretical studies have investigated these same systems (such as Shushkov's study[2] of Cu(II) porphyrins, including the present case study CuOEP) or very similar systems (such as Lunghi and Sanvito's study[11] of $VO(acac)_2$, whereas we examine the closely-related $Cu(acac)_2$). Reviewer 2 has not disputed this point, yet says this is not enough. Reviewer 2 asserts that to go beyond the state-of-the-art, we must additionally prove that no current computational model *could* be able to predict our results. This is an unreasonable and bizarre demand. Our results are novel because they provide important new information that has not been previously disclosed. Hypotheticals about what theoretical methods could or could not predict are irrelevant to the question of novelty. Theory has not, in point of fact, predicted it; therefore our results are novel and go beyond the state-of-the-art.

Let us enumerate what would be required to satisfy Reviewer 2's demands. To compare to *ab initio* calculations, we must pick a theoretical spin relaxation model. Yet due to the flux in the theoretical literature, there are roughly half a dozen effectively independent models that could be chosen. Which are we to pick, since theory has proven unreliable in indicating which models are appropriate for particular conditions? The choice of any single model is arbitrary, and if disagreement is found, Reviewer 2 could simply claim that we have not chosen the correct model. To do the comparison correctly, we would have to

compare to all competing models simultaneously, and rank the models on the basis of their fidelity to the experimental results. But (1) this enormous undertaking would be vastly beyond the scope of our study, and (2) inclusion of the technical detail needed to explain the differences between the models would severely damage the appeal of our paper for a broad audience. Therefore, such an undertaking should be conducted in separate theoretical papers. The theoretical comparison is suited to specialized, technical journals, while our novel experimental technique is of interest to a broad audience.

-> Concerning Figure 2: I find that some of my questions may have not been addressed yet, neither in the caption nor in the main text. The use of the term "anisotropy" is quite confusing. Commonly, anisotropy refers to a function of the spatial direction. However, in this Figure, T1 is measured as a function of two scalar magnitudes, namely field magnitude and temperature in powder samples. I did find a proper description of T1 anisotropy as a function of spatial direction in the supplementary section "Discussion of the spin relaxation tensor". If this single-molecule T1 spatial dependency can be extracted from powder measurements even if they are performed against scalar magnitudes (field magnitude and temperature), then the authors should definitely bring and merge (at least part of) the said supplementary discussion to the main text. Otherwise, it is really confusing to see the term "anisotropy" but no reference to any spatial direction. Moreover, this supplementary discussion where the authors develop an effective anisotropy model is what should be connected with first-principles calculations.

The extraction of single-molecule T_1 spatial dependence from powder measurements is explained clearly in Figure 1B-E, the associated caption, and the surrounding text. The requested information is already a part of the main text.

The obtention of plot Fig2A from 2C and 2D (and plot Fig2B from 2E and 2F) seems now to be explained in supplementary section "VTVH-T1 powder anisotropy factor analysis". However, since this method is not widely known, (at least part of) the corresponding supplementary discussion should also be brought and merged to the main text.

The method of analysis in Figure 2 is described at an appropriate length in the main text on page 5. Approximately 1.5 paragraphs are already devoted to explaining the technique. Moving further material into the main text from the SI would damage the readability of the paper for a broad audience.

The authors explain now that the current D and F plots contain actually the whole set of B0 values in the current C and E plots. However, I do not find this elaborated in the main text. Same for the current C and E plots: if they are not determined at a single temperature, the authors should also elaborate on this in the main text.

This is already elaborated in the main text on page 5, paragraph 3: "Each fundamental anisotropy pattern spans across the full range of B_0 values. Thus, the temperature-dependent contributions track the evolution of the entire normalized anisotropy shape, which is different than extracting the temperature-dependent variation of T_1 at a fixed field. Likewise, the anisotropy patterns are extracted from over the entire range of temperature values."

On the other hand and concerning again Figure 2 and the discussion below it, it is not clear yet what the authors mean by "mechanism" and which are the actual physical origins for the relaxation mechanisms identified (this is what first-principles calculations would be useful for). Regarding the discussion of Fig2C in the current page 5, the authors claim that mechanism #1 of Cu(acac)2 corresponds to the sen^2\theta anisotropy but no physical origin is mentioned on the mechanism #2 nor on the three mechanisms of CuOEP in Fig2E.

We have added a phrase on Page 5 to clarify that the different mechanisms “aris[e] from the impact of different classes of phonons”.

The authors say "At this stage of the analysis, the different regimes can be assigned to relaxation dominated by different classes of phonons. Each phonon mechanism possesses its own characteristic anisotropy pattern, and the temperature dependences arise from thermal population of the relevant phonon modes." They should definitely elaborate this sentences.

These concepts are already elaborated at length throughout the Results and Discussion sections, as described above.

On the other hand, according to section 2.2, the authors can here identify the physical origin of the relevant mechanisms for CuOEP ("a power law process dominant at low temperatures, and a molecular vibration dominant at high temperatures") since their powder VTVH-T1 measurements also show a crossover as in the standard fitting of the T1 temperature scaling. However, the same standard fitting fail for Cu(acac)2 so, here, an identification like that is not possible. Yet, the powder VTVH measurements do show that there exist multiple mechanistic regimes but, since the said standard fitting fail, how can now the authors provide a physical interpretation on the origin of those unveiled mechanistic regimes for Cu(acac)2 in terms of phonon/molecular vibration processes?

The physical interpretation of the origin of the mechanistic regimes is produced by analysis of the single-crystal anisotropy patterns. This is already described in the main text in several places. For example, the following sentence on page 12: “Powder VTVH-T_1 measurements delineate multiple spin relaxation mechanisms operating in the same compound at different temperatures. Single-crystal VTVH-T_1 measurements characterize the orientation of the spin relaxation tensor, garnering insight into the localized vs. delocalized character of the vibrational/phonon modes coupled to the spin.”

Here, first-principles calculations would again be useful for it and for either to confirm or deny the authors claims in the Discussion section on the spin relaxation tensor. For instance, why the fact that "the spin relaxation tensor aligns to the coordinate frame of the molecular point group, and the orientation of slowest spin relaxation coincides with the molecular z-axis" indicates that "the dominant spin-phonon coupling process at 100 K is localized on individual molecules"? (see Discussion section) Also, why "delocalization is detected when the fastest and slowest spin relaxation no longer occurs at the principal axes of the g-tensor."? (see reply to Reviewer 2) How can I trust these claims without any ab initio calculation? How can I confirm that the guess of the alignment between the spin relaxation tensor and the molecule/lattice symmetry is what is really happening in my case studies -and not any other kind of alingnment/misalignment- and is unequivocally determining the observed functional forms sen^2\theta and sen^2(\theta-\phi)? This is why I was asking whether these claims are found and universally proved in the literature, or whether they are a novel result from the current authors work (but not confirmed/benchmarked for their samples), or whether they are just a case-dependent guess to be confirmed with proper calculations in each case study (that is to say, while the observed sen^2\theta and sen^2(\theta-\phi) functional forms in the authors case studies may be explained with the proposed alignments, there could be different origins behind the experimental behavior of other case studies)

We have developed an analytical theory of the spin relaxation tensor that enables mechanistic conclusions to be drawn from the observed anisotropy patterns (Pages 9-10 and Supporting Information Section 10). This is sufficient support for our claims. Given the state of flux in the *ab initio* theoretical literature (described at length above), inclusion of calculations would not provide additional trustworthy information. In the future, *ab initio* spin-relaxation theory may become reliable enough to confirm experimental interpretation, but the field is not there yet.

-> Regarding "Our work presents (1) a new experimental design for collecting a 2D spin relaxation dataset, (2) new methods for analyzing this data (...), and (3) new conclusions about spin relaxation mechanisms that cannot be extracted from standard pulse EPR experiments. The combination of these novelties renders this work a new spectroscopic approach." (see reply to Reviewer 2)

I understand that (3) is a consequence of (2). Since (1) is still standard EPR spectroscopy, the core contribution is (2) where the authors do provide -I agree- a novel method to analyze EPR data and get info (not benchmarked though) that could not be always obtained from routinely-employed EPR analysis (such as the regular T1 thermal dependence and its functional fitting).

All three points remain important contributions of our work.

-> Regarding (see reply to Reviewer 2):

"The analysis of Cu(acac)2 presented in the text clearly shows that T1 anisotropy can yield unique information not found in the thermal dependence of T1. The Cu(acac)2 powder sample does not display multiple spin relaxation regimes in the thermal dependence (Figures 3A-B), but it does display multiple regimes in the anisotropy (Figure 3C; also the revised Figure 2C-D)."

Which meaningul physical information do we get out of them? Do each shape have a unique connection to a physical mechanism or are the shapes just an arbitrary mathematical way of deconvolving the measured 1/T1 evolution? "The “mechanism” anisotropy patterns presented in the revised Figure 2 correspond to unique physical mechanisms."

One may distinguish between a more or less anisotropic/isotropic mechanism but, which is the actual mechanism? Is it a real physical mechanism or just a renaming of the "shapes"? "These correspond to physical relaxation mechanisms, as described in the revised text."

"The observation of temperature crossover between two mechanisms of relaxation cannot be observed by T1 thermal dependence for Cu(acac)2, but it can be observed though the change in the T1 anisotropy. Thus, the powder VTVH-T1 enable the detection of two relaxation mechanisms."

OK, I think I now understand these points as follows. I asked several times in my previous report for elucidating the physical origin of the said "mechanisms" in terms of vibration/phonon and processes and electronic transitions, but did not get any satisfactory reply. So, as far as I understand, what the authors provide is spectroscopic evidence that something -initially undetected via standard EPR data analysis- is happening in the sample. While this is a really good point, however, no evidence is provided on the actual physical origin of it in terms of the mentioned processes and transitions. And this is the point where I presume that, as one finds in state-of-the-art works on molecular spin relaxation, one requires the use of first-principles computations to unveil such a dynamic information (population flow, thermalization,...). This information -whether obtained experimentally or theoretically- is undoubtedly a requirement to really claim that one has unveiled the relevant relaxation mechanism.

As mentioned above, different mechanisms relate to different classes of phonons participating in relaxation. We have added a phrase on page 5 to clarify this. It is not helpful to conduct first-principles calculations until the state of flux in the spin relaxation theoretical literature has been rectified.

Summarizing, I would really need to have the whole picture. I understand that performing first-principles calculations may be time-demanding. To make a decision and since half of the whole picture is missing, what I can recommend is to agree with reviewer 3 and publish the authors core contribution (2) in a

specialized journal. Then, I would really encourage to prepare another manuscript for a high-impact journal (maybe even in the form of short communication) to publish mostly in parallel, where the whole picture (sketched authors novel method + benchmarking of tensor alignment for localized/delocalized relaxation + dynamic information) is shown with just a single case study, with a simpler language (which I still find tough to follow), and elaborating on how this -now benchmarked- approach allows to go beyond the state of the art by providing a complementary side to both standard EPR data analysis and ab initio calculations. In this case, I would agree with reviewer 1 (e.g. "This concept of a spin-relaxation tensor that can either be aligned or non-aligned with the g-tensor is what enables the assignment of localized vs. delocalized vibrational modes (...). As mentioned by Reviewer 1, this concept "will be an exciting new lens to apply to the challenge of understanding T1".") and would be happy to accept a manuscript like that in a high-impact journal indeed.

Reviewer 2 persists in claiming that our experimental results must be benchmarked against theoretical calculations. When a theoretical field is in a state of flux, this is not the way research should proceed. Instead, it is theoretical calculations that must be benchmarked against experimental results.

Reviewer 2's assertions regarding impact are backwards. Our current manuscript reports a novel experimental approach for clarifying a contentious issue in a hot field; this is the high-impact paper. Multiple subsequent studies will be conducted to benchmark theories against our experimental results, in order to untangle the web of competing models. Those will be technical papers of more narrow interest, suitable for publication in specialized journals. They will all reference our present experimental work as the key benchmark dataset.

Additional Questions:

Quality of experimental data, technical rigor: Top 5%

Significance to chemistry researchers in this and related fields: High

Broad interest to other researchers: High

Novelty: High

Is this research study suitable for media coverage or a First Reactions (a News & Views piece in the journal)?: No

We note that Reviewer 2's assessment of our work's rigor and broad interest has increased substantially over their first review.

References:

(1) Mariano, L. A.; Nguyen, V. H. A.; Petersen, J. B.; Bjørnsson, M.; Bendix, J.; Eaton, G. R.; Eaton, S. S.; Lunghi, A. The Role of Electronic Excited States in the Spin-Lattice Relaxation of Spin-1/2 Molecules. arXiv July 1, 2024. http://arxiv.org/abs/2407.01380 (accessed 2024-07-08).

(2) Shushkov, P. A Novel Non-Adiabatic Spin Relaxation Mechanism in Molecular Qubits. *The Journal of Chemical Physics* **2024**, *160* (16), 164105.

(3) Lunghi, A.; Sanvito, S. How Do Phonons Relax Molecular Spins? *Sci. Adv.* **2019**, *5* (9), eaax7163. https://doi.org/10.1126/sciadv.aax7163.

(4) Garlatti, E.; Albino, A.; Chicco, S.; Nguyen, V. H. A.; Santanni, F.; Paolasini, L.; Mazzoli, C.; Caciuffo, R.; Totti, F.; Santini, P.; Sessoli, R.; Lunghi, A.; Carretta, S. The Critical Role of Ultra-Low-

Energy Vibrations in the Relaxation Dynamics of Molecular Qubits. *Nat Commun* **2023**, *14* (1), 1653. https://doi.org/10.1038/s41467-023-36852-y.
(5) Du, J.-L.; Eaton, G. R.; Eaton, S. S. Temperature and Orientation Dependence of Electron-Spin Relaxation Rates for Bis(Diethyldithiocarbamato)Copper(II). *Journal of Magnetic Resonance, Series A* **1995**, *117* (1), 67–72. https://doi.org/10.1006/jmra.1995.9971.
(6) Du, J. L.; Eaton, G. R.; Eaton, S. S. Electron-Spin-Lattice Relaxation in Natural Abundance and Isotopically Enriched Oxo-Chromium(V)Bis (2-Hydroxy-2-Ethylbutyrate). *Journal of Magnetic Resonance, Series A* **1995**, *115* (2), 236–240. https://doi.org/10.1006/jmra.1995.1172.
(7) Du, J. L.; Eaton, G. R.; Eaton, S. S. Temperature, Orientation, and Solvent Dependence of Electron Spin-Lattice Relaxation Rates for Nitroxyl Radicals in Glassy Solvents and Doped Solids. *Journal of Magnetic Resonance, Series A* **1995**, *115* (2), 213–221. https://doi.org/10.1006/jmra.1995.1169.
(8) Mirzoyan, R.; Hadt, R. G. The Dynamic Ligand Field of a Molecular Qubit: Decoherence through Spin–Phonon Coupling. *Phys. Chem. Chem. Phys.* **2020**, *22* (20), 11249–11265. https://doi.org/10.1039/D0CP00852D.
(9) Kazmierczak, N. P.; Mirzoyan, R.; Hadt, R. G. The Impact of Ligand Field Symmetry on Molecular Qubit Coherence. *J. Am. Chem. Soc.* **2021**, *143* (42), 17305–17315. https://doi.org/10.1021/jacs.1c04605.
(10) Kazmierczak, N. P.; Hadt, R. G. Illuminating Ligand Field Contributions to Molecular Qubit Spin Relaxation via T1 Anisotropy. *J. Am. Chem. Soc.* **2022**, *144* (45), 20804–20814. https://doi.org/10.1021/jacs.2c08729.
(11) Lunghi, A.; Sanvito, S. The Limit of Spin Lifetime in Solid-State Electronic Spins. *J. Phys. Chem. Lett.* **2020**, *11* (15), 6273–6278. https://doi.org/10.1021/acs.jpclett.0c01681.
